# Synthesis of “*All-Cis”* Trihydroxypiperidines from a Carbohydrate-Derived Ketone: Hints for the Design of New β-Gal and GCase Inhibitors

**DOI:** 10.3390/molecules25194526

**Published:** 2020-10-02

**Authors:** Maria Giulia Davighi, Francesca Clemente, Camilla Matassini, Amelia Morrone, Andrea Goti, Macarena Martínez-Bailén, Francesca Cardona

**Affiliations:** 1 Department of Chemistry “Ugo Schiff”, University of Firenze, via della Lastruccia 3-13, 50019 Sesto Fiorentino, Italy; mariagiulia.davighi@unifi.it (M.G.D.); francesca.clemente@unifi.it (F.C.); andrea.goti@unifi.it (A.G.); 2 Paediatric Neurology Unit and Laboratories, Neuroscience Department, Meyer Children’s Hospital and Department of Neurosciences, Pharmacology and Child Health, University of Florence, Viale Pieraccini 24, 50139 Firenze, Italy; amelia.morrone@unifi.it; 3 Associated with Consorzio Interuniversitario Nazionale di Ricerca in Metodologie e Processi Innovativi di Sintesi (CINMPIS), 70100 Bari, Italy; 4 Departamento de Química Orgánica, Facultad de Química, Universidad de Sevilla, García González 1, E-41012 Sevilla, Spain; mmartinez45@us.es

**Keywords:** synthesis, azasugars, iminosugars, lithium acetylides, lysosomal enzyme inhibitors, lysosomal storage disorders (LSDs), lysosomal sphingolipidoses, Gaucher disease

## Abstract

Pharmacological chaperones (PCs) are small compounds able to rescue the activity of mutated lysosomal enzymes when used at subinhibitory concentrations. Nitrogen-containing glycomimetics such as aza- or iminosugars are known to behave as PCs for lysosomal storage disorders (LSDs). As part of our research into lysosomal sphingolipidoses inhibitors and looking in particular for new β-galactosidase inhibitors, we report the synthesis of a series of alkylated azasugars with a relative “*all-cis*” configuration at the hydroxy/amine-substituted stereocenters. The novel compounds were synthesized from a common carbohydrate-derived piperidinone intermediate **8**, through reductive amination or alkylation of the derived alcohol. In addition, the reaction of ketone **8** with several lithium acetylides allowed the stereoselective synthesis of new azasugars alkylated at C-3. The activity of the new compounds towards lysosomal β-galactosidase was negligible, showing that the presence of an alkyl chain in this position is detrimental to inhibitory activity. Interestingly, **9**, **10**, and **12** behave as good inhibitors of lysosomal β-glucosidase (GCase) (IC_50_ = 12, 6.4, and 60 µM, respectively). When tested on cell lines bearing the Gaucher mutation, they did not impart any enzyme rescue. However, altogether, the data included in this work give interesting hints for the design of novel inhibitors.

## 1. Introduction

Lysosomal storage disorders (LSDs) are a group of more than 70 inherited orphan diseases caused by specific mutations in genes encoding lysosomal enzymes and characterized by progressive accumulation of substrates within the lysosomes, leading to organ dysfunctions [1,2,3,4].

Defective activity of lysosomal β-galactosidase (β-Gal), which is responsible for the hydrolytic removal of a terminal β-galactose residue from several glycoconjugates, leads to two different types of rare LSDs, the sphingolipidosis GM1-gangliosidosis and the mucopolysaccharidosis IVB (MPS IVB, also known as Morquio disease type B) [5,6]. Typical substrates of β-Gal are GM1-gangliosides, glycoproteins, oligosaccharides, and glycosaminoglycan keratan sulfate, which accumulate inside the lysosomes due to the malfunctioning enzyme, which usually presents a mutation in its natural amino acid sequence. To date, no pharmacological treatment is available for these severe diseases.

Pharmacological chaperone therapy (PCT) is emerging as a promising therapeutic approach for the treatment of LSDs, especially for those that cannot be treated with enzyme replacement therapy (ERT). PCT is based on pharmacological chaperones (PCs), small molecules that can selectively bind mutant enzymes in the endoplasmic reticulum (ER) and stabilize their correct three-dimensional conformation when used at subinhibitory concentrations, thus improving lysosomal trafficking and rescuing enzymatic activity. PCs are reversible inhibitors and can be replaced by the natural substrate of the enzyme inside the lysosomes. Their main pros include oral administration, broad body distribution (PCs have the potential to cross the blood–brain barrier), and minor side effects [7,8,9,10,11]. 

Nitrogen-containing glycomimetics, such as iminosugars (carbohydrates analogues with a nitrogen atom replacing the endocyclic oxygen), are the most investigated class of PCs for LSDs [12,13].

**Figure 1:**
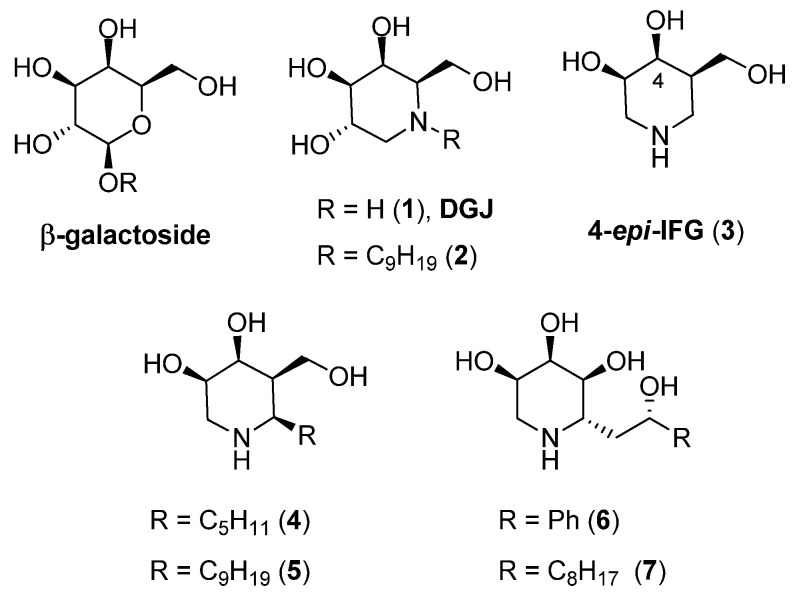
**S**tructures of a general β-galactoside and of some imino- and azasugars inhibitors of β-Gal.

Recently, the first commercially available PC for treating lysosomal Fabry disease, Galafold^TM^ (1-deoxygalactonojirimycin, DGJ, **1**, Figure 1), has been marketed in Europe [14]. Previous studies have shown that the *N*-alkylated derivative of DGJ *N*-nonyl-DGJ (**2**, Figure 1) is able to rescue the intracellular activity of mutant β-Gal in GM1-gangliosidosis patient fibroblasts, thus highlighting the potential of PC therapy for patients with brain pathologies [15,16]. In addition, several azasugars, carbohydrate analogues in which nitrogen formally replaces the anomeric carbon, have shown interesting biological activities, as recently reviewed by Simone and coworkers [17].

In particular, 4-*epi*-isofagomine (**3**, Figure 1) showed moderate inhibition of human lysosomal β-Gal (IC_50_ = 1 μM) [18]. Introduction of an alkyl chain as in compounds **4** and **5** (Figure 1) enhanced the inhibitory potency up to IC_50_ = 10 and IC_50_ = 0.4 nM, respectively. These compounds were able to rescue mutant β-Gal activity in fibroblasts from GM1-gangliosidosis patients [19,20]. Moreover, the “*all-cis*” trihydroxypiperidines **6** and **7** (Figure 1) were able to increase β-Gal activity in GM1 gangliosidosis patient fibroblasts up to 2–6 fold [21].

A commonly employed strategy for the design of a potential PC is to modify a natural inhibitor of the compromised enzyme with the aim of increasing its efficacy and selectivity. However, since the inhibitory potency of a synthetic analogue does not always correlate with its performance as a PC, a reliable structure-activity relationship (SARs) for PCs is not easy and needs to be demonstrated by experimental evidence [21].

**Scheme 1:**
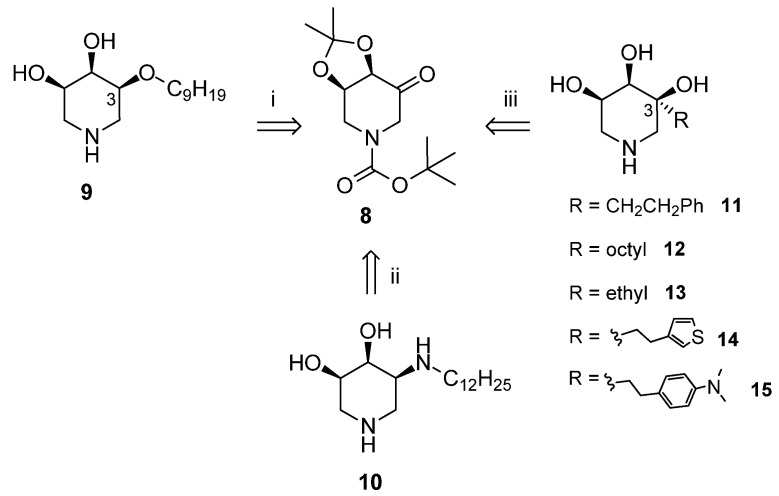
Compounds synthesized in this work through the functionalization of ketone **8** via: (i) reduction to alcohol and Williamson reaction to ether **9**, (ii) reductive amination with dodecyl amine to access **10**, (iii) addition of organolithium derivatives to finally obtain compounds **11**–1**5**.

Based on our previous experience in the synthesis of imino- and azasugars as potential PCs [22,23,24], we envisaged that a new series of *“all-cis*” trihydroxypiperidines and congeners with potential activity as β-Gal inhibitors could be synthesized starting from the common key ketone intermediate **8** [25] (Scheme 1). We report the synthesis of compounds **9**–**15** (Scheme 1) and their assessment as inhibitors of β-Gal and other glycosidases.

Ketone **8** [25] was functionalized through three different synthetic strategies: (i) reduction to the “*all-cis*” alcohol followed by Williamson reaction to afford the ether **9**; (ii) reductive amination of **8** with dodecyl amine provided the new amino azasugar **10**; and (iii) addition of organolithium derivatives followed by proper manipulation allowed access to diversely C-3 functionalized compounds **11**–**15**.

## 2. Results and Discussion

### 2.1. Synthesis

**Scheme 2:**
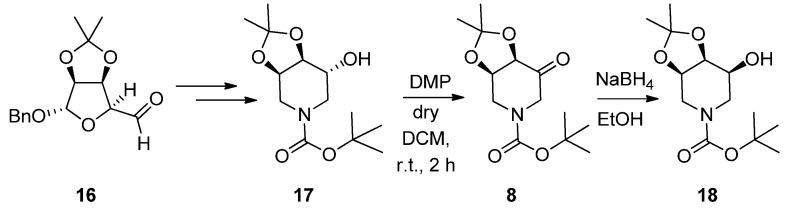
Previous work to synthesize ketone **8** and the alcohols **17** and **18**.

Ketone **8**, precursor of all the new compounds, was synthesized as reported from aldehyde **16**, derived in turn from inexpensive d-mannose in four steps with a high overall yield (85%). The piperidine skeleton of **17** was obtained through a double reductive amination procedure (DRA) [25,26,27] followed by protection of the endocyclic nitrogen atom with a *tert*-butyloxycarbonyl (Boc) group. Oxidation of **17** with Dess Martin periodinane (DMP) gave ketone **8**, which was diastereoselectively reduced to the “*all-cis*” alcohol **18** with NaBH_4_ in EtOH (Scheme 2) [25]. 

**Scheme 3:**
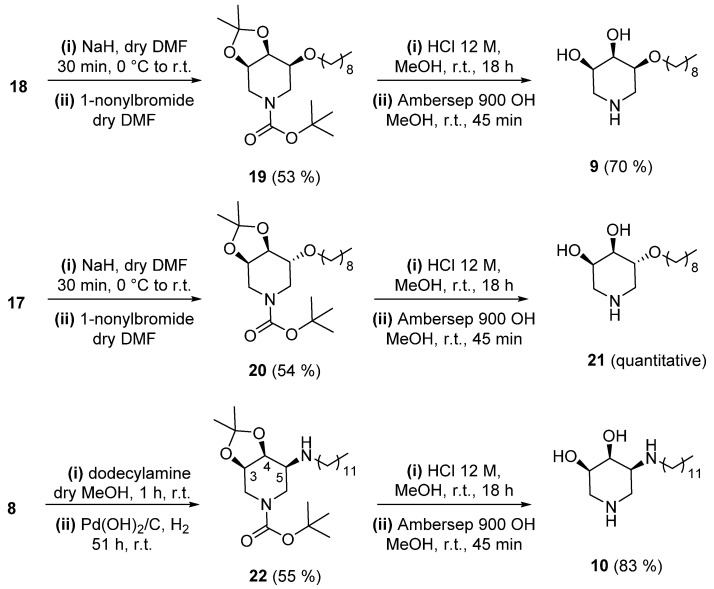
Synthetic strategies to obtain ethers **9** and **21** and the amine **10**.

The “*all-cis*” ether **19** was obtained by Williamson synthesis following a recently reported procedure on a similar substrate [28]. Treatment of alcohol **18** with sodium hydride in dry DMF, alkylation with 1-nonyl bromide gave ether **19** with a 53% yield. Concomitant deprotection of acetonide and Boc groups under acidic conditions (aqueous HCl in MeOH), followed by treatment with the strongly basic resin Ambersep 900 OH, gave ether **9** with a 70% yield (Scheme 3). In order to investigate the role of different configurations at C-3 on biological activity, the diastereomeric alcohol **17** was treated similarly to give protected ether **20** with a 54% yield. Deprotection of **20** as above yielded ether **21** quantitatively (Scheme 3).

Ketone **8** was also converted to the amine **22** through reductive amination employing dodecyl amine. The reaction was performed in dry MeOH under catalytic hydrogenation on Pd(OH)_2_/C and gave, after purification by flash column chromatography (FCC), the protected piperidine **22** with a 55% yield. Final deprotection under acidic conditions followed by treatment with Ambersep 900 OH resin gave the free amine **10** with an 83% yield (Scheme 3). The “*all-cis*” configuration in compound **22** was established by comparison of its ^1^H-NMR spectrum with those of similar compounds with a different alkyl chain at the exocyclic nitrogen atom [29]. The broad singlet at 4.41 ppm observed for 4-H in the ^1^H-NMR spectrum of **22** is consistent with eq-ax relationships with both 3-H and 5-H occurring in its chair conformations.

**Scheme 4:**
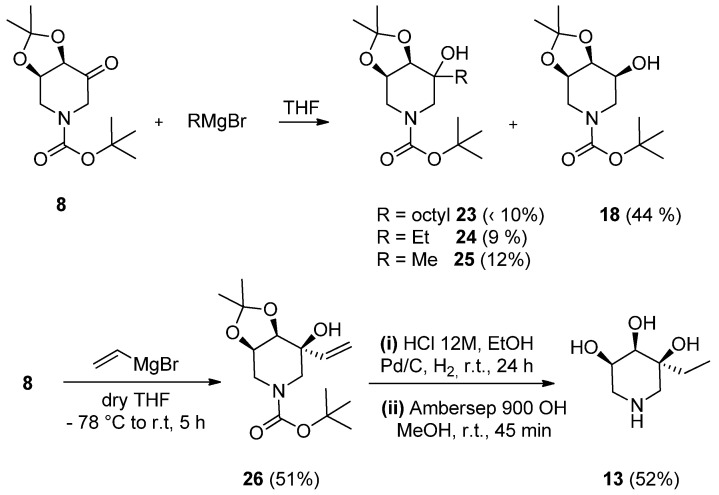
Addition reactions of sp^3^ and sp^2^ Grignard reagents to ketone **8** and synthesis of **13**.

The synthesis of “*all-cis*” trihydroxypiperidines alkylated at C-3 by addition of organometallic reagents to the carbonyl group of **8** was then addressed. Ketone **8** was first reacted with Grignard reagents (octylMgBr, ethylMgBr, and methylMgBr, Scheme 4) under conditions which had proved successful for Grignard additions to aldehyde **16** and a nitrone derived thereof [23,30]. 

The addition of ethyl magnesium bromide to a simpler *N*-Boc-protected 4-piperidone has been previously reported [31]. However, Grignard additions to ketone **8** proved difficult and sluggish. Variation of reaction conditions (temperature ranging from −78 °C to room temperature, Grignard equivalents ranging from 1 to 1.8, see the Supplementary Materials for further details) did not lead to substantial improvements, and the desired alcohols **23**, **24,** or **25** were obtained with yields of 12% or less, in complex mixtures difficult to purify. In case of **23** and **24**, a single diasteromeric adduct was isolated (as assessed by ESI-MS and ^1^H-NMR), while with methyl magnesium bromide a mixture of two diastereoisomers (about 12% yield) was observed in the ^1^H-NMR spectrum. The low yields can be ascribed to formation of the reduction product **18**. Reduction of ketones by Grignard reagents through a β-hydride addition is known as an undesired side reaction that can occur with particularly encumbered substrates as a consequence of the reduced rate of nucleophilic addition [32,33]. Formation of **18** was attested by analysis of the ^1^H-NMR and ESI-MS spectra and confirmed after acetylation of the free OH group (see the Supplementary Materials) (Scheme 4) in case of octyl magnesium bromide addition.

Given the poor results obtained with alkyl Grignard reagents, we investigated the addition reaction with less bulky sp^2^ and sp organometals. The reaction of **8** with vinyl magnesium bromide was less sluggish and a single diastereoisomeric adduct **26** (see below for structural assignment) was obtained with a 51% yield (Scheme 4). Hydrogenation of **26** in the presence of Pd/C under acidic conditions (aqueous HCl in EtOH) allowed complete deprotection and reduction of the alkene. Final treatment with Ambersep 900 OH and purification by FCC gave the saturated azasugar **13** with a 52% yield (Scheme 4).

Encouraged by this result, we turned our attention to the addition of alkynyl organometallic reagents, namely, lithium acetylides generated in situ by treating terminal alkynes with butyl lithium. Compared to many C-nucleophiles, acetylides are less basic and less sensitive to steric congestion [34]. The results of the addition of structurally differentiated lithium acetylides to ketone **8** are reported in Table 1

**Scheme 5:**
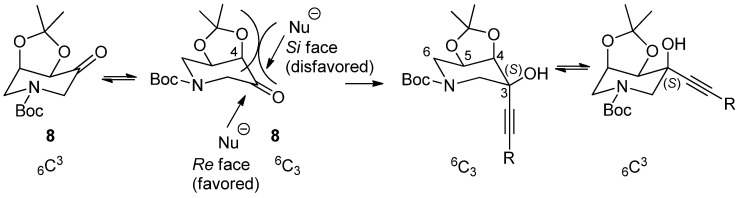
Stereochemical outcome of the addition of lithium acetylides to ketone **8**.

The alkynes were treated with BuLi (1.5 equiv.) at −78 °C for 30 min, followed by the addition of ketone **8** at −78 °C. The reaction mixture was allowed to warm to room temperature and left to react for 2.5–3 h, then worked up. In all reported cases, FCC of the crude mixtures afforded good yields (65–88%) of adducts with both simple and functionalized alkenes (Table 1). A single diastereoisomer was obtained in all cases, which was ascribed the (*S*) configuration at the newly formed C-3 stereocenter on the basis of the following considerations. Alkynyl lithium derivatives are small nucleophiles, which typically prefer an axial rather than an equatorial attack on cyclohexanones [35,36,37] in order to avoid torsional strain [38,39]. In Scheme 5, the two more stable chair conformations of ketone **8** are depicted. Only in the ^6^C_3_ conformation, nucleophilic axial attack at C-3 experiences a stabilizing interaction by the low-lying energy σ* orbital of the antiperiplanar C-O bond at C-4, according to a favorable Felkin–Anh model [40,41]. Thus, based on stereoelectronic and steric considerations, we assumed that nucleophilic attacks occurred selectively at the *Re* face of ketone **8**, giving the “*all-cis”* 3, 4, 5-trihydroxypiperidines (Scheme 5). A careful analysis of the ^1^H NMR spectra of derivatives **26**–**31** and the products of their transformations supports this structural assignment (see below). 

**Scheme 6:**
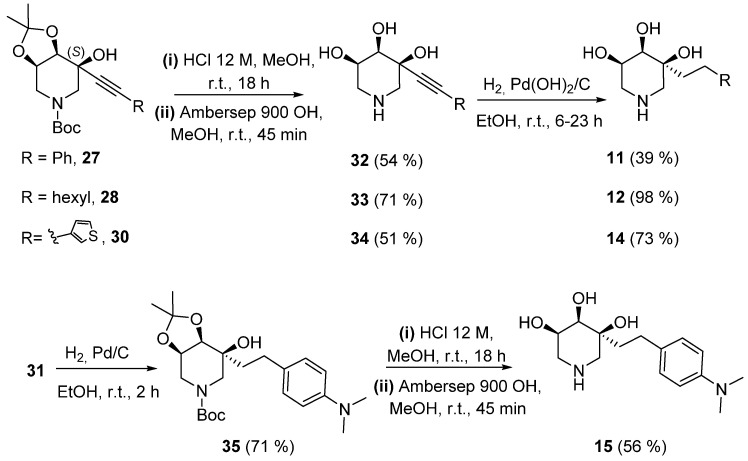
Synthesis of trihydroxypiperidines **11**, **12**, **14,** and **15**.

Compounds **27**, **28,** and **30** were subjected to acidic treatment for the concomitant removal of acetonide and Boc-protecting groups, leading to the “*all-cis*” trihydroxypiperidines **32**, **33,** and **34** with good yields (51–71%) after treatment with the strongly basic resin Ambersep 900 OH. The triple bond was subsequently reduced by catalytic hydrogenation in the presence of Pd(OH)_2_/C in EtOH to give azasugars **11**, **12,** and **14** (39–98%) (Scheme 6). 

Due to the presence of the additional acid-labile acetal moiety, adduct **29** was expected to give complications arising from possible interactions of the free amine with the aldehyde and was not subjected to acid-induced deprotection. Unsuccessful deprotection of **31** with different acids (CF_3_COOH, CH_3_COOH, and HCl) was ascribed to the higher reactivity of its triple bond. Therefore, hydrogenation of the alkyne under neutral conditions (H_2_, Pd/C in EtOH) was first carried out to give piperidine **35**, which was subsequently deprotected with aqueous HCl in MeOH. Final treatment with the strongly basic resin Ambersep 900 OH gave trihydroxypiperidine **15** with a 56% yield (Scheme 6).

### 2.2. Configuration Assignment

Relevant chemical shifts and coupling constants are reported in Table 2 and Table 3 for H-4, H-5, and H-6 in ^1^H-NMR spectra in selected compounds of the two series of protected (in CDCl_3_) and deprotected (in CD_3_OD) trihydroxypiperidines, respectively. These values show regularities (the same applies to H-2 signals), which allowed us to ascribe the same configuration at C-3 for all compounds (see the Supplementary Materials for the full Table). Moreover, the shape of the signals and their coupling constants, where detectable, are consistent with the (*S*) absolute configuration tentatively assigned (see above) on the basis of mechanistic considerations. Indeed, the signal of H-5 appears as a broad singlet (or as a narrow multiplet), which is in agreement with its equatorial position in a preferred chair conformation, which places the R substituent equatorially, i.e., in the (3*S*) configuration (_6_C^3^ alcohol in Scheme 5). The lack of large *ax-ax* coupling constants is confirmed by signals of H-6. For example, in piperidine **13** (R = ethyl), the two hydrogens at C-6 display vicinal coupling constants J = 3.4 and J = 2.4 Hz, typical for *ax-eq* and *eq-eq* relationships, respectively. The same applies to the other derivatives when the signals are well resolved, as in compounds **11**, **12**, **14,** and **15**. 

The observed upfield shift (0.3–0.5 ppm) of H-4 within the two series of compounds on turning from the alkynyl to the saturated substituents (see for instance **32** vs. **11**), consistent with H-4 falling in the deshielding cone of the triple bond in the former derivatives when in a *cis* relationship, further supports this assignment.

### 2.3. Biological Screening

Compounds **9**–**15**, **21**, **32**, and **33** were first evaluated as human lysosomal β-Gal inhibitors at 1 mM in human leukocyte homogenates and the results are shown in Table 4 and compared to previously published data. Unfortunately, none of the tested compounds strongly inhibited β-Gal (only a moderate 22% inhibition was found for the *“all-cis”* ether **9**). These data demonstrate that both the alkylation of the hydroxy or the exocyclic amine group and the introduction of a substituent at C-3 of the trihydroxypiperidine skeleton dramatically affect β-Gal inhibition. 

However, the screening on a panel of 12 commercial glycosidases (see the Supplementary Materials), showed that only trihydroxypiperidine **10** (bearing a dodecyl chain connected at C-3 through a nitrogen atom) was able to inhibit β-glucosidase from almonds. In particular, compound **10** showed an IC_50_ = 85 µM towards this enzyme, which prompted us to evaluate compounds **9**–**15**, **21**, **32,** and **33** also on human lysosomal β-glucosidase (GCase) (Table 4). Point mutations in the gene encoding this enzyme cause Gaucher disease, the most common autosomal recessive LSD [42,43]. 

In keeping with the results on commercially available β-glucosidase, the best GCase inhibitor was compound **10**, bearing a dodecylamino substituent at C-3 (IC_50_ = 6.4 µM, Table 4, entry 2). Regarding the newly synthesized ethers, the *“all-cis”* **9** showed an inhibitory activity towards GCase one order of magnitude greater than its epimer **21** (IC_50_ = 12 µM vs. IC_50_ = 130 µM, Table 4, entries 1 vs. 8). Among the compounds derived from organometal addition reactions, the lowest inhibitory activities were observed for thienyl and dimethylaminophenyl-containing derivatives **14** and **15** (Table 4, entries 6 and 7). Moreover, GCase was poorly inhibited also by compound **13**, bearing a short ethyl chain (Table 4, entry 5), and by trihydroxypiperidines **11** and **32,** bearing the phenylethyl or the phenylethynyl substituent, respectively (Table 4, entries 3 and 9).

Conversely compound **33**, bearing the triple bond and a longer aliphatic substituent, showed 65% inhibition, which increased considerably after hydrogenation to **12** (Table 4, entry 10 vs. 4). This latter compound showed a moderate IC_50_ = 60 µM, which is close to that observed for trihydroxypiperidines bearing an octyl chain at C-2, previously synthesized in our group (**37** and **39**, Table 4, entries 12 and 14) [23,24]. 

These data overall show that GCase inhibition higher than 90% is guaranteed by the presence of a long alkyl chain (8, 9, or 12 carbon atoms, compounds **9**, **10**, **12,** and **21**). This parallels previous studies, which showed that alkylated imino- and azasugars are strong GCase inhibitors due to favorable interaction of the alkyl chain with the hydrophobic domain of the enzyme [24,44,45]. However, in terms of IC_50_ values, a remarkable difference was found between compounds **9** and **21**, in agreement with previous observations by Compain and coworkers on differently configured ethers derived from 1,5-dideoxy and 1-5-imino-d-xylitol (DIX) [46]. Moreover, good GCase inhibitors can be identified among piperidines with only two free hydroxy groups (e.g., **9**, **10,** and **21**), as previously observed with different azasugars [46].

We then assayed the strongest GCase inhibitors **9**, **10,** and **12** (IC_50_ lower than 100 μM) in human fibroblasts derived from Gaucher patients bearing the N370S mutation. None of the compounds gave enzyme rescue when tested at six different concentrations (10, 100, 1, 10, 50, and 100 µM) (see the Supplementary Materials). In particular, compound **10** showed remarkable toxicity at the highest concentrations (50 and 100 µM). Notably, the stronger inhibitory activity of **10** with respect to **36** (see Table 4) does not correspond to a higher chaperoning activity (indeed, compound **36** was able to rescue GCase activity of 1.5-fold at 100 µM) [22].

The same behavior is evident by comparing the C-2 alkylated trihydroxypiperidines **38** and **37**: The best inhibitor was the dodecyl alkylated (compound **38**) but the best chaperone was the octyl derivative **37** [24]. The inefficacy of dodecyl alkylated compounds **10** and **38** as PCs can be ascribed to the cytotoxicity imparted by the 12-carbon atom alkyl chains. These data are also consistent with other reports on amphiphilic *N*-alkylated iminosugars, which suggest that cytotoxicity is strongly chain-length dependent and that potent inhibitors with chains longer than C_8_ can be toxic when assayed in cell lines [47,48].

## 3. Materials and Methods 

### 3.1. General Experimental Procedures for the Syntheses

Commercial reagents were used as received. All reactions were carried out under magnetic stirring and monitored by TLC on 0.25 mm silica gel plates (Merck F254). Column chromatographies were carried out on silica gel 60 (32–63 μm) or on silica gel (230–400 mesh, Merck, Kenilworth, NJ, USA). Yields refer to spectroscopically and analytically pure compounds unless otherwise stated. ^1^H-NMR spectra were recorded on a Varian Gemini 200 MHz, a Varian Mercury 400 MHz, or on a Varian INOVA 400 MHz instrument at 25 °C (Agilent Technologies, Santa Clara, CA, USA). ^13^C-NMR spectra were recorded on a Varian Gemini 200 MHz or on a Varian Mercury 400 MHz instrument (Agilent Technologies, Santa Clara, CA, USA). Chemical shifts are reported relative to CDCl_3_ (^13^C: δ = 77.0 ppm) or to CD_3_OD (^13^C: δ = 49.0 ppm). Integrals are in accordance with assignments, coupling constants are given in Hz. For detailed peak assignments 2D spectra were measured (COSY, HSQC, NOESY, and NOE as necessary). IR spectra were recorded with a IRAffinity-1S SHIMADZU system spectrophotometer (Shimadzu Italia S.r.l., Milan, Italy) ESI-MS spectra were recorded with a Thermo Scientific™ LCQ fleet ion trap mass spectrometer (Thermo Fisher Scientific,Waltham, MA, USA). Elemental analyses were performed with a Thermo Finnigan FLASH EA 1112 CHN/S analyzer (Perkin-Elmer, Waltham, MA, USA). Optical rotation measurements were performed on a JASCO DIP-370 polarimeter (JASCO, Easton, MD, USA).

#### 3.1.1. Synthesis of (3*R*, 4*S*, 5*S*)-3, 4-*O*-(1-Methylethylidene)-5-Nonyloxy-*N*-Boc-Piperidine (**19**)

NaH (7 mg, 0.3 mmol, 60% on mineral oil) was added to a solution of **18** [25] (22 mg, 0.08 mmol) in dry DMF (1.2 mL) at 0 °C. The mixture was stirred at room temperature for 30 min, then 1-bromononane (54 µL, 0.28 mmol) was added, and the reaction mixture was stirred at room temperature for 72 h, until the disappearance of the starting material was observed via TLC (CH_2_Cl_2_/MeOH/NH_4_OH (6%) 10:1:0.1). Then, water was slowly added, and the reaction mixture was extracted with AcOEt (3 × 3 mL). The combined organic layer was washed with saturated NaHCO_3_ and brine and concentrated after drying with Na_2_SO_4_. The crude residue was purified by flash column chromatography on silica gel (hexane/AcOEt 8:1) to give 17 mg of **19** (R_f_ = 0.3, hexane/AcOEt 8:1, 0.04 mmol, 53%) as a colorless oil.

**19**: $[\alpha {]}_{D}^{21}$ = +17.7 (c = 0.75, CHCl_3_). ^1^H-NMR (400 MHz, CDCl_3_) *δ* ppm = 4.51–4.44 (m, 1H, H-3), 4.28 (br s, 1H, H-4), 3.85–3.07 (m, 7H, H-2, H-6, H-5, H-1′), 1.66–1.56 (m, 2H, H-2′), 1.49 (s, 3H, Me), 1.45 (s, 9H, t-Bu), 1.36 (s, 3H, Me), 1.34–1.20 (m, 12H, H-3′, H’4′, H-5′, H-6′, H-7′, H-8′), 0.90–0.85 (m, 3H, H-9′). ^13^C-NMR (50 MHz, CDCl_3_) *δ* ppm = 155.3 (s, 1C, NCOO), 109.8 (s, 1C, OC(CH_3_)_2_), 80.0 (s, 1C, OC(CH_3_)_3_), 73.4 (d, 1C, C-5), 72.7 (d, 2C, C-3, C-4), 70.4 (t, 1C, C-1′), 44.0, 42.8, 41.8, 41.5 (t, 2C, C-2, C-6), 32.0, 29.9, 29.7, 29.6, 29.4, 27.2, 26.1, 25.3, 22.8 (t, 7C, C-2′, C-3′, C-4′, C-5′, C-6′, C-7′, C-8′ and q, 2C, OC(CH_3_)_2_), 28.6 (q, 3C, OC(CH_3_)_3_), 14.2 (q, 1C, C-9′). IR (CDCl_3_): ν = 2959, 2928, 2857, 2249, 1686, 1375, 1261, 1165, 1101, 1172, 1008 cm^−1^. C_22_H_41_NO_5_ (399.56): calcd. C, 66.13; H, 10.34; N, 3.51; found C, 66.23; H, 10.32; N, 3.45. MS-ESI (*m*/*z*, %) = 422.16 (100) [M + Na]^+^, 820.89 (17) [2M + Na]^+^.

#### 3.1.2. Synthesis of (3*S*, 4*R*, 5*R*)-4, 5-Dihydroxy-3-(Nonyloxy) Piperidine (**9**)

A solution of **19** (15 mg, 0.04 mmol) in MeOH (3 mL) was left stirring with 12 M HCl (60 μL) at room temperature for 18 h. The crude mixture was concentrated to yield **9** as the hydrochloride salt. The corresponding free amine was obtained by dissolving the residue in MeOH (4 mL), then the strongly basic resin Ambersep 900 OH was added, and the mixture was stirred for 45 min. The resin was removed by filtration and the crude product was purified on silica gel by flash column chromatography (DCM/MeOH/NH_4_OH (6%) 10:1:0.1) to afford 7 mg of **9** (R_f_ = 0.2, DCM/MeOH/NH_4_OH (6%) 10:1:0.1, 0.03 mmol, 70%) as a pale-yellow oil.

**9**: $[\alpha {]}_{D}^{22}$ = −4.6 (c = 0.54, MeOH). ^1^H-NMR (400 MHz, CD_3_OD) *δ* ppm: 4.01 (br s, 1H, H-4), 3.60–3.45 (m, 3H, H-5, H-1′), 3.38–3.32 (m, 1H, H-3), 2.84–2.66 (m, 4H, H-2, H-6), 1.63–1.54 (m, 2H, H-2′), 1.40–1.25 (m, 12H, H-3′, H’4′, H-5′, H-6′, H-7′, H-8′), 0.90 (t, *J* = 6.9 Hz, 3H, H-9′). ^13^C-NMR (100 MHz, CD_3_OD) *δ* ppm: 78.5 (d, 1C, C-3), 70.5 (d, 1C, C-5), 70.4 (t, 1C, C-1′), 70.3 (d, 1C, C-4), 47.5, 44.7 (t, 2C, C-2, C-6), 33.1, 31.0, 30.8, 30.7, 30.6, 30.4, 27.2, 23.7 (t, 7C, C-2′, C-3′, C-4′, C-5′, C-6′, C-7′, C-8′), 14.4 (q, 1C, C-9′). C_14_H_29_NO_3_ (259.38): calcd. C, 64.83; H, 11.27; N, 5.40; found C, 64.85; H, 11.13; N, 5.60. MS-ESI (*m*/*z*, %) = 260.12 (100) [M + H]^+^, 282.29 (58) [M + Na]^+^, 540.91 (25) [2M + Na]^+^.

#### 3.1.3. Synthesis of (3*R*, 4*S*, 5*R*)-3, 4-*O*-(1-Methylethylidene)-5-Nonyloxy-*N*-Boc-Piperidine (**20**)

NaH (11 mg, 0.46 mmol, 60% on mineral oil) was added to a solution of **17** [25] (57 mg, 0.21 mmol) in dry DMF (3 mL) at 0 °C. The mixture was stirred at room temperature for 30 min, then 1-bromononane (140 µL, 0.73 mmol) was added, and the reaction mixture was stirred at room temperature for 40 h, until the disappearance of the starting material was observed via TLC (CH_2_Cl_2_/MeOH/NH_4_OH (6%) 10:1:0.1). Then, water was slowly added and the reaction mixture was extracted with AcOEt (3 × 5 mL). The combined organic layer was washed with saturated NaHCO_3_ and brine and concentrated after drying with Na_2_SO_4_. The crude residue was purified by flash column chromatography on silica gel (hexane/AcOEt 10:1) to give 45 mg of **20** (R_f_ = 0.4, hexane/AcOEt 7:1, 0.11 mmol, 54%) as a colorless oil.

**20**: $[\alpha {]}_{D}^{27}$= +1.8 (c = 0.96, CHCl_3_). ^1^H-NMR (400 MHz, CDCl_3_) *δ* ppm: 4.31–4.26 (m, 1H, H-3), 4.13 (br s, 1H, H-4), 3.92–3.82 (m, 1H, Ha-2), 3.58–3.28 (m, 6H, Hb-2, H-5, H-6, H-1′), 1.59–1.48 (m, 2H, H-2′), 1.45 (s, 12H, Me, t-Bu), 1.33 (s, 3H, Me), 1.32–1.17 (m, 12H, H-3′, H’4′, H-5′, H-6′, H-7′, H-8′), 0.87 (t, *J* = 6.2 Hz, 3H, H-9′). ^13^C-NMR (50 MHz, CDCl_3_) *δ* ppm: 155.8 (s, 1C, NCOO), 109.1 (s, 1C, OC(CH_3_)_2_), 79.8 (s, 1C, OC(CH_3_)_3_), 74.8 (d, 1C, C-5), 74.5 (d, 1C, C-4), 72.6 (d, 1C, C-3), 69.6 (t, 1C, C-1′), 43.0, 42.0 (t, 1C, C-2), 42.0–40.8 (t, 1C, C-6), 32.0, 30.0, 29.7, 29.6, 29.4, 27.3, 22.8 (t, 7C, C-2′, C-3′, C-4′, C-5′, C-6′, C-7′, C-8′), 28.6 (q, 3C, OC(CH_3_)_3_), 26.3, 25.0 (q, 2C, OC(CH_3_)_2_), 14.2 (q, 1C, C-9′). IR (CDCl_3_): ν = 2930, 2859, 1686, 1416, 1375, 1260, 1165, 1101, 1063, 1016 cm^−1^. C_22_H_41_NO_5_ (399.56): calcd. C, 66.13; H, 10.34; N, 3.51; found C, 66.15; H, 10.40; N, 3.60. MS-ESI (*m*/*z*, %) = 422.17 (100) [M + Na]^+^, 821.05 (96) [2M + Na]^+^.

#### 3.1.4. Synthesis of (3*R*, 4*R*, 5*R*)-4, 5-Dihydroxy-3-(Nonyloxy) Piperidine (**21**)

A solution of **20** (54 mg, 0.14 mmol) in MeOH (6 mL) was left stirring with 12 M HCl (150 μL) at room temperature for 18 h. The crude mixture was concentrated to yield 21 as hydrochloride salt. The corresponding free amine was obtained by dissolving the residue in MeOH (5 mL), then the strongly basic resin Ambersep 900 OH was added, and the mixture was stirred for 45 min. The resin was removed by filtration to give 36 mg of **21** (0.14 mmol, 100% yield) as a pale-yellow oil. 

**21**: $[\alpha {]}_{D}^{25}$ = −39.6 (c = 0.48, MeOH). ^1^H-NMR (400 MHz, CD_3_OD) *δ* ppm: 3.84–3.77 (m, 1H, H-5), 3.67–3.60 (m, 1H, H-4), 3.60–3.52 (m, 2H, H-1′) 3.46–3.39 (m, 1H, H-3), 3.02 (d, *J* = 13.4 Hz, 1H, Ha-2), 2.82 (dd, *J* = 6.1, 13.2 Hz, 1H, Ha-6), 2.65 (d, *J* = 13.3 Hz, 1H, Hb-6), 2.48–2.38 (m, 1H, Hb-2), 1.62–1.51 (m, 2H, H-2′), 1.41–1.20 (m, 12H, H-3′, H’4′, H-5′, H-6′, H-7′, H-8′), 0.90 (t, *J* = 5.9 Hz, 3H, H-9′). ^13^C-NMR (50 MHz, CD_3_OD) *δ* ppm: 78.6 (d, 1C, C-3), 73.2 (d, 1C, C-4), 71.2 (t, 1C, C-1′), 69.5 (d, 1C, C-5), 49.3 (t, 1C, C-6), 47.1 (t, 1C, C-2), 33.0, 31.2, 30.7, 30.6, 30.4, 27.2, 23.7 (t, 7C, C-2′, C-3′, C-4′, C-5′, C-6′, C-7′, C-8′), 14.4 (q, 1C, C-9′). C_14_H_29_NO_3_ (259.38): calcd. C, 64.83; H, 11.27; N, 5.40; found C, 64.50; H, 11.38; N, 5.30. MS-ESI (*m*/*z*, %) = 260.18 (100) [M + H]^+^.

#### 3.1.5. Synthesis of (3*R*, 4*S*, 5*S*)-5-Dodecylamino-3, 4-*O*-(1-Methylethylidene)-*N*-Boc-Piperidine (**22**)

Ketone **8** [25] (63 mg, 0.23 mmol) and dodecylamine (65 mg, 0.35 mmol) were dissolved in MeOH (3 mL), and molecular sieves (3 Å pellets; 25 mg) were added. The reaction mixture was stirred at room temperature for 1 h and then Pd(OH)_2_/C (30 mg) was added. The mixture was further stirred at room temperature under hydrogen atmosphere for 51 h. The catalyst and the molecular sieves were removed by filtration, the obtained compound was washed several times with MeOH, and the solvent was evaporated under vacuum. The crude residue was purified by flash column chromatography on silica gel (gradient eluent from hexane/AcOEt 5:1 to 2:1) to afford 56 mg of **22** (R_f_ = 0.4, hexane/AcOEt 2:1, 0.13 mmol, 55%) as a colorless oil.

**22**: $[\alpha {]}_{D}^{27}$= +1.9 (c = 0.90, CHCl_3_). ^1^H-NMR (400 MHz, CDCl_3_) *δ* ppm: 4.41 (br s, 1H, H-4), 4.29 (br s, 1H, H-3), 3.85–3.40 (m, 2H), 3.40–3.22 (m, 1H), 2.99–2.84 (m, 1H), 2.83–2.75 (m, 1H, H-5), 2.74–2.54 (m, 2H, H-1′), 1.44 (s, 12H, Me, t-Bu), 1.33 (s, 3H, Me), 1.31–1.17 (m, 20H, H-2′, H-3′, H-5′, H-6′, H-7′, H-8′, H-9′, H-10′, H-11′), 0.86 (t, *J* = 6.6 Hz, 3H, H-12′). ^13^C-NMR (100 MHz, CDCl_3_) *δ* ppm: 155.4 (s, 1C, NCOO), 108.9 (s, 1C, OC(CH_3_)_2_), 79.8 (s, 1C, OC(CH_3_)_3_), 72.5 (d, 2C, C-3, C-4), 53.7 (d, 1C, C-5), 47.2 (t, 1C, C-1′), 43.6, 43.3, 42.4, 42.3 (t, 2C, C-2, C-6), 32.0, 30.6, 29.8, 29.7, 29.6, 29.5, 27.4, 27.1, 25.1, 22.6 (t, 10C, C-2′, C-3′, C-4′, C-5′, C-6′, C-7′, C-8′, C-9′, C-10′, C-11′ and q, 2C, OC(CH_3_)_2_, 28.6 (q, 3C, OC(CH_3_)_3_), 14.2 (q, 1C, C-12′). IR (CDCl_3_): ν = 3300, 2958, 2927, 2854, 1686, 1414, 1373, 1260, 1165, 1098, 1007 cm^−1^. C_25_H_48_N_2_O_4_ (440.66): calcd. C, 68.14; H, 10.98; N, 6.36; found C, 68.30; H, 10.78; N, 6.33. MS-ESI (*m*/*z*, %) = 441.28 (100) [M + H]^+^, 463.23 (91) [M + Na]^+^, 903.11 (51) [2M + Na]^+^.

#### 3.1.6. Synthesis of (3*R*, 4*S*, 5*S*)-3, 4-Dihydroxy-5-(Dodecylamino) Piperidine (**10**)

A solution of **22** (45 mg, 0.10 mmol) in MeOH (6 mL) was left stirring with 12 M HCl (150 μL) at room temperature for 18 h. The crude mixture was concentrated to yield **10** as hydrochloride salt. The corresponding free amine was obtained by dissolving the residue in MeOH (5 mL), then the strongly basic resin Ambersep 900 OH was added, and the mixture was stirred for 45 min. The resin was removed by filtration to give 25 mg of **10** (0.08 mmol, 83%) as a white solid. 

**10**: M.p. = 92–94 °C. $[\alpha {]}_{D}^{24}$ = −5.2 (c = 0.85, MeOH). ^1^H-NMR (400 MHz, CD_3_OD) *δ* ppm: 4.00 (br s, 1H, H-4), 3.58–3.51 (m, 1H, H-3), 2.80–2.51 (m, 7H, H-2, H-6, H-5, H-1′), 1.55–1.47 (m, 2H, H-2′), 1.37–1.26 (m, 18H, H-3′, H-4′, H-5′, H-6′, H-7′, H-8′, H-9′, H-10′, H-11′), 0.90 (t, *J* = 6.0 Hz, 3H, H-12′). ^13^C-NMR (50 MHz, CD_3_OD) *δ* ppm: 70.9 (d, 1C, C-3), 69.8 (d, 1C, C-4), 58.7 (d, 1C, C-5), 47.4, 45.4 (t, 3C, C-1′, C-2, C-6), 33.1, 30.9, 30.7, 30.5, 28.5, 23.7 (t, 10C, C-2′, C-3′, C-4′, C-5′, C-6′, C-7′, C-8′, C-9′, C-10′, C-11′) 14.4 (q, 1C, C-12′). C_17_H_36_N_2_O_2_ (300.48): calcd. C, 67.95; H, 12.08; N, 9.32; found C, 67.96; H, 12.03; N, 9.53. MS-ESI (*m*/*z*, %) = 301.28 (100) [M + H]^+^.

#### 3.1.7. Synthesis of (3*S*, 4*R*, 5*R*)-3-Hydroxy-4, 5-*O*-(1-Methylethylidene)-3-Vinyl-*N*-Boc-Piperidine (**26**)

Vinyl magnesium bromide (288 µL, 0.29 mmol) was added to a dry THF solution (1 mL) of ketone **8** (52 mg, 0.19 mmol), dropwise at 0 °C under nitrogen atmosphere. The solution was stirred at 0 °C for 5 h when the disappearance of 8 was attested by a TLC control (hexane/AcOEt 2:1). A saturated aqueous NH_4_Cl solution was added at 0 °C, and the mixture was stirred for 10 min. The reaction mixture was extracted with AcOEt (3 × 3 mL). The combined organic layer was washed with water, saturated NaHCO_3_, and brine and concentrated after drying with Na_2_SO_4_. The crude residue was purified by flash column chromatography on silica gel (gradient eluent from hexane/AcOEt 5:1 to 2:1) to give 29 mg of **26** (R_f_ = 0.2, hexane/AcOEt 5:1, 0.10 mmol, 51%) as a pale-yellow oil. 

**26**: $[\alpha {]}_{D}^{25}$ = −22.9 (c = 1.00, CHCl_3_). ^1^H-NMR (400 MHz, CDCl_3_) *δ* ppm: 5.85 (dd, *J* = 10.8, 17.2 Hz, 1H, H-1′), 5.49 (dd, *J* = 0.8, 17.2 Hz, 1H, H-2′_trans_), 5.28 (d, *J* = 10.8 Hz, 1H, H-2′_cis_), 4.33 (br s, 1H, H-5), 4.07 (d, *J* = 6.8 Hz, 1H, H-4), 3.95–3.69 (m, 1H, Ha-6), 3.53–3.32 (m, 1H, Hb-6), 3.46 (d, *J* = 13.0 Hz, 1H, Ha-2), 3.25 (d, *J* = 13.2 Hz, 1H, Hb-2), 2.67 (br s, 1H, OH), 1.52 (s, 3H, Me), 1.44 (s, 9H, t-Bu), 1.37 (s, 3H, Me). ^13^C-NMR (100 MHz, CDCl_3_) *δ* ppm: 155.1 (s, 1C, NCOO)_,_ 139.3 (d, 1C, C-1′), 116.8 (t, 1C, C-2′), 109.5 (s, 1C, OC(CH_3_)_2_), 80.2 (s, 1C, OC(CH_3_)_3_)_,_ 77.2 (d, 1C, C-4), 71.9 (d, 1C, C-5), 71.3 (s, 1C, C-3), 48.4, 47.5 (t, 1C, C-2), 43.4, 42.5 (t, 1C, C-6), 28.5 (q, 3C, OC(CH_3_)_3_), 27.0 (q, 1C, OC(CH_3_)_2_), 25.0 (q, 1C, OC(CH_3_)_2_). IR (CDCl_3_) ν = 3555, 2984, 2932, 2251, 1688, 1410, 1385, 1373, 1252, 1213, 1163, 1070 cm^−1^. C_15_H_25_NO_5_ (299.36): calcd. C, 60.18; H, 8.42; N, 4.68; found C, 60.37; H, 8.60; N, 4.67. MS-ESI (*m*/*z*, %) = 322.03 (100) [M + Na]^+^, 620.85 (40) [2M + Na]^+^, 338.03 (12) [M + K]^+^.

#### 3.1.8. Synthesis of (3*S*, 4*R*, 5*R*)-3-Ethyl-3, 4, 5-Trihydroxypiperidine (**13**)

Compound **26** (26 mg, 0.09 mmol) was dissolved in EtOH (5 mL) and HCl 12 M (150 µL) and Pd/C (13 mg) were added. The reaction mixture was stirred at room temperature under hydrogen atmosphere for 24 h. The catalyst was removed by filtration through Celite, and the filtrate was concentrated under vacuum to give the hydrochloride salt of **13**. The corresponding free amine was obtained by dissolving the residue in MeOH, then the strongly basic resin Ambersep 900 OH was added, and the mixture was stirred for 45 min. The resin was removed by filtration and the crude product was purified on silica gel by flash column chromatography (gradient eluent from DCM/MeOH/NH_4_OH (6%) 10:1:0.1 to 1:1:0.1) to give 8 mg of **13** (R_f_ = 0.2, DCM/MeOH/NH_4_OH (6%) 10:1:0.1, 0.05 mmol, 52%) as a colorless oil. 

**13**: $[\alpha {]}_{D}^{24}$ = −5.7 (c = 0.75, CD_3_OD). ^1^H-NMR (400 MHz, CD_3_OD) *δ* ppm: 3.81 (br s, 1H, H-5), 3.47 (d, *J* = 3.2 Hz, 1H, H-4), 2.96 (dd, *J* = 3.4, 13.6 Hz, 1H, Ha-6), 2.79 (br d, *J* = 13.6 Hz, 1H, Ha-2), 2.72 (dd, *J* = 2.4, 13.7 Hz, 1H, Hb-6), 2.55 (d, *J* = 13.6 Hz, 1H, Hb-2), 1.68–1.55 (m, 2H, H-1′), 0.90 (t, *J* = 7.6 Hz, 3H, H-2′). ^13^C-NMR (50 MHz, CD_3_OD) *δ* ppm: 75.1 (s, 1C, C-3), 72.1 (d, 1C, C-4), 70.7 (d, 1C, C-5), 53.0 (t, 1C, C-2), 50.3 (t, 1C, C-6), 29.6 (t, 1C, C-1′), 7.6 (q, 1C, C-2′). C_7_H_15_NO_3_ (161.20): calcd. C, 52.16; H, 9.38; N, 8.69; found C, 52.06; H, 9.46; N, 8.71. MS-ESI (*m*/*z*, %) = 184.15 (100) [M + Na]^+^.

#### 3.1.9. General Procedure for the Addition of Lithium Acetylides to Ketone **8**

To a dry THF solution (0.24 M) of alkyne (2 eq.), *n*-BuLi (1.5 eq.) was added dropwise over 5 min at –78 °C under nitrogen atmosphere. The solution was allowed to warm to 0 °C over 1 h and held at 0 °C for an additional 30 min. The solution was then recooled to –78 °C and ketone **8** (1 eq.) was added in one portion. The solution was allowed to warm to room temperature and stirred at room temperature until the disappearance of ketone **8** was attested by a TLC control (EtP/AcOEt 2:1). A saturated aqueous solution of NH_4_Cl was added and the reaction mixture was extracted with AcOEt. The combined organic layer was washed with water, saturated NaHCO_3_, and brine and concentrated after drying with Na_2_SO_4_. The crude compound was purified by flash column chromatography.

#### 3.1.10. Synthesis of (3*S*, 4*R*, 5*R*)-3-Hydroxy-4, 5-*O*-(1-Methylethylidene)-3-(Phenylethynyl)-*N*-Boc-Piperidine (**27**)

Application of the general procedure to ketone **8** (60 mg, 0.24 mmol) with phenylacetylene (54 µL, 0.49 mmol) and *n*-BuLi (150 µL, 0.37 mmol, 2.5 M) gave, after purification by flash column chromatography on silica gel (hexane/AcOEt 4:1), 69 mg of **27** (R_f_ = 0.2, 0.18 mmol, 77%) as a pale-yellow oil.

**27**: $[\alpha {]}_{D}^{27}$ = +9.4 (c = 1.05, CHCl_3_). ^1^H-NMR (400 MHz, CDCl_3_) *δ* ppm: 7.40–7.34 (m, 2H, Ar), 7.30–7.21 (m, 3H, Ar), 4.52–4.40 (m, 1H, H-5), 4.36 (d, *J* = 6.8 Hz, 1H, H-4), 3.92–3.70 (m, 2H, Ha-2, Ha-6), 3.67–3.55 (m, 1H, Hb-6), 3.37–3.28 (m, 1H, Hb-2), 3.09–2.97 (m, 1H, OH), 1.50 (s, 3H, Me), 1.40 (s, 9H, t-Bu), 1.36 (s, 3H, Me). ^13^C-NMR (50 MHz, CDCl_3_) *δ* ppm: 155.2 (s, 1C, NCOO)_,_ 132.0, 129.0, 128.4 (d, 5C, Ar), 121.9 (s, 1C, Ar), 109.9 (s, 1C, OC(CH_3_)_2_), 88.1, 86.4 (s, 2C, C-1′, C-2′), 80.2 (s, 1C, OC(CH_3_)_3_), 77.1 (d, 1C, C-4), 72.5 (d, 1C, C-5), 67.4 (s, 1C, C-3), 48.3, 47.5 (t, 1C, C-6), 42.7, 41.9 (t, 1C, C-2), 28.5 (q, 3C, OC(CH_3_)_3_), 27.0 (q, 1C, OC(CH_3_)_2_), 25.0 (q, 1C, OC(CH_3_)_2_). IR (CDCl_3_) ν = 3541, 2982, 2934, 2251, 1688, 1600, 1260, 1215, 1163 cm^−1^. C_21_H_27_NO_5_ (373.44): calcd. C, 67.54; H, 7.29; N, 3.75; found C, 67.40; H, 7.33; N, 3.76. MS-ESI (*m*/*z*, %) = 769.01 (100) [2M + Na]^+^, 396.11 (21) [M + Na]^+^.

#### 3.1.11. Synthesis of (3*S*, 4*R*, 5*R*)-3-Hydroxy-4, 5-*O*-(1-Methylethylidene)-3-(oct-1-yn-1-yl)-*N*-Boc-Piperidine (**28**)

Application of the general procedure to ketone **8** (120 mg, 0.45 mmol) with 1-octyne (131 µL, 0.89 mmol) and *n*-BuLi (267 µL, 0.67 mmol, 2.5 M) gave, after purification by flash column chromatography on silica gel (hexane/AcOEt 4:1), 134 mg of **28** (R_f_ = 0.2, 0.35 mmol, 78%) as a colorless oil.

**28**: $[\alpha {]}_{D}^{27}$ = +5.5 (c = 1.03, CHCl_3_). ^1^H-NMR (400 MHz, CDCl_3_) *δ* ppm: 4.47–4.34 (m, 1H, H-5), 4.24 (d, *J* = 6.9 Hz, 1H, H-4), 3.74–3.54 (m, 3H, Ha-2, H-6), 3.22 (d, *J* = 12.3 Hz, 1H, Hb-2), 2.87 (br s, 1H, OH), 2.19–2.12 (m, 2H, H-3′), 1.48 (s, 3H, Me), 1.43 (s, 9H, t-Bu), 1.35 (s, 3H, Me), 1.34–1.15 (m, 8H, H-4′, H-5′, H-6′, H-7′), 0.91–0.79 (m, 3H, H-8′). ^13^C-NMR (100 MHz, CDCl_3_) *δ* ppm: 155.2 (s, 1C, NCOO)_,_ 109.6 (s, 1C, OC(CH_3_)_2_), 87.5, 79.4 (s, 2C, C-1′, C-2′), 80.0 (s, 1C, OC(CH_3_)_3_), 77.2 (d, 1C, C-4), 72.4 (d, 1C, C-5), 66.8 (s, 1C, C-3), 48.3, 47.5 (t, 1C, C-2), 42.5, 41.6 (t, 1C, C-6), 31.3, 28.6, 28.4, 22.6 (t, 4C, C-4′, C-5′, C-6′-C-7′) e 26.9 (q, 1C, OC(CH_3_)_2_), 24.9 (q, 1C, OC(CH_3_)_2_), 28.5 (q, 3C, OC(CH_3_)_3_)_,_ 18.8 (t, 1C, C-3′), 14.1 (q, 1C, C-8′). IR (CDCl_3_) ν = 3543, 2961, 2932, 2862, 2249, 1730, 1458, 1412, 1375, 1215, 1167, 1142, 1045 cm^−1^. C_21_H_35_NO_5_ (381.51): calcd. C, 66.11; H, 9.25; N, 3.67; found C, 66.34; H, 9.38; N, 3.73. MS-ESI (*m*/*z*, %) = 785.02 (100) [2M + Na]^+^, 404.12 (32) [M + Na]^+^.

#### 3.1.12. Synthesis of (3*S*, 4*R*, 5*R*)-3-Hydroxy-4, 5-*O*-(1-Methylethylidene)-3-(3, 3-Diethoxyprop-1-yn-1-yl)-*N*-Boc-Piperidine (**29**)

Application of the general procedure to ketone **8** (60 mg, 0.22 mmol) with 3, 3-diethoxyprop-1-yne (64 µL, 0.44 mmol) and *n*-BuLi (208 µL, 0.33 mmol, 1.6 M) gave, after purification by flash column chromatography on silica gel (EtP/AcOEt 2:1), 57 mg of **29** (R_f_ = 0.3, 0.14 mmol, 65%) as a pale-yellow oil.

**29**: $[\alpha {]}_{D}^{25}$ = +19.8 (c = 1.00, CHCl_3_). ^1^H-NMR (400 MHz, CDCl_3_) *δ* ppm: 5.25 (s, 1H, H-3′), 4.47–4.36 (m, 1H, H-5), 4.30 (d, *J* = 6.8 Hz, 1H, H-4), 3.78–3.49 (m, 7H, Ha-2, H-6, H-4′, H-6′), 3.32 (d, *J* = 12.6 Hz, 1H, Hb-2), 3.05–2.96 (m, 1H, OH), 1.50 (s, 3H, Me), 1.44 (s, 9H, t-Bu), 1.36 (s, 3H, Me), 1.19 (t, *J* = 7.1 Hz, 6H, H-5′, H-7′). ^13^C-NMR (50 MHz, CDCl_3_) *δ* ppm: 155.4 (s, 1C, NCOO)_,_ 110.2 (s, 1C, OC(CH_3_)_2_, 91.6 (d, 1C, C-3′), 84.9, 82.1 (s, 2C, C-1′, C-2′), 80.6 (s, 1C, OC(CH_3_)_3_), 78.1 (d, 1C, C-4), 72.7 (d, 1C, C-5), 67.1 (s, 1C, C-3), 61.5, 61.4 (t, 2C, C-4′, C-6′) 48.4, 47.8 (t, 1C, C-2), 42.9, 42.2 (t, 1C, C-6), 28.8 (q, 1C, OC(CH_3_)_2_), 27.2 (q, 1C, OC(CH_3_)_2_), 25.3 (q, 3C, OC(CH_3_)_3_), 15.5 (q, 2C, C-5′, C-7′). IR (CDCl_3_) ν = 3750, 3649, 3545, 2982, 2933, 2252, 1689, 1456, 1371, 1215, 1163, 1118, 1080, 1051 cm^−1^. C_20_H_33_NO_7_ (399.48): calcd. C, 60.13; H, 8.33; N, 3.51; found C, 60.35; H, 8.20; N, 3.61. MS-ESI (*m*/*z*, %) = 422.14 (100) [M + Na]^+^, 821.08 (68) [2M + Na]^+^.

#### 3.1.13. Synthesis of (3*S*, 4*R*, 5*R*)-3-Hydroxy-4, 5-*O*-(1-Methylethylidene)-3-(3-Thienylethynyl)-*N*-Boc-Piperidine (**30**) 

Application of the general procedure to ketone **8** (30 mg, 0.11 mmol) with 3-ethynylthiophene (22 µL, 0.22 mmol) and *n*-BuLi (67 µL, 0.17 mmol, 2.5 M) gave, after purification by flash column chromatography on silica gel (hexane/AcOEt 4:1), 37 mg of **30** (R_f_ = 0.2, 0.10 mmol, 88%) as a pale-yellow oil.

**30**: $[\alpha {]}_{D}^{22}$ = +44.3 (c = 0.60, CHCl_3_). ^1^H-NMR (400 MHz, CDCl_3_) *δ* ppm: 7.48–7.41 (m, 1H, Ar), 7.26–7.22 (m, 1H, Ar), 7.10–7.04 (m, 1H, Ar), 4.55–4.42 (m, 1H, H-5), 4.39 (d, *J* = 6.8 Hz, 1H, H-4), 3.90–3.60 (m, 3H, Ha-2, H-6), 3.38 (d, *J* = 12.6 Hz, 1H, Hb-2), 2.96 (s, 1H, OH), 1.54 (s, 3H, Me), 1.45 (s, 9H, t-Bu), 1.40 (s, 3H, Me). ^13^C-NMR (100 MHz, CDCl_3_) *δ* ppm: 155.2 (s, 1C, NCOO)_,_ 130.0, 129.9, 125.6 (d, 3C, Ar), 120.9 (s, 1C, Ar), 109.9 (s, 1C, OC(CH_3_)_2_), 87.7, 81.6 (s, 2C, C-1′, C-2′), 80.3 (s, 1C, OC(CH_3_)_3_), 77.0 (d, 1C, C-4), 72.3 (d, 1C, C-5), 67.4 (s, 1C, C-3), 48.3, 47.5 (t, 1C, C-2), 42.7, 41.8 (t, 1C, C-6), 28.5 (q, 3C, OC(CH_3_)_3_)_,_ 27.0 (q, 1C, OC(CH_3_)_2_), 25.0 (q, 1C, OC(CH_3_)_2_). IR (CDCl_3_) ν = 2960, 3702, 3660, 2980, 2252, 1687, 1460, 1406, 1381, 1215, 1161, 1091, 941 cm^−1^. C_19_H_25_NO_5_S (379.47): calcd. C, 60.14; H, 6.64; N, 3.69; S, 8.45; found C, 60.40; H, 6.58; N, 3.81; S, 8.56. MS-ESI (*m*/*z*, %) = 780.96 (100) [2M + Na]^+^, 402.13 (65) [M + Na]^+^.

#### 3.1.14. Synthesis of (3*S*, 4*R*, 5*R*)-3-Hydroxy-4, 5-*O*-(1-Methylethylidene)-3-((4-(Dimethylamino) Phenyl) Ethynyl)-*N*-Boc-Piperidine (**31**)

Application of the general procedure to ketone **8** (50 mg, 0.18 mmol) with 4-ethynyl-*N*, *N*-dimethylaniline (53 mg, 0.37 mmol) and *n*-BuLi (110 µL, 0.28 mmol, 2.5 M) gave, after purification by flash column chromatography on silica gel (hexane/AcOEt 5:1), 62 mg of **31** (R_f_ = 0.4, hexane/AcOEt 2:1, 0.15 mmol, 83%) as orange oil. 

**31**: $[\alpha {]}_{D}^{22}$ = +50.4 (c = 1.00, CHCl_3_). ^1^H-NMR (400 MHz, CDCl_3_) *δ* ppm: 7.25 (d, *J* = 8.2 Hz, 2H, Ar), 6.55 (d, *J* = 8.8 Hz, 2H, Ar), 4.49–4.42 (m, 1H, H-5), 4.35 (d, *J* = 6.8 Hz, 1H, H-4), 3.90–3.57 (m, 3H, Ha-2, H-6), 3.33 (d, *J* = 12.3 Hz, 1H, Hb-2), 3.08 (br s, 1H, OH), 2.93 (s, 6H, N(CH_3_)_2_), 1.51 (s, 3H, Me), 1.43 (s, 9H, t-Bu), 1.38 (s, 3H, Me). ^13^C-NMR (50 MHz, CDCl_3_) *δ* ppm: 155.3 (s, 1C, NCOO)_,_ 150.6 (s, 1C, Ar), 133.1, 111.8 (d, 4C, Ar), 109.7 (s, 1C, OC(CH_3_)_2_), 108.7 (s, 1C, Ar), 87.7, 85.9 (s, 2C, C-1′, C-2′), 80.0 (s, 1C, OC(CH_3_)_3_), 77.3 (d, 1C, C-4), 72.6 (d, 1C, C-5), 67.4 (s, 1C, C-3), 48.5, 47.6 (t, 1C, C-2), 42.7, 41.9 (t, 1C, C-6), 40.2 (q, 2C, N(CH_3_)_2_), 28.6 (q, 3C, OC(CH_3_)_3_)_,_ 27.0, 25.0 (q, 2C, OC(CH_3_)_2_). IR (CDCl_3_) ν = 3541, 2984, 2934, 2249, 2222, 1687, 1607, 1522, 1476, 1452, 1412, 1368, 1215, 1165, 1134, 1072, 943 cm^−1^. C_23_H_32_N_2_O_5_ (416.51): calcd. C, 66.32; H, 7.74; N, 6.73; found C, 66.18; H, 7.68; N, 6.81. MS-ESI (*m*/*z*, %) = 855.20 (100) [2M + Na]^+^, 439.17 (20) [M + Na]^+^.

#### 3.1.15. General Procedure for the Synthesis of Trihydroxypiperidines **32**, **33**, and **34**

A solution of protected compound in MeOH was left stirring with 12 M HCl at room temperature for 18 h. The crude mixture was concentrated to yield the deprotected compound as hydrochloride salt. The corresponding free amine was obtained by dissolving the residue in MeOH, then the strongly basic resin Ambersep 900 OH was added, and the mixture was stirred for 45 min. The resin was removed by filtration and the crude product, if necessary, was purified on silica gel by flash column chromatography to afford the 3-substituted trihydroxypiperidine as free base.

#### 3.1.16. Synthesis of (3*S*, 4*R*, 5*R*)-3, 4, 5-Trihydroxy-3-(Phenylethynyl)-Piperidine (**32**)

Application of the general procedure to **27** (48 mg, 0.13 mmol) with HCl (150 µL) in MeOH (7 mL) furnished, after treatment with Ambersep 900 OH and purification by column chromatography on silica gel (CH_2_Cl_2_/MeOH/NH_4_OH (6%) 10:1:0.1), 16 mg of **32** (R_f_ = 0.2, 0.07 mmol, 53%) as waxy white solid.

**32**: $[\alpha {]}_{D}^{24}$ = −59.1 (c = 0.65, MeOH). ^1^H-NMR (400 MHz, CD_3_OD) *δ* ppm: 7.51–7.43 (m, 2H, Ar), 7.38–7.27 (m, 3H, Ar), 3.98–3.91 (m, 1H, H-5), 3.89 (br s, 1H, H-4), 3.01 (d, *J* = 13.1 Hz, 1H, Ha-2), 2.86–2.76 (m, 3H, Hb-2, H-6). ^13^C-NMR (50 MHz, CD_3_OD) *δ* ppm: 132.7, 129.7, 129.4 (d, 5C, Ar), 123.8 (s, 1C, Ar), 90.7, 87.0 (s, 2C, C-1′, C-2′), 74.8 (d, 1C, C-4), 71.1 (s, 1C, C-3), 69.9 (d, 1C, C-5), 52.7 (t, 1C, C-2), 48.0 (t, 1C, C-6). C_13_H_15_NO_3_ (233.26): calcd. C, 66.94; H, 6.48; N, 6.00; found C, 66.80; H, 6.33; N, 6.07. MS-ESI (*m*/*z*, %) = 234.00 (100) [M + H]^+^.

#### 3.1.17. Synthesis of (3*S*, 4*R*, 5*R*)-3, 4, 5-Trihydroxy-3-(oct-1-yn-1-yl)-Piperidine (**33**)

Application of the general procedure to **28** (120 mg, 0.30 mmol) with HCl (150 µL) in MeOH (7 mL) furnished, after treatment with Ambersep 900 OH and purification by column chromatography on silica gel (CH_2_Cl_2_/MeOH/NH_4_OH (6%) 10:1:0.1), 51 mg of **33** (R_f_ = 0.2, 0.21 mmol, 71%) as a colorless oil.

**33**: $[\alpha {]}_{D}^{25}$ = −23.8 (c = 0.42, CHCl_3_). ^1^H-NMR (400 MHz, CD_3_OD) *δ* ppm: 3.90–3.84 (m, 1H, H-5), 3.75 (br s, 1H, H-4), 2.87 (d, *J* = 13.0 Hz, 1H, Ha-2), 2.78–2.70 (m, 2H, H-6), 2.62 (d, *J* = 13.0 Hz, 1H, Hb-2), 2.25 (t, *J* = 6.8 Hz, 2H, H-3′), 1.57–1.25 (m, 8H, H-4′, H-5′, H-6′, H-7′), 0.92 (t, *J* = 6.8 Hz, 3H, H-8′). ^13^C-NMR (50 MHz, CD_3_OD) δ ppm: 88.0, 81.9 (s, 2C, C-1′, C-2′), 75.1 (d, 1C, C-4), 70.7 (s, 1C, C-3), 69.8 (d, 1C, C-5), 52.7 (t, 1C, C-2), 47.7 (t, 1C, C-6), 32.4, 29.6, 23.6 (t, 4C, C-4′, C-5′, C-6′-C-7′), 19.4 (t, 1C, C-3′), 14.4 (q, 1C, C-8′). C_13_H_23_NO_3_ (241.33): calcd. C, 64.70; H, 9.61; N, 5.80; found C, 64.94; H, 9.49; N, 5.50. MS-ESI (*m*/*z*, %) = 504.92 (100) [2M + Na]^+^, 242.02 (94) [M + H]^+^, 264.14 (47) [M + Na]^+^.

#### 3.1.18. Synthesis of (3*S*, 4*R*, 5*R*)-3, 4, 5-Trihydroxy-3-(3-Thienylethynyl))-Piperidine (**34**)

Application of the general procedure to **30** (85 mg, 0.22 mmol) with HCl (120 µL) in MeOH (6 mL) furnished, after treatment with Ambersep 900 OH and purification by column chromatography on silica gel (gradient eluent from DCM/MeOH/NH_4_OH (6%) 20:1:0.1 to 10:1:0.1), 27 mg of **34** (R_f_ = 0.2, DCM/MeOH/NH_4_OH (6%) 10:1:0.1, 0.11 mmol, 51%) as white waxy solid.

**34**: $[\alpha {]}_{D}^{22}$ = −69.2 (c = 1.00, MeOH). ^1^H-NMR (400 MHz, CD_3_OD) *δ* ppm: 7.63–7.57 (m, 1H, Ar), 7.43–7.38 (m, 1H, Ar), 7.16–7.12 (m, 1H, Ar), 3.95–3.89 (m, 1H, H-5), 3.87 (br s, 1H, H-4), 2.99 (d, *J* = 13.1 Hz, 1H, Ha-2), 2.85–2.74 (m, 3H, Hb-2, H-6). ^13^C-NMR (50 MHz, CD_3_OD) *δ* ppm: 130.8, 130.4, 126.7 (d, 3C, Ar), 122.7 (s, 1C, Ar), 90.2, 82.2 (s, 2C, C-1′, C-2′), 74.8 (d, 1C, C-4), 71.2 (s, 1C, C-3), 69.9 (d, 1C, C-5), 52.8 (t, 1C, C-2), 48.1 (t, 1C, C-6). C_11_H_13_NO_3_S (239.29): calcd. C, 55.21; H, 5.48; N, 5.85; S, 13.40; found C, 55.23; H, 5.61; N, 5.93; S, 13.34. MS-ESI (*m*/*z*, %) = 261.99 (100) [M + Na]^+^.

#### 3.1.19. General Procedure for the Reduction of Triple Bond to Piperidines **11**, **12**, **14**

To a solution of the deprotected compound in EtOH, Pd(OH)_2_/C was added under nitrogen atmosphere. The mixture was stirred at room temperature under hydrogen atmosphere until a ^1^H-NMR analysis attested the presence of aliphatic hydrogens at C-1′ and C-2′ (6–23 h). The catalyst was removed by filtration, the obtained compound was washed several times with EtOH, and the solvent was evaporated under vacuum. The crude product, if necessary, was purified on silica gel by flash column chromatography to afford the corresponding completely reduced 3-substituted trihydroxypiperidine.

#### 3.1.20. Synthesis of (3*S*, 4*R*, 5*R*)-3, 4, 5-Trihydroxy-3-(2-Phenylethyl)-Piperidine (**11**)

Application of the general procedure to **32** (13 mg, 0.05 mmol) with Pd(OH)_2_/C (7 mg) in EtOH (1 mL) furnished, after purification by column chromatography on silica gel (CH_2_Cl_2_/MeOH/NH_4_OH (6%) 10:1:0.1), 5 mg of the corresponding reduced trihydroxypiperidine **11** (R_f_ = 0.2, 0.02 mmol, 39%) as orange oil.

**11**: $[\alpha {]}_{D}^{24}$ = −13.1 (c = 0.36, MeOH). ^1^H-NMR (400 MHz, CD_3_OD) δ ppm: 7.27–7.19 (m, 4H, Ar), 7.18–7.10 (m, 1H, Ar), 3.81 (br s, 1H, H-5), 3.51 (br d, *J* = 2.2 Hz, 1H, H-4), 2.96 (dd, *J* = 4.0, 13.7 Hz, 1H, Ha-6), 2.88 (d, *J* = 13.1 Hz, 1H, Ha-2), 2.76–2.70 (m, 1H, Hb-6), 2.70–2.62 (m, 2H, H-2′), 2.59 (d, *J* =13.1 Hz, 1H, Hb-2), 1.97–1.88 (m, 1H, Ha-1′), 1.86–1.76 (m, 1H, Hb-1′). ^13^C-NMR (100 MHz, CD_3_OD) δ ppm: 143.9 (s, 1C, Ar), 129.4, 129.3, 126.7 (d, 5C, Ar), 74.8 (s, 1C, C-3), 72.7 (d, 1C, C-4), 70.8 (d, 1C, C-5), 54.8 (t, 1C, C-2), 53.5 (t, 1C, C-6), 39.6 (t, 1C, C-1′), 30.7 (t, 1C, C-2′). C_13_H_19_NO_3_ (237.29): calcd. C, 65.80; H, 8.07; N, 5.90; found C, 65.53; H, 8.25; N, 5.81. MS-ESI (*m*/*z*, %) = 238.09 (100) [M + H]^+^, 260.14 (65) [M + Na]^+^, 497.20 (16) [2M + Na]^+^.

#### 3.1.21. Synthesis of (3*S*, 4*R*, 5*R*)-3, 4, 5-Trihydroxy-3-Octyl-Piperidine (**12**)

Application of the general procedure to **33** (40 mg, 0.17 mmol) with Pd(OH)_2_/C (20 mg) in EtOH (2 mL) furnished 41 mg of the corresponding reduced trihydroxypiperidine **12** (0.17 mmol, 98%) as orange oil.

**12**: $[\alpha {]}_{D}^{23}$ = −9.0 (c = 1.00, MeOH). ^1^H-NMR (400 MHz, CD_3_OD) *δ* ppm: 3.77 (br s, 1H, H-5), 3.44 (br s, 1H, H-4), 2.92 (d, *J* = 12.6 Hz, 1H, Ha-6), 2.77 (d, *J* = 13.6, 1H, Ha-2), 2.68 (d, *J* = 12.6 Hz, 1H, Hb-6), 2.50 (d, *J* = 13.6 Hz, 1H, Hb-2), 1.67–1.51 (m, 2H, H-1′), 1.36–1.25 (m, 12H, H-2′, H-3′, H-4′, H-5′, H-6′, H-7′), 0.90 (m, 3H, H-8′). ^13^C-NMR (50 MHz, CD_3_OD) *δ* ppm: 75.1 (s, 1C, C-3), 72.5 (d, 1C, C-4), 70.9 (d, 1C, C-5), 53.6 (t, 1C, C-2), 50.7 (t, 1C, C-6), 37.3 (t, 1C, C-1′), 33.1, 31.5, 30.7, 30.4, 23.9, 23.7 (t, 6C, C-2′, C-3′, C-4′, C-5′, C-6′-C-7′), 14.4 (q, 1C, C-8′). C_13_H_27_NO_3_ (245.36): calcd. C, 63.64; H, 11.09; N, 5.71; found C, 63.78; H, 11.01; N, 5.92. MS-ESI (*m*/*z*, %) = 246.15 (100) [M + H]^+^, 268.26 (22) [M + Na]^+^, 513.00 (10) [2M + Na]^+^.

#### 3.1.22. Synthesis of (3*S*, 4*R*, 5*R*)-3, 4, 5-Trihydroxy-3-((2-(3-Thienyl) Ethyl))-Piperidine (**14**)

Application of the general procedure to **34** (22 mg, 0.09 mmol) with Pd(OH)_2_/C (11 mg) in EtOH (1 mL) furnished, after purification by column chromatography on silica gel (CH_2_Cl_2_/MeOH/NH_4_OH (6%) 5:1:0.1), 16 mg of the corresponding reduced trihydroxypiperidine **14** (R_f_ = 0.2, 0.07 mmol, 73%) as a white solid.

**14**: M.p. = 73–75 °C. $[\alpha {]}_{D}^{20}$ = −19.9 (c = 0.75, MeOH). ^1^H-NMR (400 MHz, CD_3_OD) *δ* ppm: 7.32–7.24 (m, 1H, Ar), 7.06–7.00 (m, 1H, Ar), 7.00–6.94 (m, 1H, Ar), 3.86 (br s, 1H, H-5), 3.53 (br d, *J* = 2.7 Hz, 1H, H-4), 3.02 (dd, *J* = 3.8, 13.6 Hz, 1H, Ha-6), 2.92 (d, *J* = 13.5 Hz, 1H, Ha-2), 2.79 (br d, *J* = 13.6 Hz, 1H, Hb-6), 2.75–2.61 (m, 3H, Hb-2, H-2′), 2.04–1.92 (m, 1H, Ha-1′), 1.92–1.80 (m, 1H, Hb-1′). ^13^C-NMR (100 MHz, CD_3_OD) *δ* ppm: 143.9 (s, 1C, Ar), 129.1, 126.3, 120.8 (d, 3C, Ar), 74.6 (s, 1C, C-3), 72.3 (d, 1C, C-4), 70.4 (d, 1C, C-5), 53.2 (t, 1C, C-2) 50.4 (t, 1C, C-6) 38.3 (t, 1C, C-1′), 24.6 (t, 1C, C-2′). C_11_H_17_NO_3_S (243.32): calcd. C, 54.30; H 7.04; N, 5.76; S, 13.18; found C, 54.11; H, 7.15; N, 5.88; S, 13.33. MS-ESI (*m*/*z*, %) = 243.99 (100) [M + H]^+^, 266.02 (31) [M + Na]^+^, 508.78 (30) [2M + Na]^+^.

#### 3.1.23. Synthesis of (3*S*, 4*R*, 5*R*)-3-hydroxy-4, 5-*O*-(1-methylethylidene)-3-(2-((4-(dimethylamino) phenyl) ethyl)-*N*-Boc-piperidine (**35**)

To a solution of **31** (75 mg, 0.18 mmol) in EtOH (2 mL), Pd/C (37 mg) was added under nitrogen atmosphere. The mixture was stirred at room temperature under hydrogen atmosphere until a ^1^H-NMR analysis attested the presence of aliphatic hydrogens at C-1′ and C-2′ (2 h). The catalyst was removed by filtration, the obtained compound was washed several times with EtOH, and the solvent was evaporated under vacuum. The crude product was purified on silica gel by flash column chromatography (hexane:AcOEt 2:1) to afford 54 mg of the corresponding reduced piperidine **35** (R_f_ = 0.3, 0.13 mmol, 71%) as a colorless oil.

**35**: $[\alpha {]}_{D}^{22}$ = +6.3 (c = 0.60, CHCl_3_). ^1^H-NMR (400 MHz, CDCl_3_) *δ* ppm: 7.07 (d, *J* = 8.3 Hz, 2H, Ar), 6.69 (d, *J* = 8.3 Hz, 2H, Ar), 4.30 (br s, 1H, H-5), 3.98 (d, *J* = 6.4 Hz, 1H, H-4), 3.92–3.70 (m, 1H, Ha-2), 3.68–3.55 (m, 1H, Ha-6), 3.43–3.10 (m, 2H, Hb-2, Hb-6), 2.90 (s, 6H, N(CH_3_)_2_), 2.78–2.64 (m, 2H, H-2′), 1.81–1.66 (m, 2H, H-1′), 1.54 (s, 3H, Me), 1.47 (s, 9H, t-Bu), 1.38 (s, 3H, Me). ^13^C-NMR (100 MHz, CDCl_3_) *δ* ppm: 155.0 (s, 1C, NCOO)_,_ 149.2 (s, 2C, Ar), 129.0, 113.3 (d, 4C, Ar), 109.4 (s, 1C, OC(CH_3_)_2_), 80.3 (s, 1C, OC(CH_3_)_3_), 77.9 (d, 1C, C-4), 72.0 (d, 1C, C-5), 70.7 (s, 1C, C-3), 47.2, 46.4 (t, 1C, C-6), 43.7, 42.9 (t, 1C, C-2), 41.0 (q, 2C, N(CH_3_)_2_), 40.5, 39.6 (t, 1C, C-1′), 28.6 (q, 3C, OC(CH_3_)_3_)_,_ 28.0 (t, 1C, C-2′) 27.4 (q, 1C, OC(CH_3_)_2_), 25.3 (q, 1C, OC(CH_3_)_2_). IR (CDCl_3_) ν = 3561, 2984, 2934, 2803, 2247, 1684, 1614, 1520, 1456, 1416, 1375, 1346, 1246, 1215, 1165, 1136 1059 cm^−1^. C_23_H_36_N_2_O_5_ (420.54): calcd. C, 65.69; H 8.63; N, 6.66; found C, 65.58; H, 8.78; N, 6.50. MS-ESI (*m*/*z*, %) = 443.10 (100) [M + Na]^+^.

#### 3.1.24. Synthesis of (3*S*, 4*R*, 5*R*)-3, 4, 5-Trihydroxy-3-(2-((4-(Dimethylamino) Phenyl) Ethyl)-Piperidine (**15**) 

A solution of **35** (53 mg, 0.13 mmol) in MeOH (6 mL) was stirred with 12 M HCl (120 µL) at room temperature for 18 h. The crude mixture was concentrated to yield **15** as hydrochloride salt. The corresponding free amine was obtained by dissolving the residue in MeOH, then the strongly basic resin Ambersep 900 OH was added, and the mixture was stirred for 45 min. The resin was removed by filtration and the crude product was purified on silica gel by flash column chromatography (CH_2_Cl_2_/MeOH/NH_4_OH (6%) 10:1:0.1) to afford 36 mg of the 3-substituted trihydroxypiperidine **15** (R_f_ = 0.1, 0.13 mmol, 100% yield) as a waxy colorless solid.

**15**: $[\alpha {]}_{D}^{20}$ = −21.8 (c = 1.00, MeOH). ^1^H-NMR (400 MHz, CD_3_OD) *δ* ppm: 7.06 (d, *J* = 8.2 Hz, 2H, Ar), 6.72 (d, *J* = 8.2 Hz, 2H, Ar), 3.80 (br s, 1H, H-5), 3.49 (br s, 1H, H-4), 2.95 (d, *J* = 13.2 Hz, 1H, Hb-6), 2.91–2.75 (m, 7H, Ha-2, N(CH_3_)_2_), 2.70 (d, *J* = 13.8 Hz, 1H, Hb-6), 2.63–2.44 (m, 3H, Hb-2, H-2′), 1.94–1.71 (m, 2H, H-1′). ^13^C-NMR (100 MHz, CD_3_OD) *δ* ppm: 150.6, 132.7 (s, 2C, Ar), 129.8, 114.9 (d, 4C, Ar), 74.9 (s, 1C, C-3), 72.6 (d, 1C, C-4), 70.8 (d, 1C, C-5), 53.6 (t, 1C, C-2), 50.6 (t, 1C, C-6), 41.6 (q, 2C, N(CH_3_)_2_), 39.7 (t, 1C, C-1′), 29.2 (t, 1C, C-2′). C_15_H_24_N_2_O_3_ (280.36): calcd. C, 64.26; H, 8.63; N, 9.99; found C, 64.10; H, 8.33; N, 9.63. MS-ESI (*m*/*z*, %) = 582.84 (100) [2M + Na]^+^, 281.04 (59) [M + H]^+^.

### 3.2. Preliminary Biological Screening towards Commercial Glycosidases

A panel of 12 commercial glycosidases (α-l-fucosidase EC 3.2.1.51 from Homo sapiens, α-galactosidase EC 3.2.1.22 from coffee beans, β-galactosidases EC 3.2.1.23 from *Escherichia coli* and *Aspergillus oryzae*, α-glucosidases EC 3.2.1.20 from yeast and rice, amyloglucosidase EC 3.2.1.3 from *Aspergillus niger*, β-glucosidase EC 3.2.1.21 from almonds, α-mannosidase EC 3.2.1.24 from Jack beans, β-mannosidase EC 3.2.1.25 from snail, β-*N*-acetylglucosaminidases EC 3.2.1.52 from Jack beans and bovine kidney) purchased from Sigma-Aldrich (St. Louis, MO, USA) or Megazyme Ltd. (Bray, Co. Wicklow, Ireland) was screened.

The percentage (%) of inhibition towards the corresponding glycosidase was determined in quadruplicate in the presence of 100 μM of the inhibitor on the well. Each enzymatic assay (final volume 0.12 mL) contains 0.01–0.5 units/mL of the enzyme (with previous calibration) and 4.2 mM aqueous solution of the appropriate *p*-nitrophenyl glycopyranoside (substrate) buffered to the optimal pH of the enzyme. Enzyme and inhibitor were preincubated for 5 min at room temperature, and the reaction started by addition of the substrate. After 20 min of incubation at 37 °C, the reaction was stopped by the addition of 0.1 mL of sodium borate solution (pH 9.8). The *p*-nitrophenolate formed was measured by visible absorption spectroscopy at 405 nm (Asys Expert 96 spectrophotometer, Biochrom Ltd., Cambridge, England). Under these conditions, the *p*-nitrophenolate released led to optical densities linear with both reaction time and concentration of the enzyme (for more details, see the Supplementary Materials). The IC_50_ value (concentration of inhibitor required for 50% inhibition of enzyme activity) was determined from plots of % inhibition vs. different inhibitor concentrations. Each point of the graph is the average of four measures. The standard deviation for each point has been also represented in the graph.

### 3.3. Biological Screening towards Human Lysosomal β-Galactosidase (β-Gal) and β-Glucosidase (GCase)

All experiments on biological materials were performed in accordance with the ethical standards of the institutional research committee and with the 1964 Helsinki Declaration and its later amendments. In keeping with ethical guidelines, all blood and cell samples were obtained for storage and analyzed only after written informed consent of the patients (and/or their family members) was obtained, using a form approved by the local Ethics Committee (Project ID code: 16774_bio, 5 May 2020, Comitato Etico Regionale per la Sperimentazione Clinica della Regione Toscana, Area Vasta Centro, Florence, Italy). Controls and patients’ samples were anonymized and used only for research purposes.

The new compounds were screened at 1 mM concentration towards β-galactosidase (β-Gal) and β-glucosidase (GCase) in leukocytes isolated from healthy donors (controls). Isolated leukocytes were disrupted by sonication, and a Micro BCA Protein Assay Kit (Sigma–Aldrich, St. Louis, MO, USA) was used to determine the total protein amount for the enzymatic assay, according to the manufacturer’s instructions.

#### 3.3.1. Human Lysosomal β-Galactosidase (β-Gal) Activity

β-Gal activity was measured in a flat-bottomed 96-well plate. Azasugar solution (3 μL), 4.29 μg/μL leukocytes homogenate 1:10 (7 μL), and substrate 4-methylumbelliferyl β-d-galactopyranoside (1.47 mM, 20 μL, Sigma–Aldrich) in acetate buffer (0.1 M, pH 4.3) containing NaCl (0.1 M) and sodium azide (0.02%) were incubated at 37 °C for 1 h. The reaction was stopped by addition of sodium carbonate (200 μL; 0.5 M, pH 10.7) containing Triton X-100 (0.0025%), and the fluorescence 4-methylumbelliferone released by β-galactosidase activity was measured in SpectraMax M2 Microplate Reader (λex = 365 nm and λem = 435 nm; Molecular Devices LLC, San Jose, CA, US). Inhibition is given with respect to the control (without azasugar). Data are mean SD (*n* = 3).

#### 3.3.2. Human Lysosomal β-Glucosidase (GCase) Activity

GCase activity was measured in a flat-bottomed 96-well plate. Compound solution (3 µL), 4.29 µg/µL leukocytes homogenate (7 µL), and substrate 4-methylumbelliferyl-β-d-glucoside (3.33 mM, 20 µL, Sigma–Aldrich Chemie Gmbh, Munich, Germany)) in citrate/phosphate buffer (0.1:0.2, M/M, pH 5.8) containing sodium taurocholate (0.3%) and Triton X-100 (0.15%) at 37 °C were incubated for 1 h. The reaction was stopped by addition of sodium carbonate (200 µL; 0.5 M, pH 10.7) containing Triton X-100 (0.0025%), and the fluorescence of 4-methylumbelliferone released by β-glucosidase activity was measured in SpectraMax M2 Microplate Reader (λex = 365 nm and λem = 435 nm; Molecular Devices LLC, San Jose, CA, US). Percentage GCase inhibition is given with respect to the control (without compound). Data are mean SD (*n* = 3). For compounds showing GCase inhibitory activity higher than 80% at 1 mM concentration, the IC_50_ values were determined by measuring the initial hydrolysis rate with 4-methylumbelliferyl-β-d-glucoside (3.33 mM). Data obtained were fitted by using the appropriate equation (for more details, see the Supplementary Materials).

### 3.4. Pharmacological Chaperoning Activity of Compound **10**

Anonymized patient fibroblasts cell lines for research purposes were provided by “Cell Line and DNA Biobank from Patients Affected by Genetic Diseases” (G. Gaslini Institute)-Telethon Genetic Biobank Network (Project No. GTB07001A).

Fibroblasts with the N370S/RecNcil mutation from patients with Gaucher disease were obtained from the “Cell line and DNA Biobank from patients affected by Genetic Diseases” (Gaslini Hospital, Genova, Italy). Fibroblasts cells (15.0 × 10^4^) were seeded in T25 flasks with DMEM supplemented with fetal bovine serum (10%), penicillin/streptomycin (1%), and glutamine (1%) and incubated at 37 °C with 5% CO_2_ for 24 h. The medium was removed, and fresh medium containing compound **10** was added to the cells and left for 4 days. The medium was removed, and the cells were washed with PBS and detached with trypsin to obtain cell pellets, which were washed four times with PBS, frozen, and lysed by sonication in water. Enzyme activity was measured as reported above. Reported data are mean S.D. (*n* = 2).

## 4. Conclusions

In conclusion, with the aim of searching for new human lysosomal β-Gal inhibitors, we synthesized a series of trihydroxypiperidines and congeners with the “*all-cis*” configuration at the carbons bearing the hydroxy/amino groups. The reported syntheses exploited the reaction of ketone intermediate **8**, which was functionalized at C=O through ketone reduction followed by alcohol alkylation (**9**, **21**), reductive amination (**10**), or via the stereoselective addition of various lithium acetylides (**11**–**15**, **32,** and **33**). 

Unfortunately, none of the reported compounds showed β-Gal inhibition. However, these data give useful pointers, suggesting that the presence of three “*all-cis”* hydroxy groups at C-3, C-4, and C-5 of the piperidine ring is essential for β-Gal inhibitory activity, but that the introduction of an alkyl chain at C-3, either through oxygen (or nitrogen) or directly to carbon (as in compound **12**), hampers interaction with the enzyme active site. 

Interestingly, newly synthesized compounds **9**, **10**, and **12** showed moderate to good inhibition against human lysosomal β-glucosidase (GCase). For this enzyme, our data confirmed that the presence of an alkyl chain of at least eight carbon atoms is essential to impart GCase inhibitory activity. However, we show that the alkyl chain can be placed at the C-3 position either maintaining the three hydroxyl groups (as in compound **12**) or via alkylation of the hydroxy group at C-3 (as in ether **9**). Compound **10**, the best GCase inhibitor synthesized in this work, failed to rescue the enzyme activity on cell lines likely due to its cytotoxicity. Nevertheless, this study provided useful hints for the design of novel inhibitors for both GCase and β-Gal.

## Figures and Tables

**Table 1:**
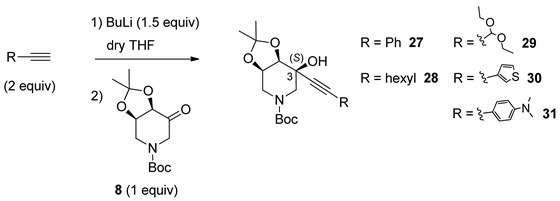
Addition reactions of lithium acetylides to ketone **8**.

Entry ^a^	Alkyne	Time (h)	Product	Yield (%)
1	Phenylacetylene	2.5	**27**	77
2	1-octyne	3	**28**	78
3	3,3-diethoxyprop-1-yne	3	**29**	65
4	3-ethynylthiophene	4	**30**	88
5	4-ethynyl-*N*,*N*-dimethylaniline	4	**31**	83

^a^: All the experiments were carried out in dry THF with temperatures ranging from −78 °C to room temperature, with ketone **8** (1.0 equiv.) and BuLi (1.5 equiv.).

**Table 2:** Chemical shifts and coupling constants of H-4, H-5, and H-6 of protected compounds **26** and **35**, in CDCl_3_.

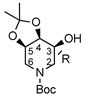	H-4	H-5	H-6a	H-6b
	δ (ppm)	δ (ppm)	δ (ppm)	δ (ppm)
R = vinyl, **26**	4.07 (d, J = 6.8 Hz)	4.33 (br s)	3.95–3.69 (m)	3.53–3.32 (m)
R = 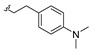 , **35**	3.98 (d, J = 6.4 Hz)	4.30 (br s)	3.68–3.55 (m)	3.43–3.10 (m)

**Table 3:** Chemical shifts and coupling constants of H-4, H-5, and H-6 of protected compounds **32** and **11**–**15**, in CD_3_OD.

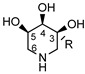	H-4	H-5	H-6a	H-6b
	δ (ppm)	δ (ppm)	δ (ppm)	δ (ppm)
R = 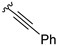 , **32**	3.89 (br s)	3.98–3.91 (m)	2.86–2.76 (m)	
R = 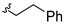 , **11**	3.51 (br d, J = 2.2 Hz)	3.81 (br s)	2.96 (dd, J = 13.7, 4.0 Hz)	2.76–2.70 (m)
R = octyl, **12**	3.44 (br s)	3.77 (br s)	2.92 (d, J = 12.6 Hz)	2.68 (d, J = 12.6 Hz)
R = ethyl, **13**	3.47 (d, J = 3.2 Hz)	3.81 (br s)	2.96 (dd, J = 13.6, 3.4 Hz)	2.72 (dd, J = 13.7, 2.4 Hz)
R = 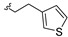 , **14**	3.53 (br d, J = 2.7 Hz)	3.86 (br s)	3.02 (dd, J = 13.6, 3.8 Hz)	2.79 (br d, J = 13.6 Hz)
R = 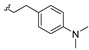 **, 15**	3.49 (br s)	3.80 (br s)	2.95 (d, J = 13.2 Hz)	2.70 (d, J = 13.8 Hz)

**Table 4:** β-galactosidase (β-Gal) and β-glucosidase (GCase) inhibition in human leukocytes from healthy donors.

Entry	Compound	β-Gal	GCase	
		Inhibition (%) ^a^	Inhibition (%) ^a^	IC_50_ (µM) ^b^
1	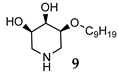	22	98	12 ± 6
2	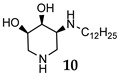	0	100	6.4 ± 0.7
3	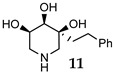	0	36	n.d.
4	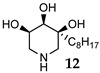	0	93	60 ± 23
5	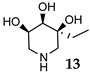	3	30	n.d.
6	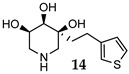	16	9	n.d.
7	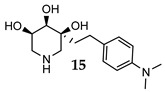	6	6	n.d.
8	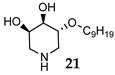	0	100	130 ± 13
9	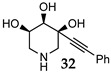	0	25	n.d.
10	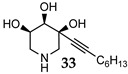	4	65	n.d.
11	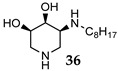	15	93 ^c^	40 ± 3 ^c^
12	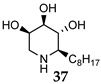	14 ^d^	100 ^d^	29 ± 2 ^d^
13	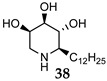	5 ^d^	100 ^d^	1.5 ± 0.1 ^d^
14	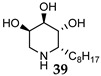	31 ^d^	80 ^d^	94 ± 5 ^d^

^a^ Percentage inhibition of β-galactosidase (β-Gal) and β-glucosidase (GCase) in human leukocytes extracts incubated with compounds (1 mM). ^b^ IC_50_ values were determined by measuring GCase activity at different concentrations of each inhibitor for compounds showing inhibitory activity higher than 70% at 1 mM (n.d. = not determined). ^c^ Ref. [22] ^d^ Ref. [24].

## Supplementary Materials

Figures S1–S44 containing ^1^H and ^13^C-NMR of new compounds, Table S1: Addition of sp^3^ Grignard reagents to ketone **8**. Table S2: Configuration assignment. Table S3: Biological screening towards commercial glycosidases. Figure S45: IC_50_ for compound **10** towards β-glucosidase from almonds. Figure S46 and Figure S47: Biological screening towards human lysosomal β-Gal and GCase. Figures S48–S51: IC_50_ for compounds **9**, **10**, **12**, and **21** towards GCase. Figure S52 to Figure S54: Chaperoning activity assay of compounds **9**, **10**, and **12**.
