# Supplementary material for: Synthesis of “All-Cis” Trihydroxypiperidines from a Carbohydrate-Derived Ketone: Hints for the Design of New β-Gal and GCase Inhibitors
Source: Molecules. 2020 Oct 2;25(19):4526. doi: 10.3390/molecules25194526 (PMC7582770; doi:10.3390/molecules25194526)
Supplement: Supplementary file 1 [file molecules-25-04526-s001.pdf]

Synthesis of "*all-cis*" trihydroxypiperidines from a carbohydrate derived ketone: hints for the design of new β-Gal and GCase inhibitors

Maria Giulia Davighi [1], Francesca Clemente [1], Camilla Matassini*[1], Amelia Morrone [2], Andrea Goti [1,3], Macarena Martínez-Bailén[4] and Francesca Cardona [1,3*]

[1] Department of Chemistry "Ugo Schiff", University of Firenze, via della Lastruccia 3-13, 50019 Sesto Fiorentino (FI), Italy; mariagiulia.davighi@unifi.it (M.G.D.); francesca.clemente@unifi.it (F.Cl.); andrea.goti@unifi.it (A.G.)

[2] Paediatric Neurology Unit and Laboratories, Neuroscience Department, Meyer Children's Hospital, and Department of Neurosciences, Pharmacology and Child Health. University of Florence, Viale Pieraccini n. 24, 50139 Firenze, Italy; amelia.morrone@unifi.it (A.M.)

[3] Associated with Consorzio Interuniversitario Nazionale di ricerca in Metodologie e Processi Innovativi di Sintesi (CINMPIS)

[4] Departamento de Química Orgánica, Facultad de Química, Universidad de Sevilla, c/ Prof. García González 1, E-41012 Sevilla, Spain, mmartinez45@us.es (M. M.B.)

* Correspondence: francesca.cardona@unifi.it (F.Ca.), camilla.matassini@unifi.it (C.M.); Tel: +39-055-4573504 (F.Ca.)

Supplementary Materials

Table of contents

Figures S1-S2: ^{1}H-NMR and ^{13}C-NMR spectra of compound **19**	S3
Figures S3-S4: ^{1}H-NMR and ^{13}C-NMR spectra of compound **9**	S4
Figures S5-S6: ^{1}H-NMR and ^{13}C-NMR spectra of compound **20**	S5
Figures S7-S8: ^{1}H-NMR and ^{13}C-NMR spectra of compound **21**	S6
Figures S9-S10: ^{1}H-NMR and ^{13}C-NMR spectra of compound **22**	S7
Figures S11-S12: ^{1}H-NMR and ^{13}C-NMR spectra of compound **10**	S8
Table S1: Reactions of sp^3 Grignard reagents to ketone **8**	S9
Synthesis and characterization of acetylated compound **40**	S10
Figures S13-S14: ^{1}H-NMR and ^{13}C-NMR spectra of acetylated compound **40**	S11
Figures S15-S16: ^{1}H-NMR and ^{13}C-NMR spectra of compound **26**	S12
Figures S17-S18: ^{1}H-NMR and ^{13}C-NMR spectra of compound **13**	S13
Figures S19-S20: ^{1}H-NMR and ^{13}C-NMR spectra of compound **27**	S14
Figures S21-S22: ^{1}H-NMR and ^{13}C-NMR spectra of compound **28**	S15
Figures S23-S24: ^{1}H-NMR and ^{13}C-NMR spectra of compound **29**	S16
Figures S25-S26: ^{1}H-NMR and ^{13}C-NMR spectra of compound **30**	S17
Figures S27-S28: ^{1}H-NMR and ^{13}C-NMR spectra of compound **31**	S18
Figures S29-S30: ^{1}H-NMR and ^{13}C-NMR spectra of compound **32**	S19
Figures S31-S32: ^{1}H-NMR and ^{13}C-NMR spectra of compound **33**	S20
Figures S33-S34: ^{1}H-NMR and ^{13}C-NMR spectra of compound **34**	S21
Figures S35-S36: ^{1}H-NMR and ^{13}C-NMR spectra of compound **11**	S22
Figures S37-S38: ^{1}H-NMR and ^{13}C-NMR spectra of compound **12**	S23
Figures S39-S40: ^{1}H-NMR and ^{13}C-NMR spectra of compound **14**	S24
Figures S41-S42: ^{1}H-NMR and ^{13}C-NMR spectra of compound **35**	S25
Figures S43-S44: ^{1}H-NMR and ^{13}C-NMR spectra of compound **15**	S26
Table S2 Configuration assignment	S27-S28
Table S3: Biological screening towards commercial glycosidases	S29
Figure S45: IC_{50} for compound **10** towards β-glucosidase from almonds	S30
Figure S46: Biological screening towards human lysosomal β-galactosidase	S31
Figure S47: Biological screening towards human lysosomal β-glucosidase	S31
Figures S48-S51: IC_{50} for compounds **9**, **10**, **12** and **21** towards GCase	S32-S33
Figures S52-S54: Chaperoning activity assays of compound **9, 10** and **12**	S34-S35

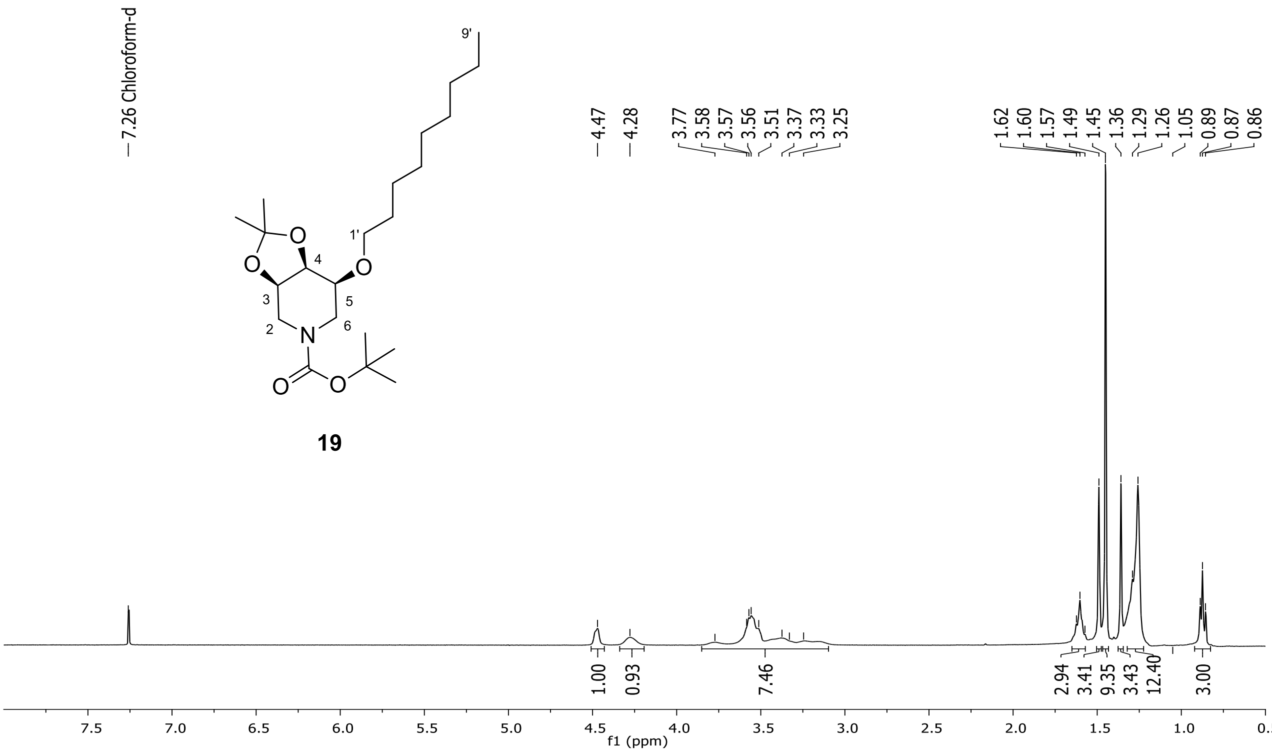

Figure S1: ^{1}H NMR spectrum of 19 (400 MHz, $CDCl_3$)

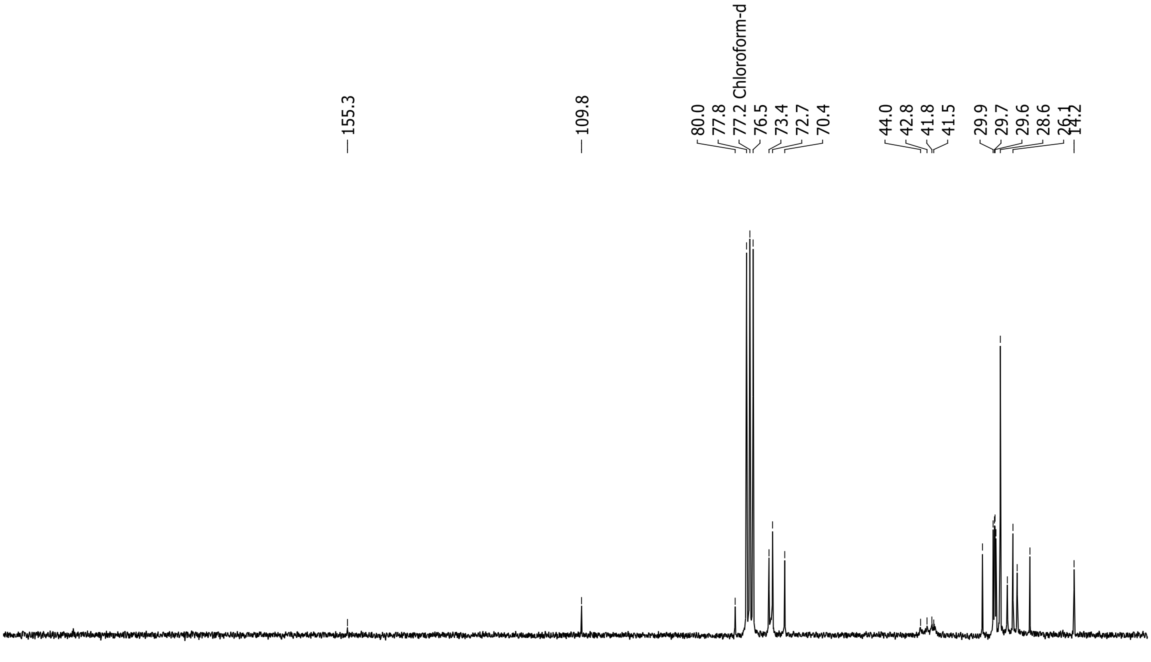

Figure S2: ^{13}C NMR spectrum of 19 (50 MHz, $CDCl_3$)

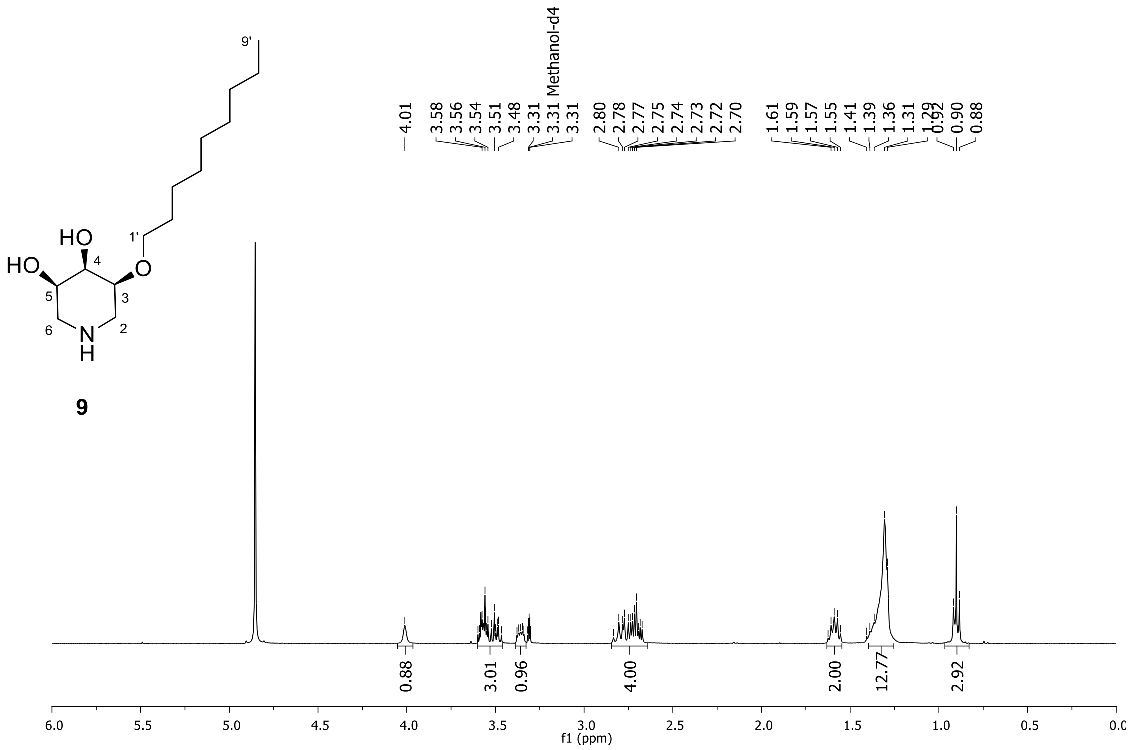

Figure S3: ^{1}H NMR spectrum of 9 (400 MHz, CD_3OD)

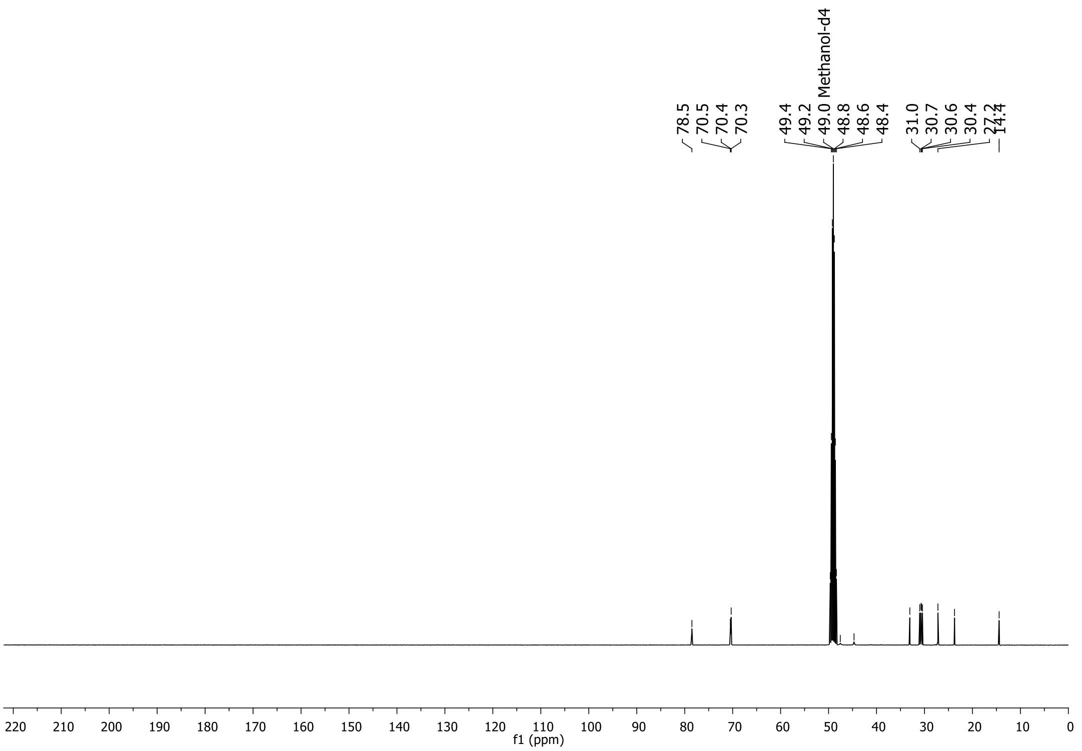

Figure S4: ^{13}C NMR spectrum of 9 (100 MHz, CD_3OD)

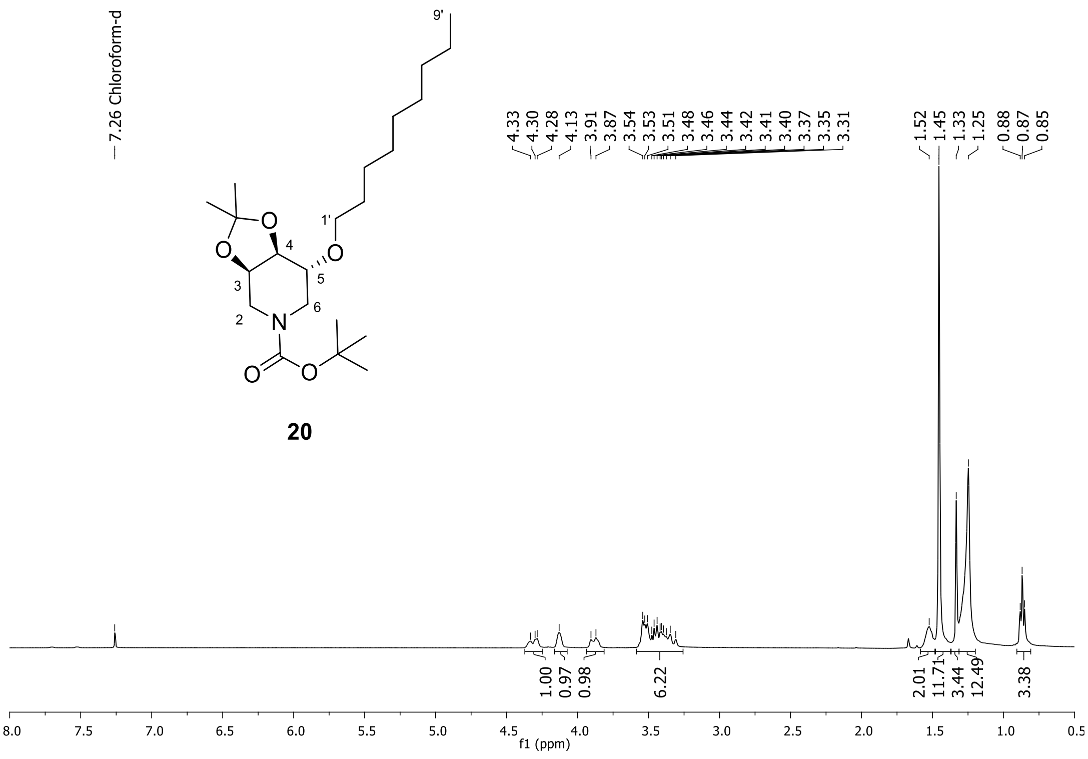

Figure S5: ^{1}H NMR spectrum of 20 (400 MHz, $CDCl_3$)

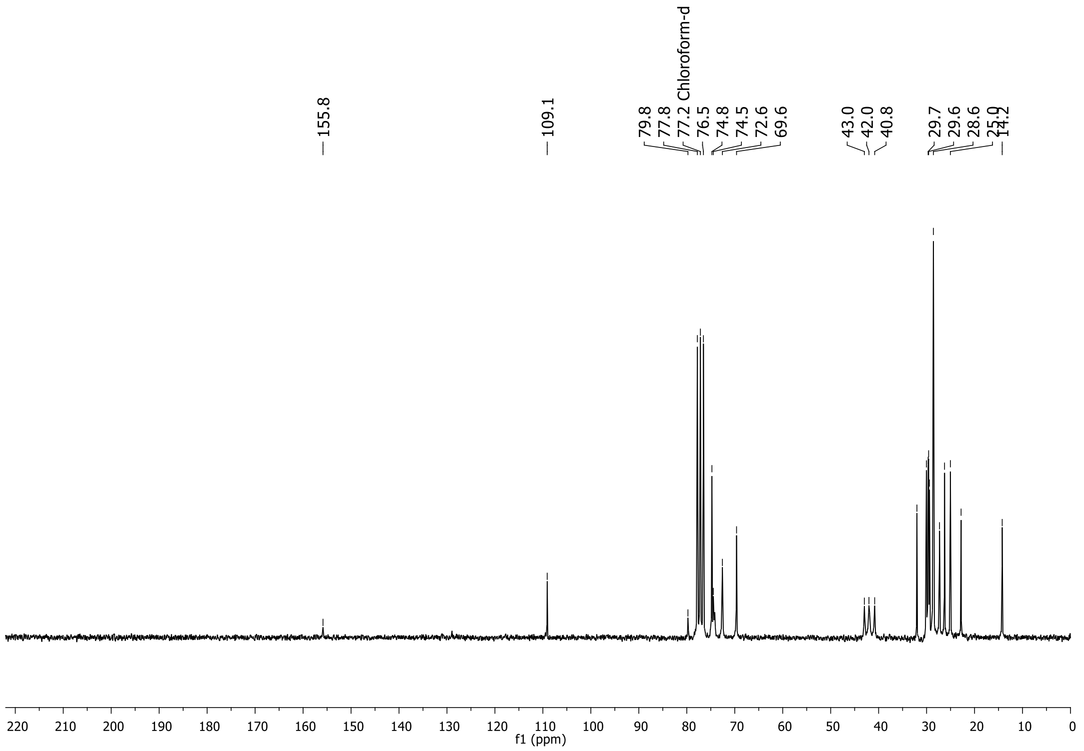

Figure S6: ^{13}C NMR spectrum of 20 (50 MHz, $CDCl_3$)

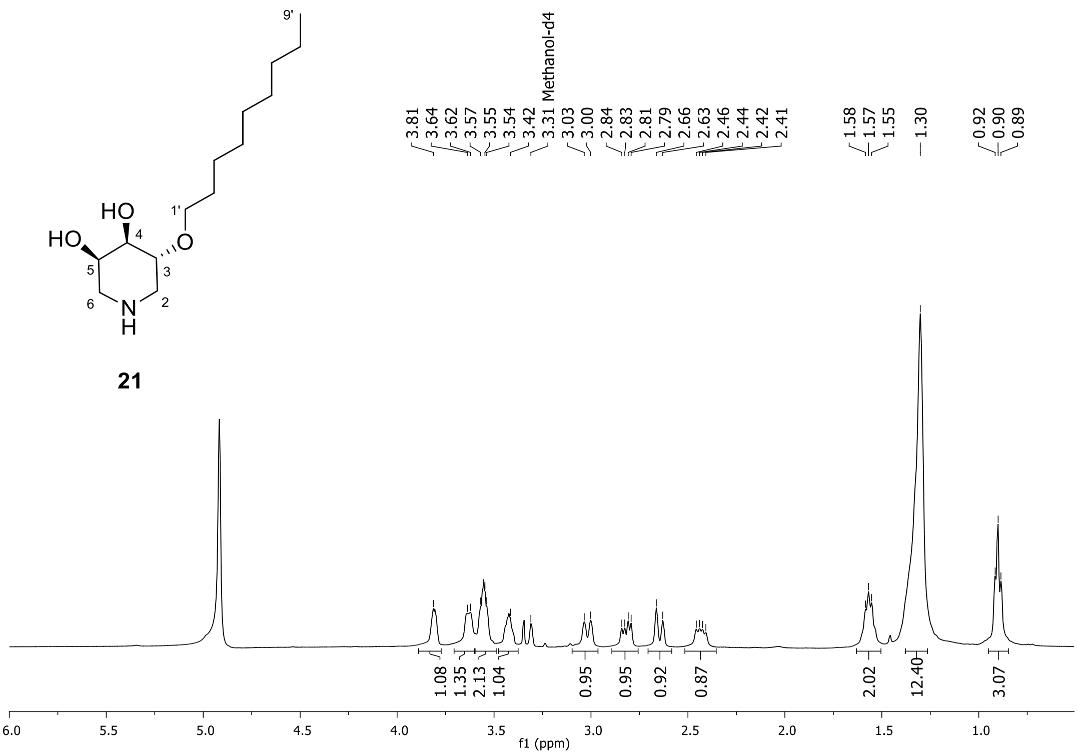

Figure S7: ^{1}H NMR spectrum of 21 (400 MHz, CD_3OD)

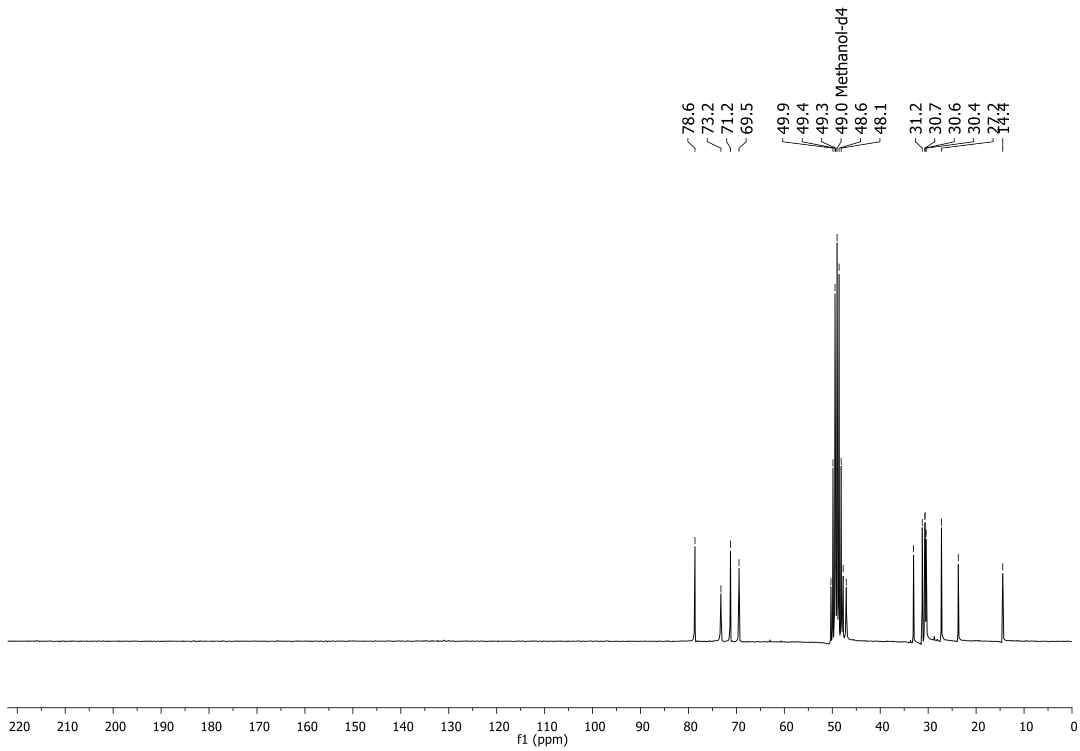

Figure S8: ^{13}C NMR spectrum of 21 (50 MHz, CD_3OD)

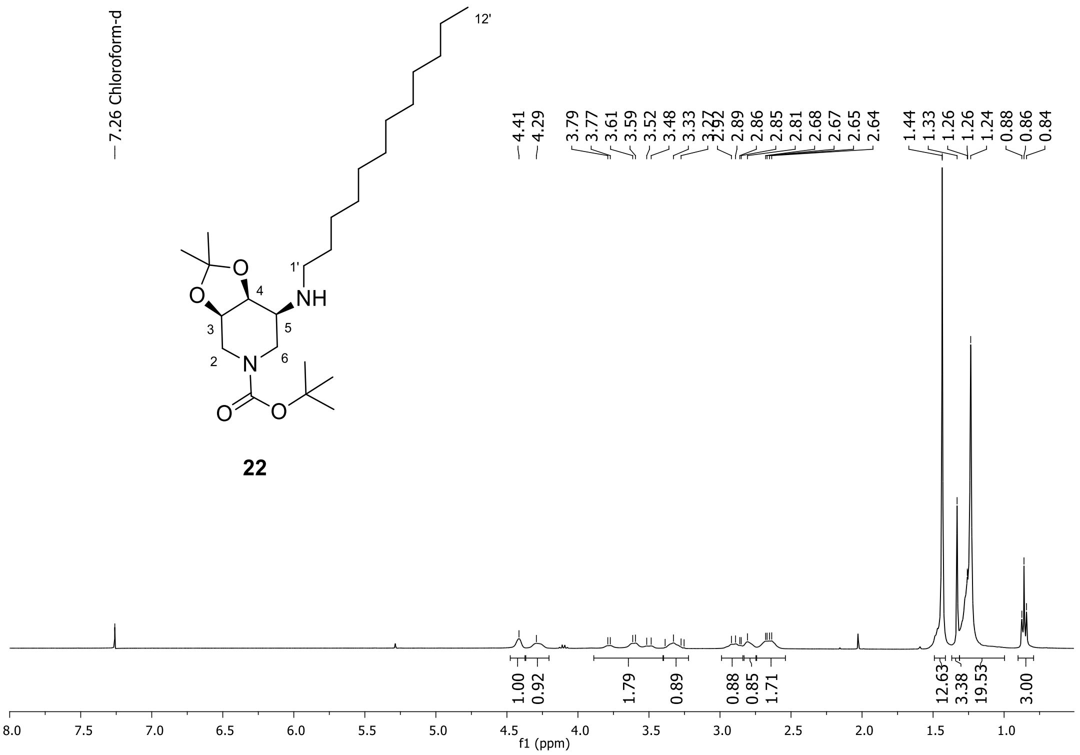

Figure S9: ^{1}H NMR spectrum of 22 (400 MHz, $CDCl_3$)

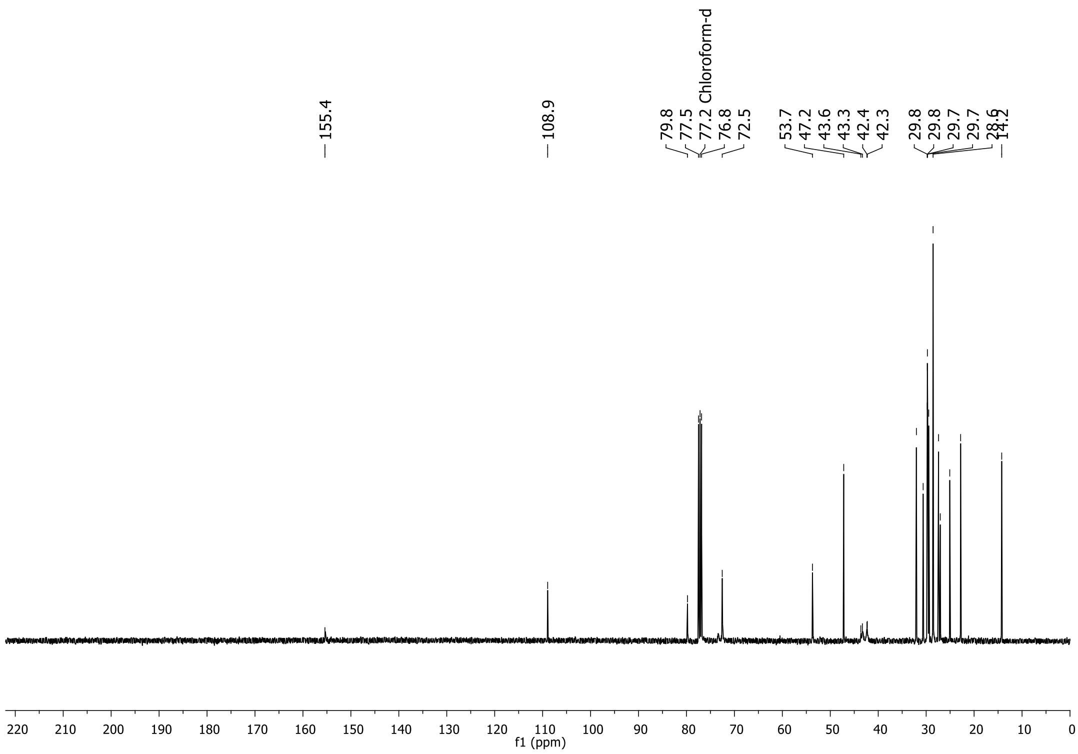

Figure S10: ^{13}C NMR spectrum of 22 (100 MHz, $CDCl_3$)

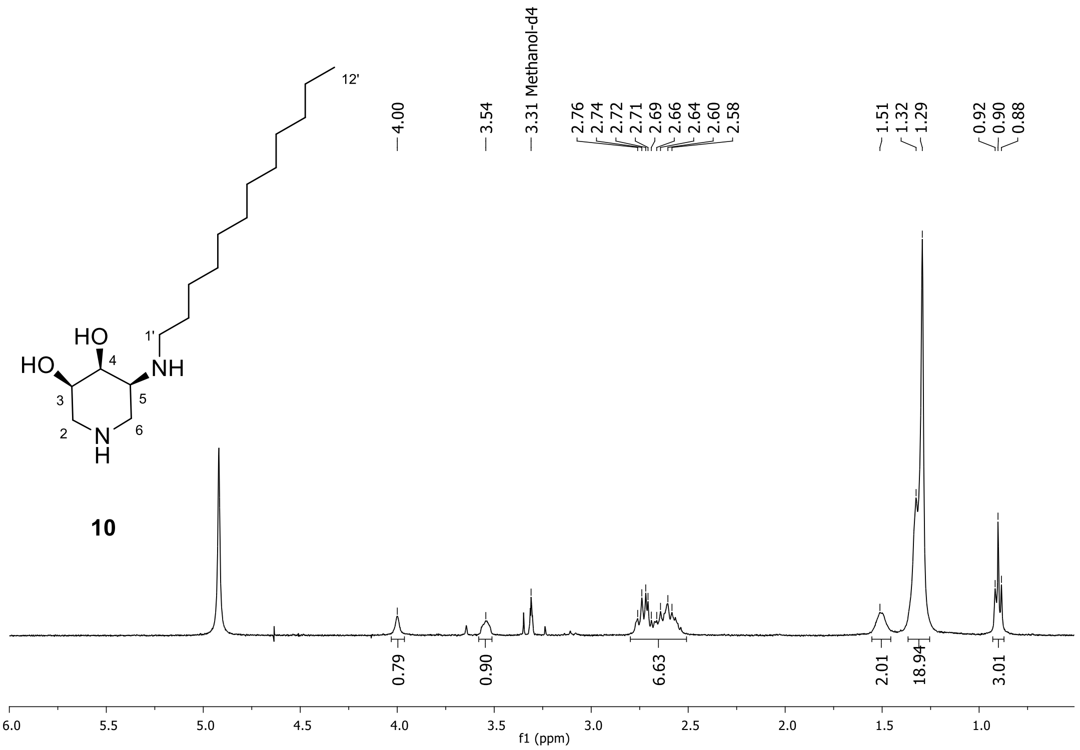

Figure S11: ^{1}H NMR spectrum of 10 (400 MHz, CD_3OD)

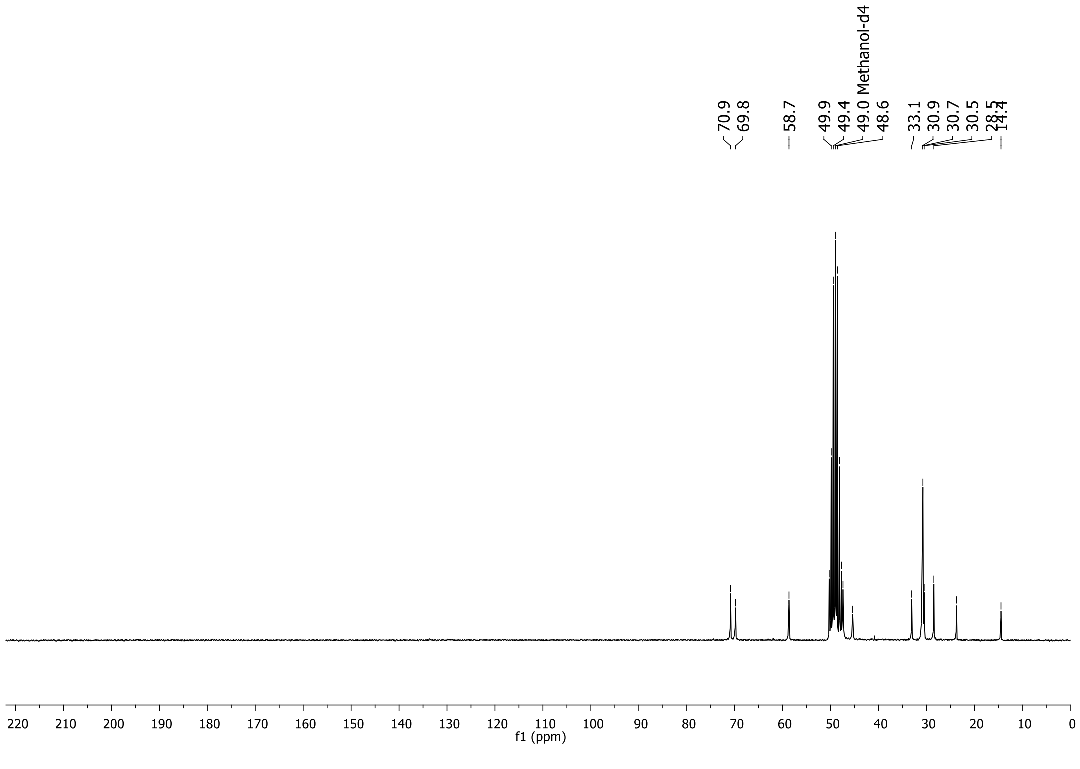

Figure S12: ^{13}C NMR spectrum of 10 (50 MHz, CD_3OD)

Table S1. Addition of sp^3 Grignard reagents to ketone **8**.

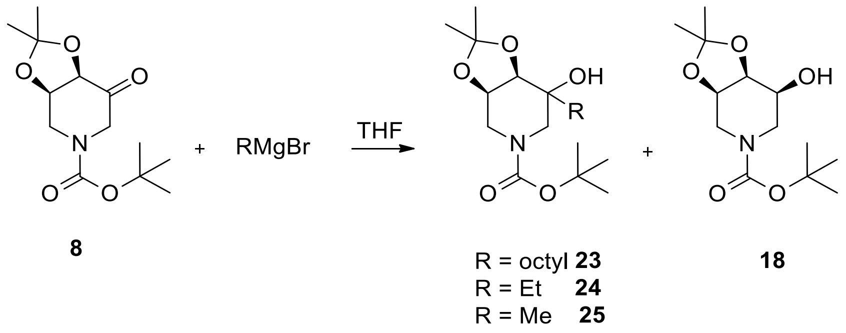

Entry	R	Equiv. Grignard	Temperature	Additive	Time (h)	Yield (%)
1	octyl	1	-78 °C	-	3	< 10
2	octyl	1.8	-78 °C	-	3	< 10
3	octyl	1.5	0 °C	-	5	< 10
4	octyl	1.5	-30 °C → r.t.	$BF_3 \cdot Et_2O$	15	-
5	Et	1.3	-78 °C → r.t.	-	21	9
6	Me	1.3	0 °C → r.t.	-	3	12

General procedure for the addition of different Grignard reagents to ketone 8

To a dry THF solution (0.2 M) of ketone **8** (1 eq.) with or without Lewis acid ($BF_3 \cdot Et_2O$) (1.1 eq.), the Grignard reagent was added dropwise at low temperature (See Table 1) under nitrogen atmosphere. The solution was stirred at different temperatures (See Table S1) until the disappearance of **8** was attested by a TLC control (3-21 h). A saturated aqueous NH_4Cl solution was added to the mixture and left stirring for 10 minutes. The reaction mixture was then extracted with Et_2O. The combined organic extracts were washed with brine and concentrated under reduced pressure after drying with Na_2SO_4. The crude residue was purified by flash column chromatography on silica gel to afford the impure **23**, **24** and **25**, respectively (see Table S1).

Synthesis of alcohols 23 and 18

To a dry THF solution (1 mL) of ketone **8** (52 mg, 0.19 mmol), octylmagnesium bromide (143 µL, 0.29 mmol, 2 M) was added dropwise at 0 °C under nitrogen atmosphere. The solution was stirred at 0 °C for 5 h (disappearance of **8** was attested by a TLC control with hexane/AcOEt 2:1). A saturated aqueous NH_4Cl solution was added to the mixture at 0 °C and left stirring for 10 minutes. The reaction mixture was extracted with Et_2O (3×3 ml). The combined organic extracts were washed with brine and concentrated under reduced pressure after drying with Na_2SO_4. The crude residue was purified by flash column chromatography on silica gel (gradient eluent from hexane/AcOEt 20:1 to 15:1) to afford 7 mg of impure **23** (R_f = 0.6, Hexane/AcOEt 8:1, 0.02 mmol, 10 %) and 23 mg of **18** (R_f = 0.2, Hexane/AcOEt 2:1, 0.08 mmol, 44 %).

***Synthesis of (3R,4R,5R)-4,5-O-(1-methylethylidene)-3-acetyl-N-Boc-piperidine* (40).**

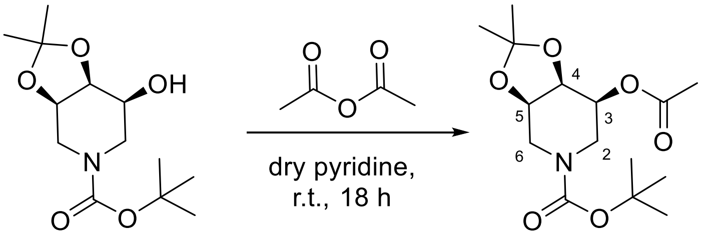

18 **40**

A solution of **18** (23 mg, 0.08 mmol) in dry pyridine (0.3 mL) was stirred with acetic anhydride (0.2 mL, 2.12 mmol) at room temperature for 18 h. The crude mixture was diluted with toluene and then concentrated under vacuum. The crude was purified by gradient silica gel column chromatography (Hexane/AcOEt 5:1) to give 9.5 mg of the acetylated compound **40** (R_f = 0.2, Hexane/AcOEt 5:1, 0.04 mmol, 56 %) as a pale-yellow oil.

40: $[\alpha]_D^{25}$ = + 9.92 (c = 1.20, $CHCl_3$). ^{1}H-NMR (400 MHz, $CDCl_3$) δ ppm: 5.09-4.99 (m, 1H, H-3), 4.51-4.43 (m, 1H, H-4), 4.36 (br s, 2H, H-5), 3.75-3.60 (m, 1H, Ha-6), 3.58-3.49 (m, 1H, Ha-2), 3.42 (d, J= 10.8 Hz, 1H, Hb-2), 3.35 (dd, J= 14.4, 3.7 Hz, 1H, Hb-6), 2.13 (s, 3H, CH_3COO), 1.49 (s, 3H, Me), 1.45 (s, 9H, t-Bu), 1.35 (s, 3H, Me). ^{13}C-NMR (50 MHz, $CDCl_3$) δ ppm: 170.4 (s, 1C, $CH_3\underline{C}OO$), 155.1 (s, 1C, N$\underline{C}$OO), 110.0 (s, 1C, O$\underline{C}(CH_3)_2$, 80.3 (s, 1C, O$\underline{C}(CH_3)_3$, 72.9 (d, 1C, C-5), 72.3 (d, 1C, C-4), 67.5 (s, 1C, C-3), 42.5 (t, 1C, C-6), 41.1 (t, 1C, C-2), 28.5 (q, 3C, OC($\underline{C}H_3)_3$), 26.8 (q, 1C, OC($\underline{C}H_3)_2$), 25.0 (q, 1C, OC($\underline{C}H_3)_2$), 21.2 (q, 1C, $\underline{C}H_3COO$). IR ($CDCl_3$) ν = 3686, 2967, 2930, 1734, 1690, 1416, 137, 1254, 1163, 1082 cm^{-1}. $C_{10}H_{17}NO_4$ (215.25): calcd. C, 55.80; H, 7.96; N, 6.51; found C, 55.93; H, 7.80; N, 6.66. MS-ESI (m/z, %) = 652.87 (100) $[2M+Na]^+$, 338.06 (70) $[M+Na]^+$.

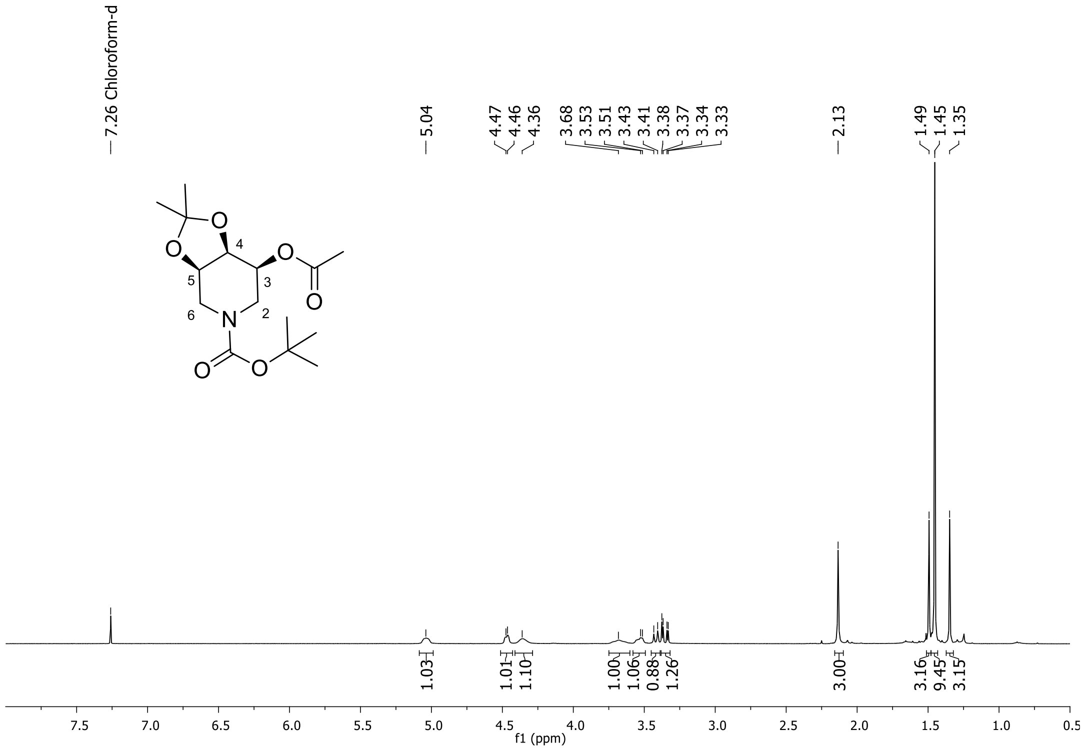

Figure S13: ^{1}H NMR spectrum of 40 (400 MHz, $CDCl_3$)

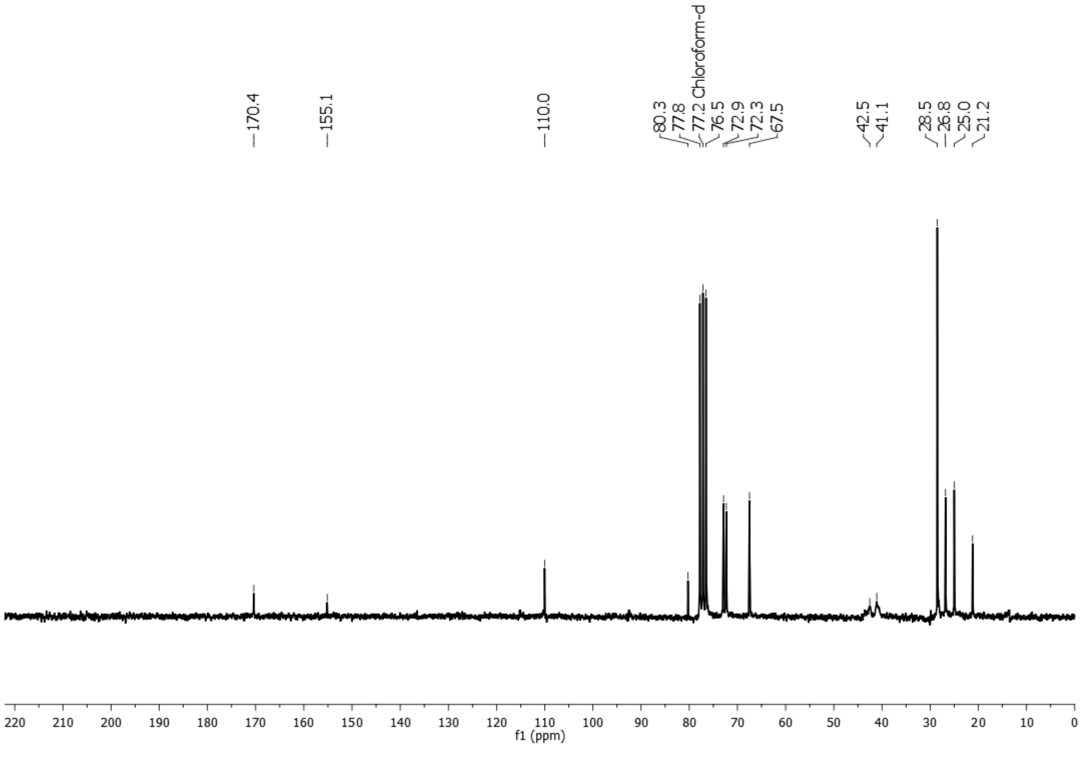

Figure S14: ^{13}C NMR spectrum of 40 (50 MHz, $CDCl_3$)

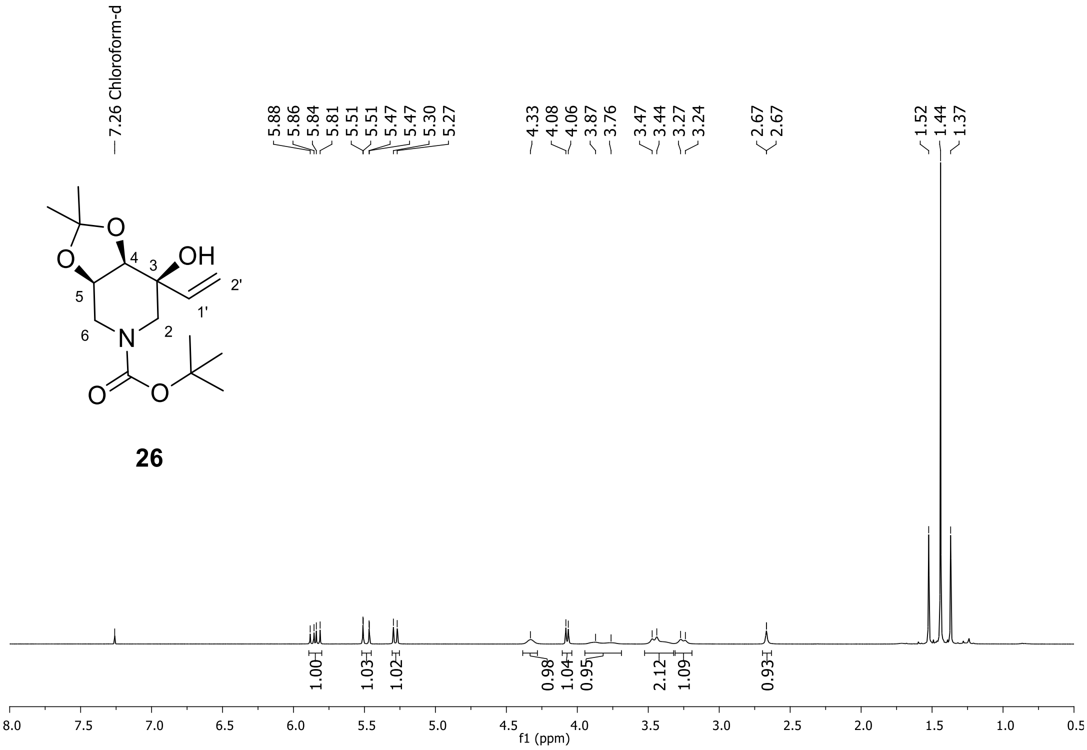

Figure S15: ^{1}H NMR spectrum of 26 (400 MHz, $CDCl_3$)

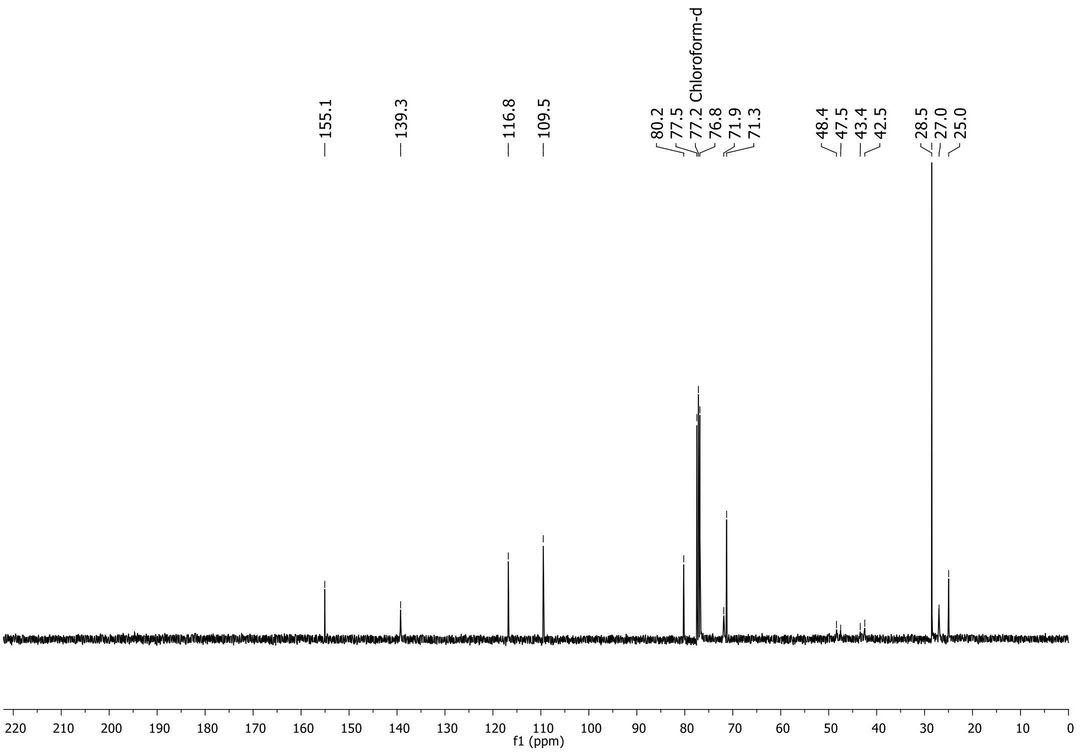

Figure S16: ^{13}C NMR spectrum of 26 (100 MHz, $CDCl_3$)

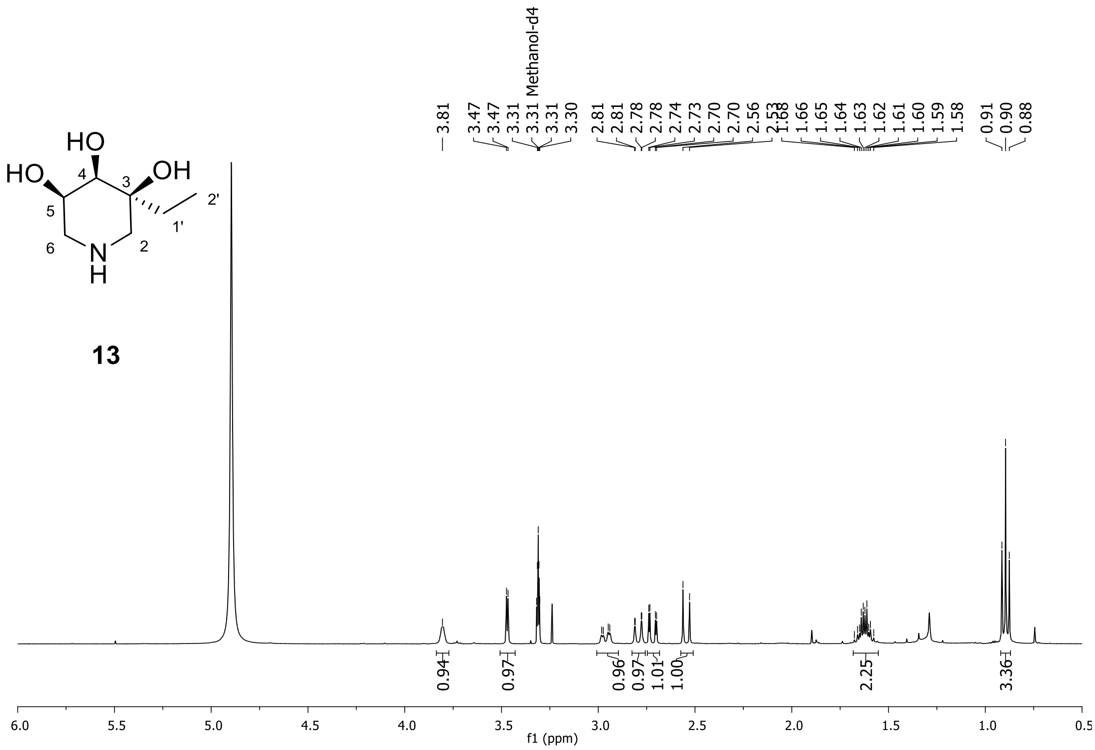

Figure S17: ^{1}H NMR spectrum of 13 (400 MHz, CD_3OD)

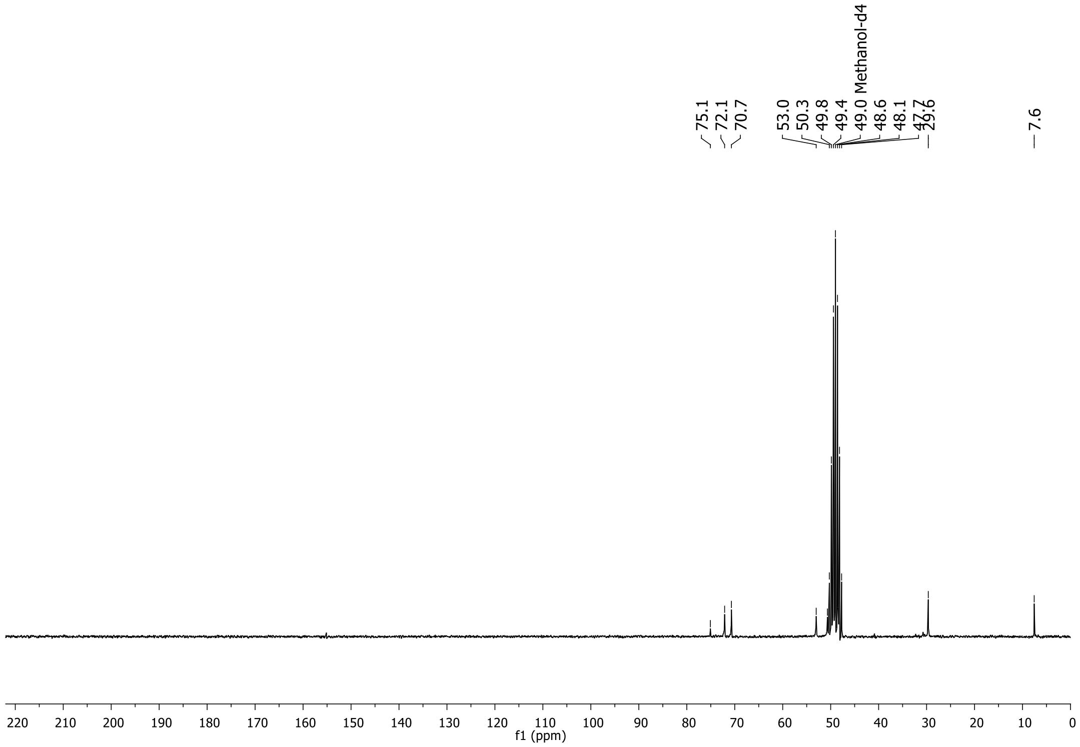

Figure S18: ^{13}C NMR spectrum of 13 (50 MHz, CD_3OD)

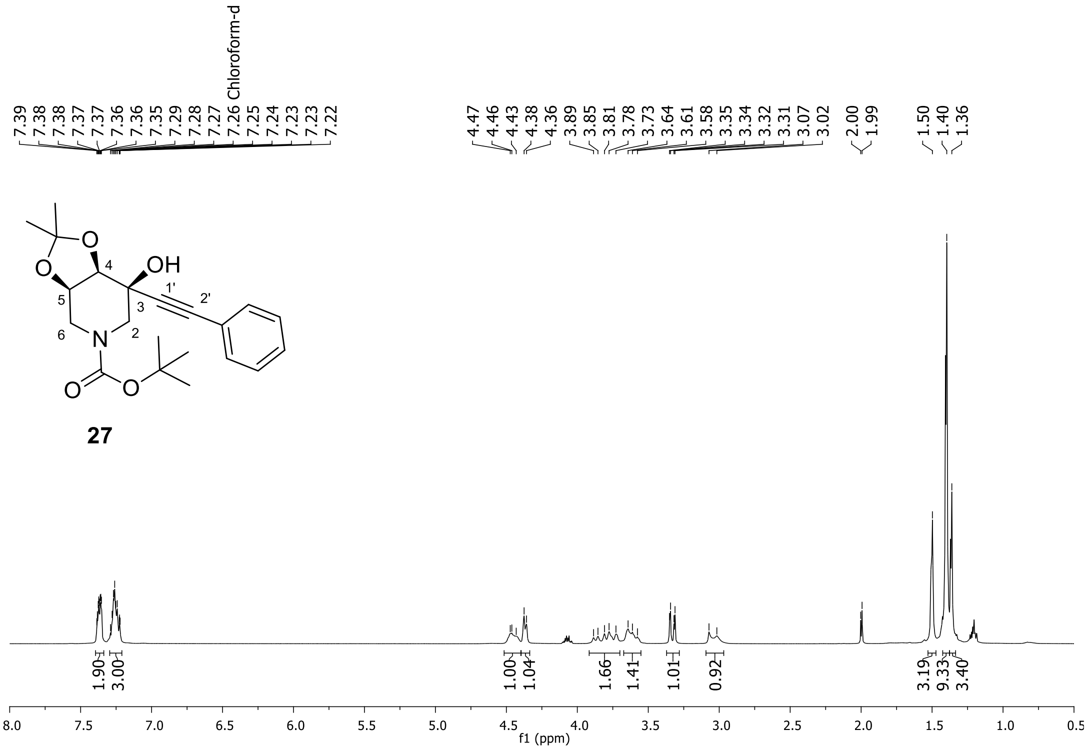

Figure S19: ^{1}H NMR spectrum of 27 (400 MHz, $CDCl_3$)

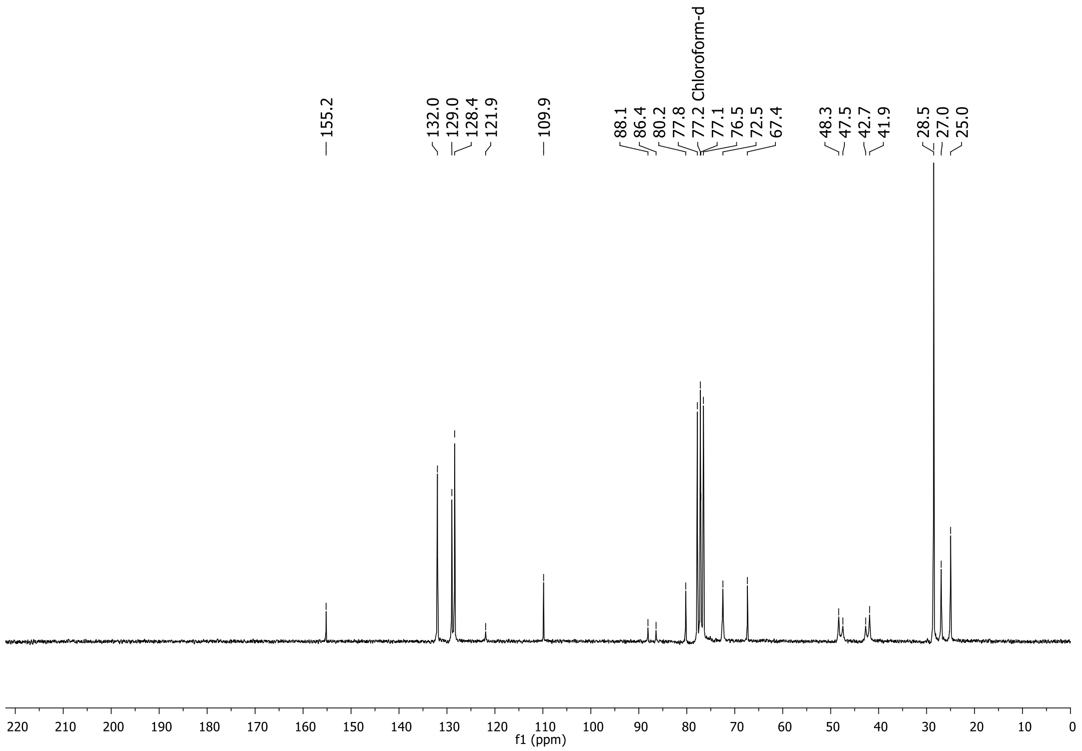

Figure S20: ^{13}C NMR spectrum of 27 (50 MHz, $CDCl_3$)

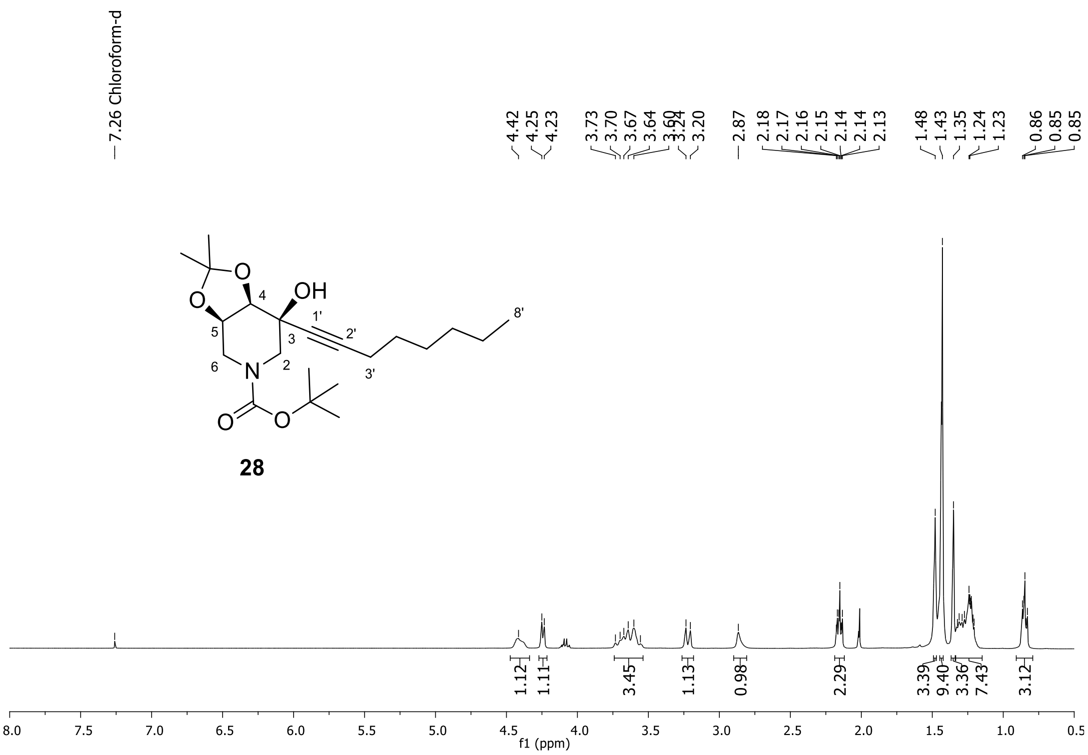

Figure S21: ^{1}H NMR spectrum of 28 (400 MHz, $CDCl_3$)

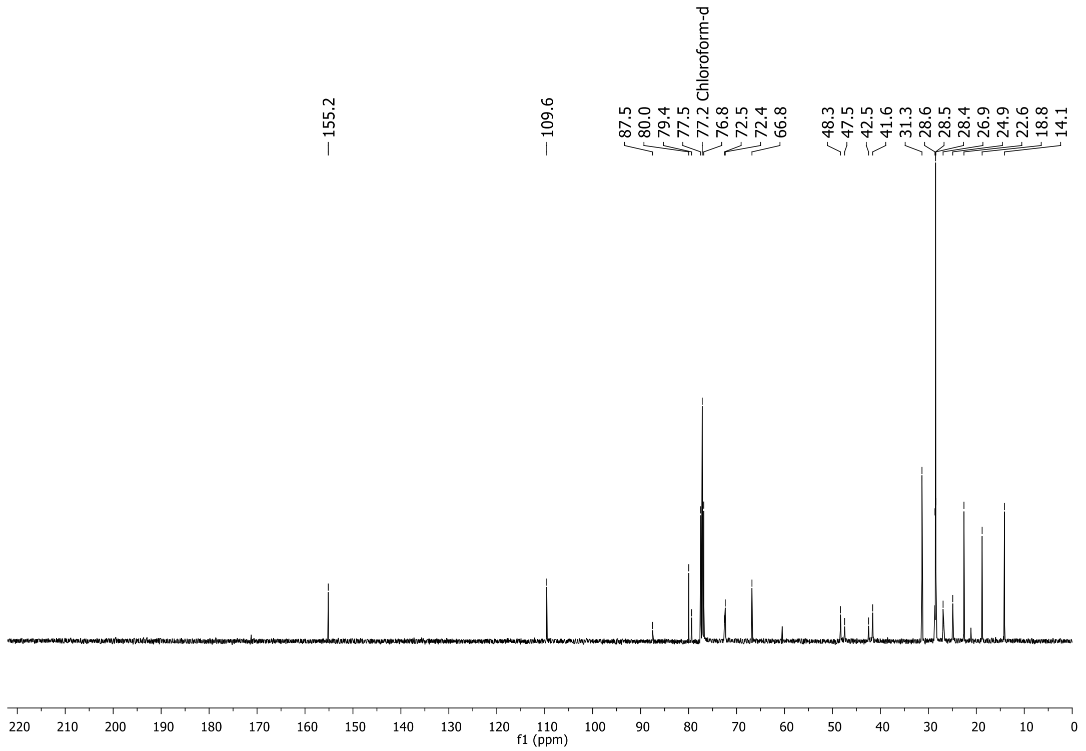

Figure S22: ^{13}C NMR spectrum of 28 (100 MHz, $CDCl_3$)

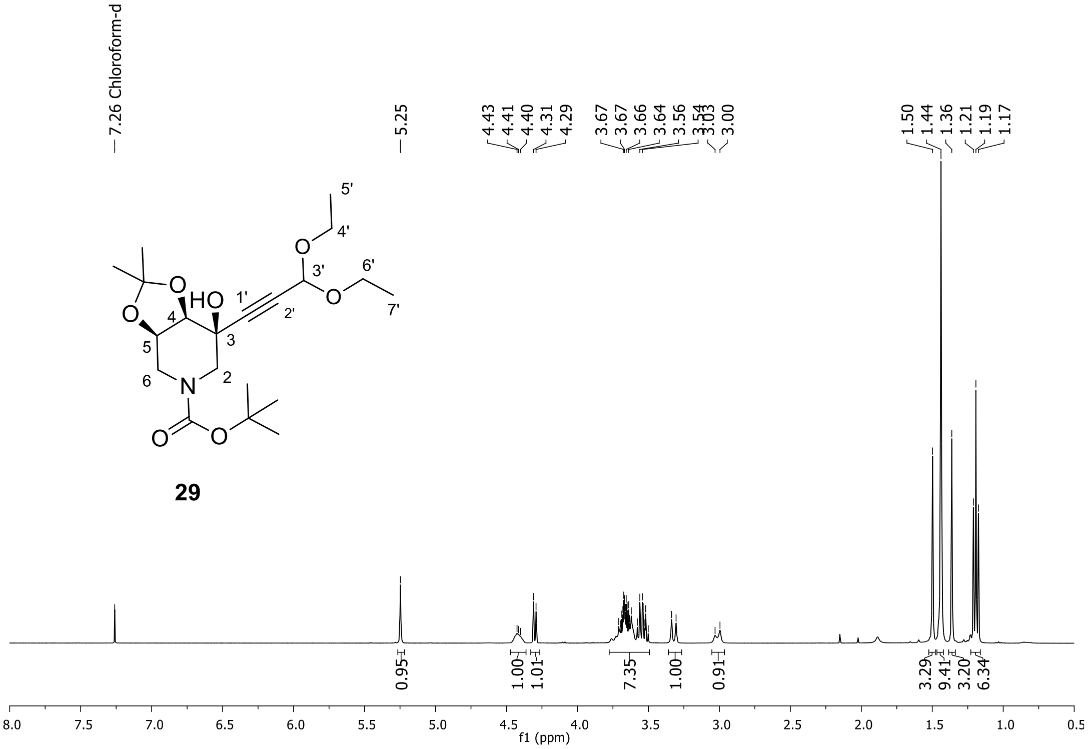

Figure S23: ^{1}H NMR spectrum of 29 (400 MHz, $CDCl_3$)

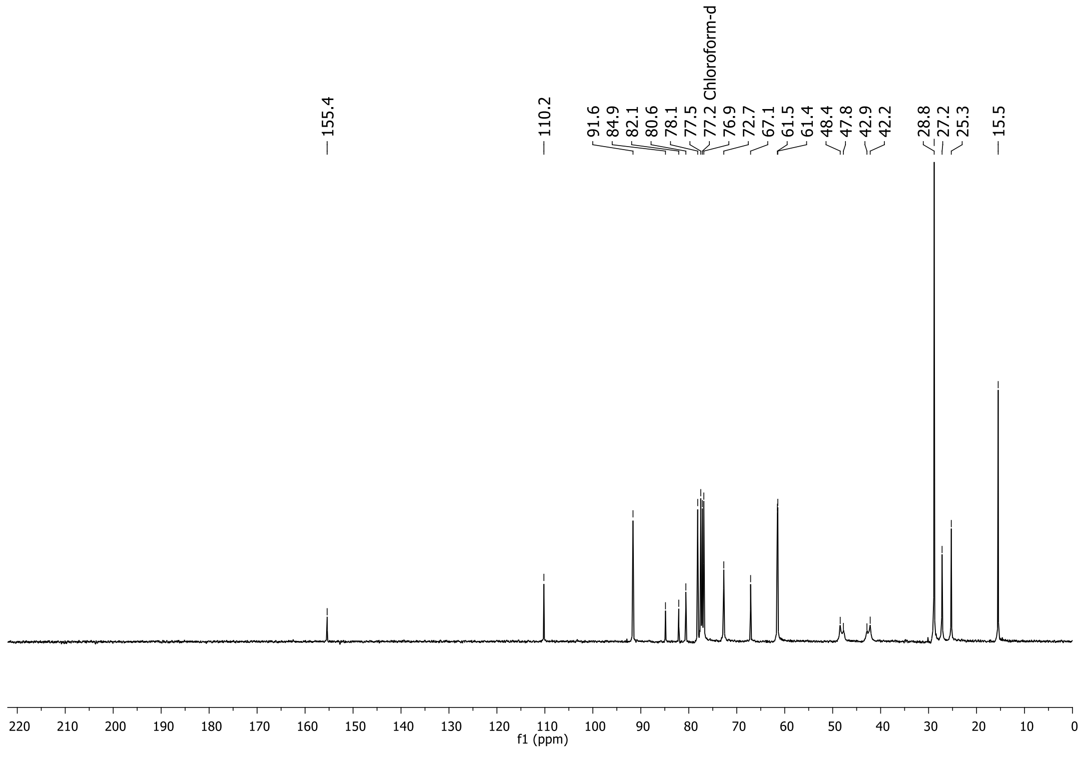

Figure S24: ^{13}C NMR spectrum of 29 (50 MHz, $CDCl_3$)

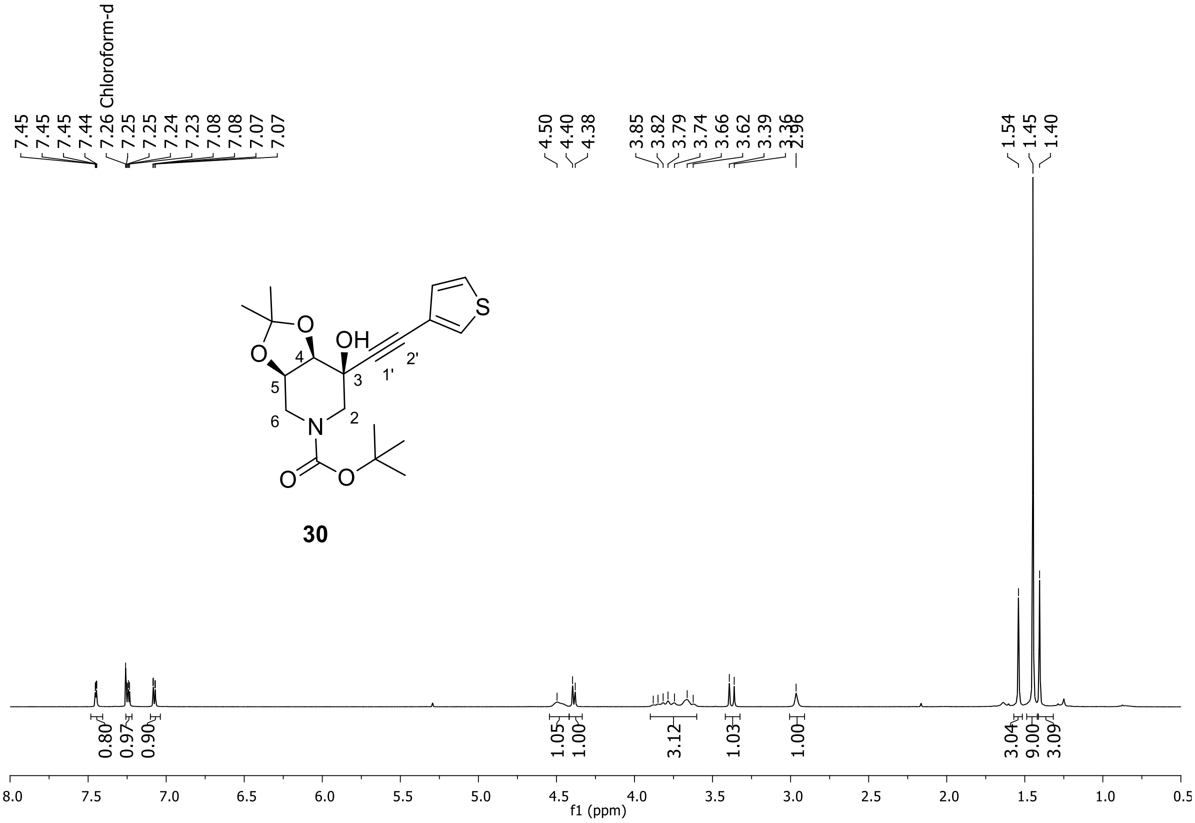

Figure S25: ^{1}H NMR spectrum of 30 (400 MHz, CDCl$_3$)

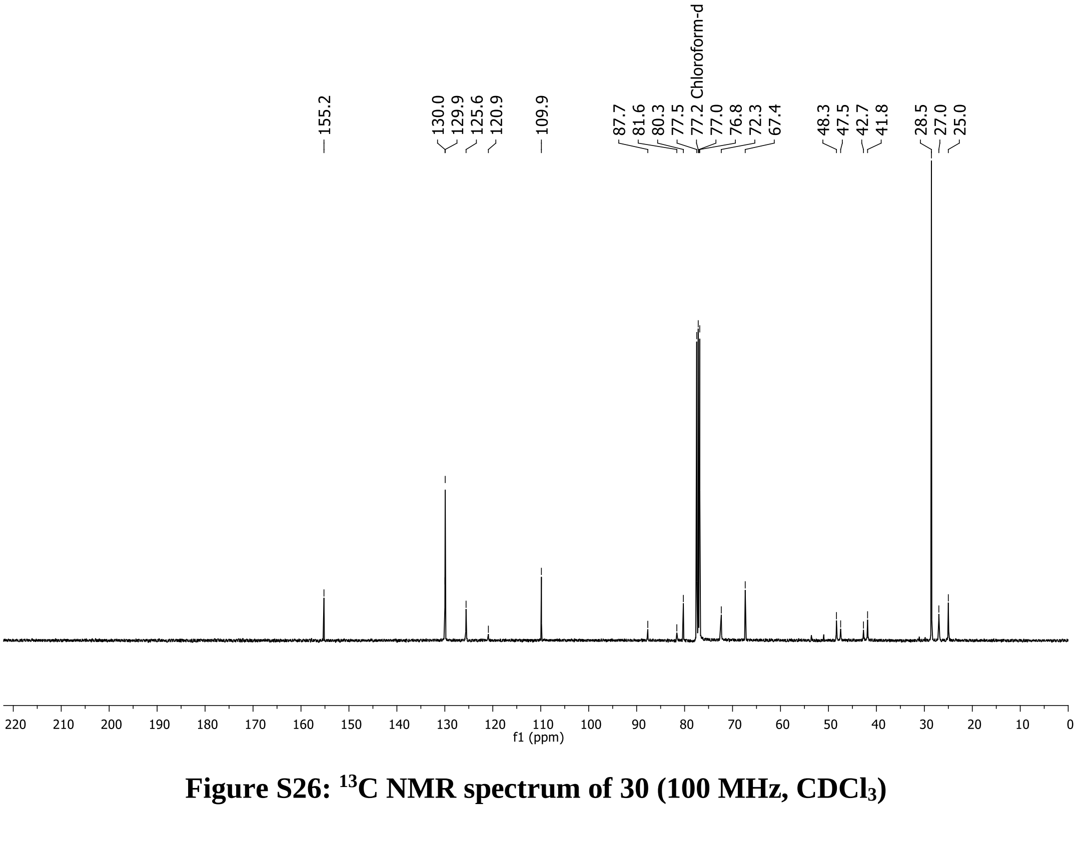

Figure S26: ^{13}C NMR spectrum of 30 (100 MHz, CDCl$_3$)

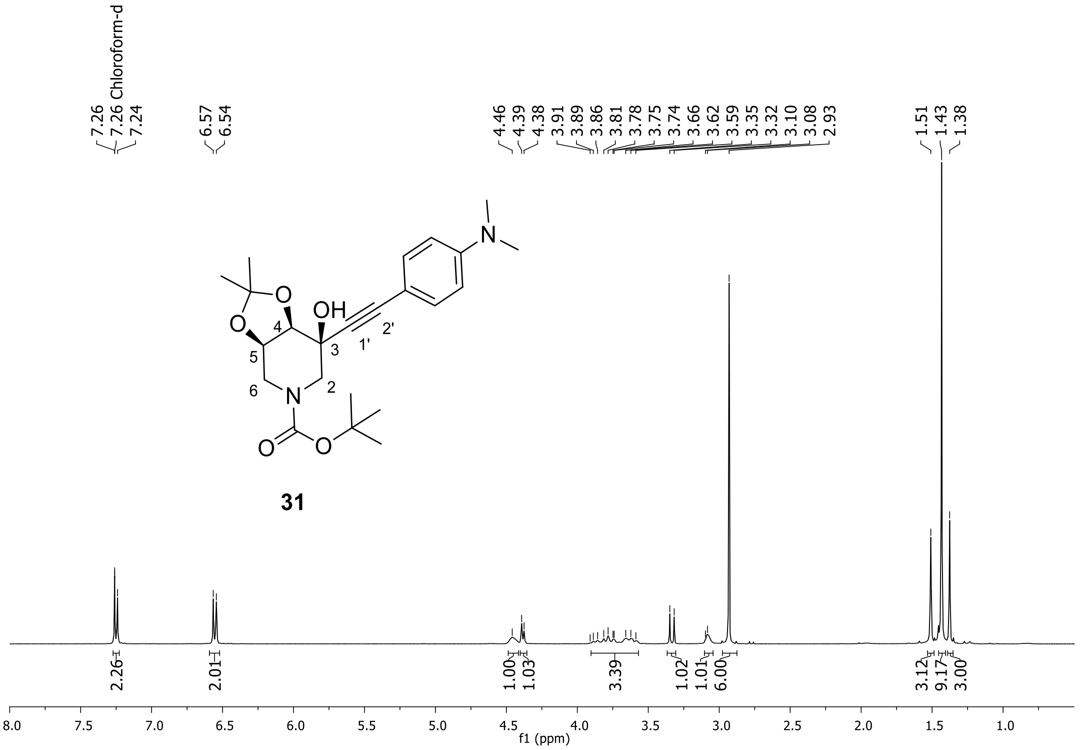

Figure S27: ^{1}H NMR spectrum of 31 (400 MHz, $CDCl_3$)

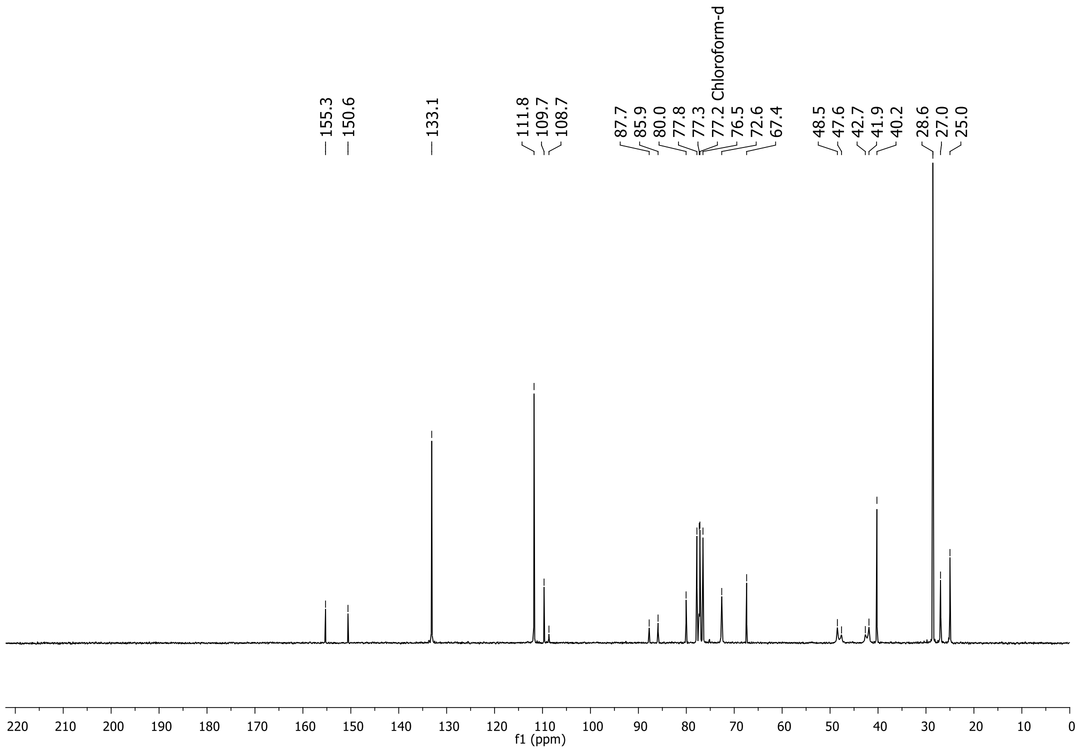

Figure S28: ^{13}C NMR spectrum of 31 (50 MHz, $CDCl_3$)

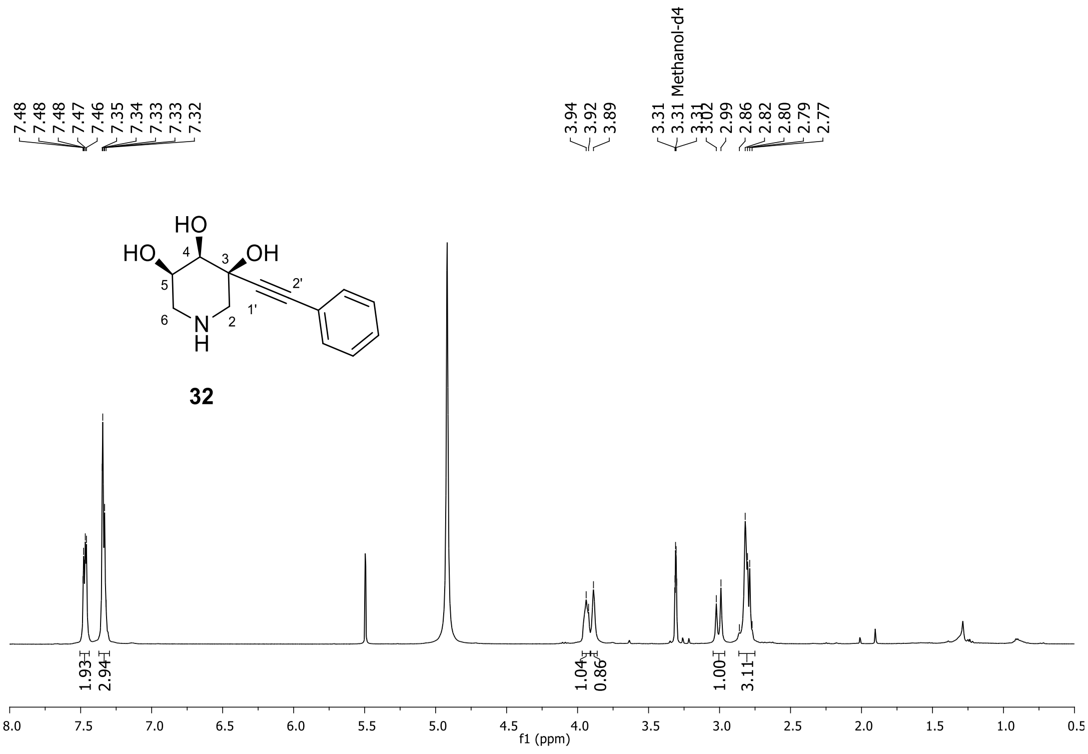

Figure S29: ^{1}H NMR spectrum of 32 (400 MHz, CD_3OD)

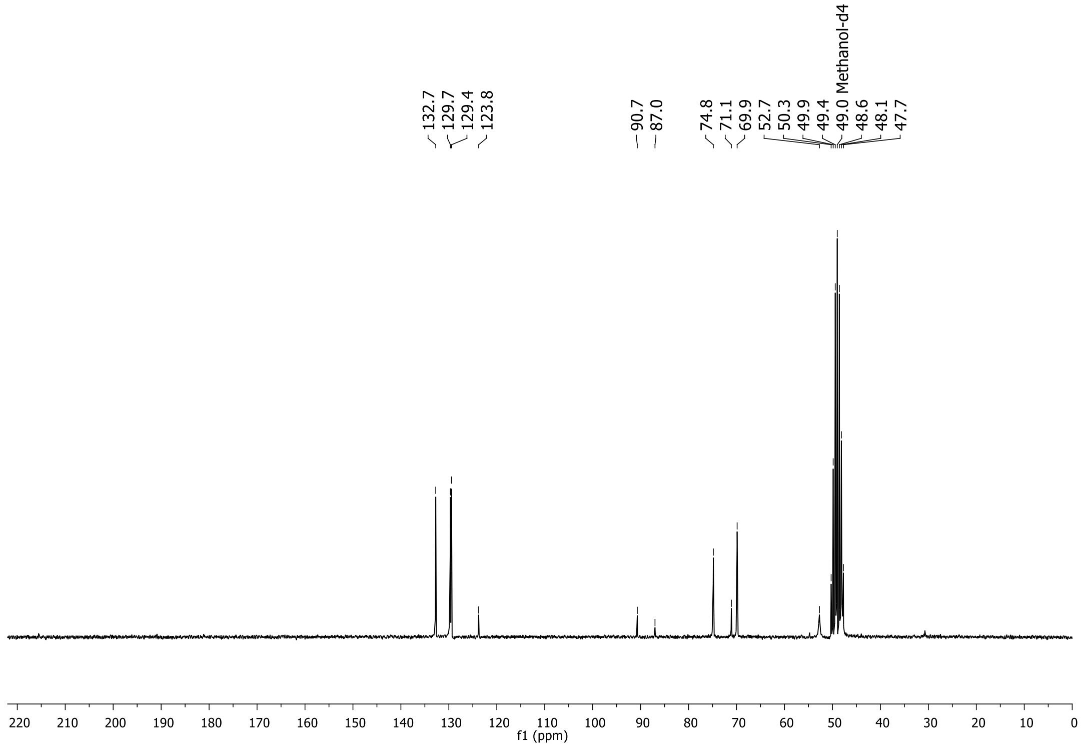

Figure S30: ^{13}C NMR spectrum of 32 (50 MHz, CD_3OD)

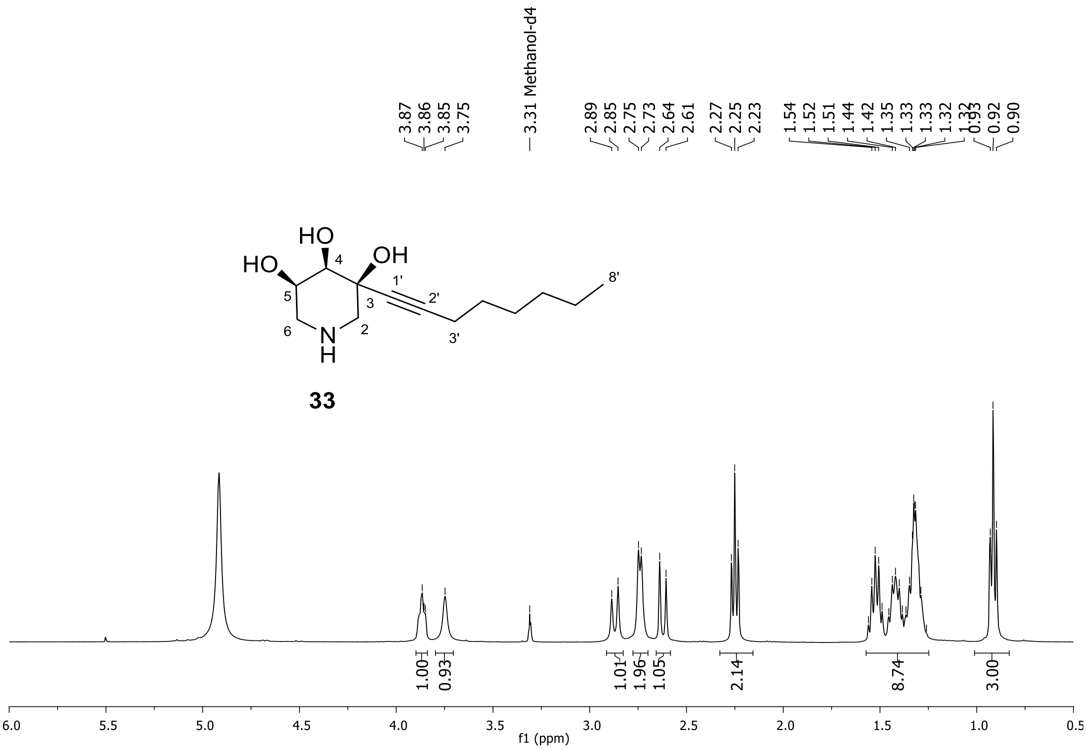

Figure S31: ¹H NMR spectrum of 33 (400 MHz, CD_3OD)

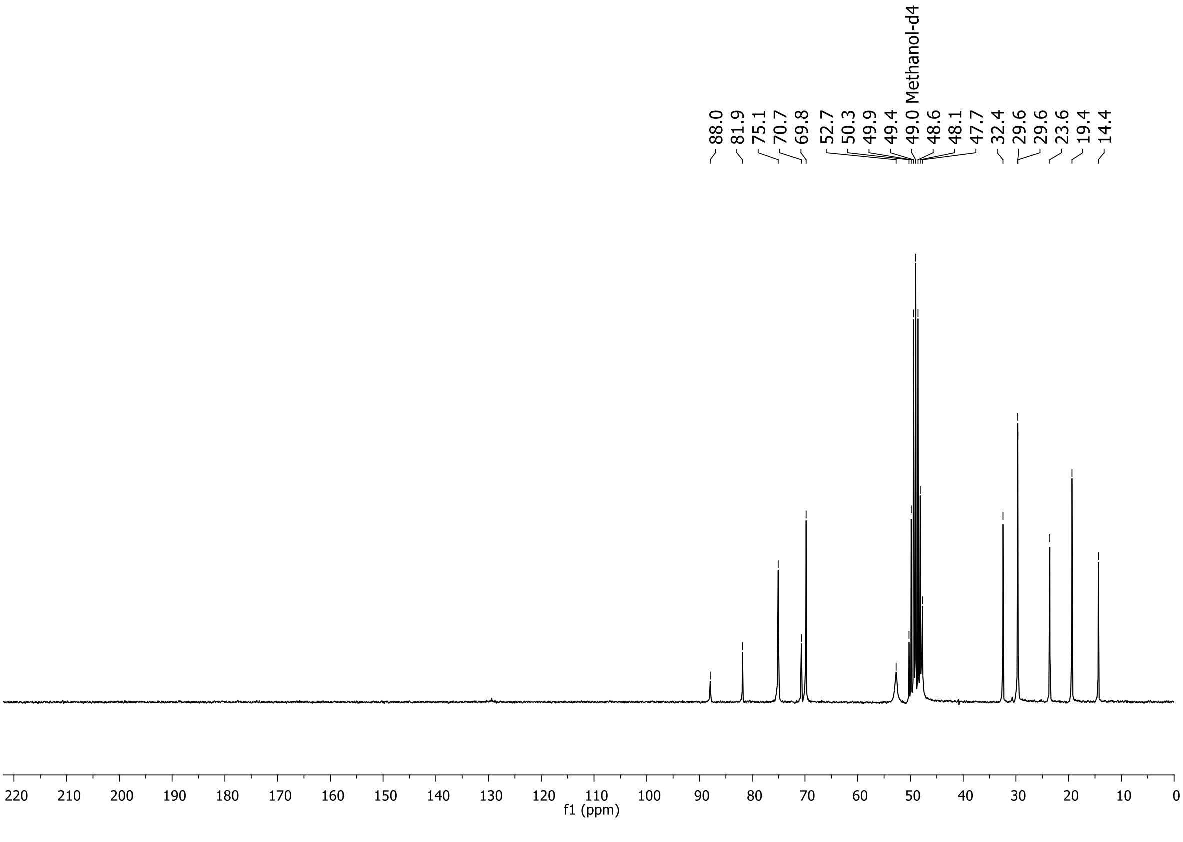

Figure S32: ¹³C NMR spectrum of 33 (50 MHz, CD_3OD)

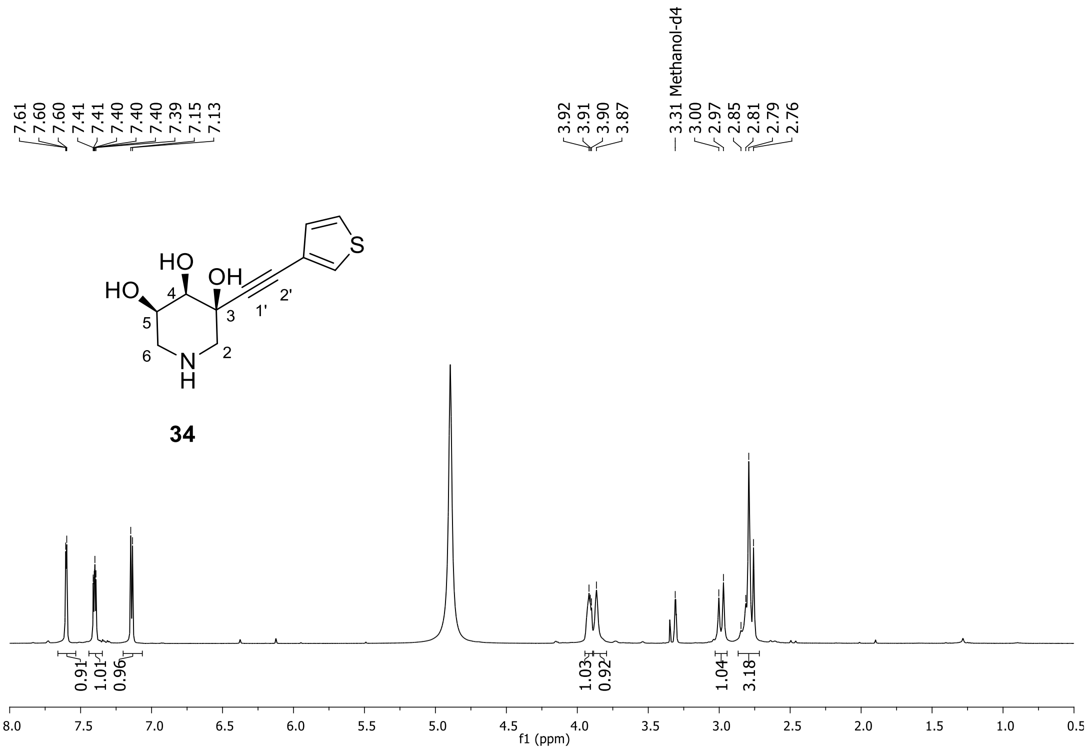

Figure S33: ^{1}H NMR spectrum of 34 (400 MHz, CD_3OD)

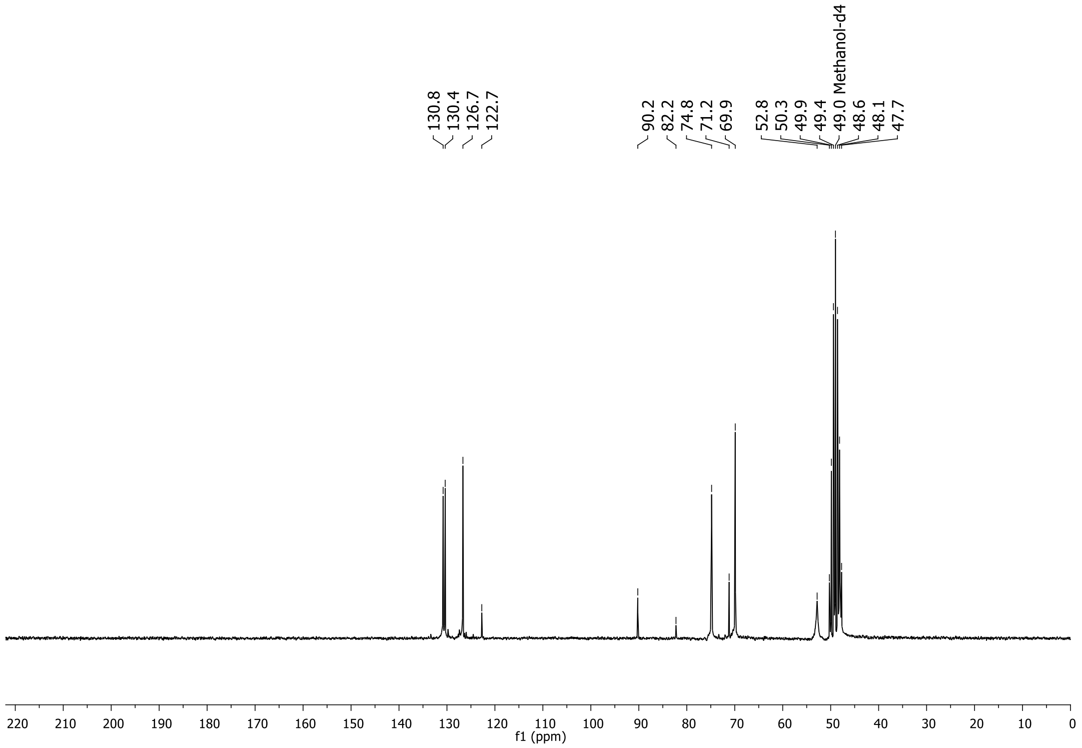

Figure S34: ^{13}C NMR spectrum of 34 (50 MHz, CD_3OD)

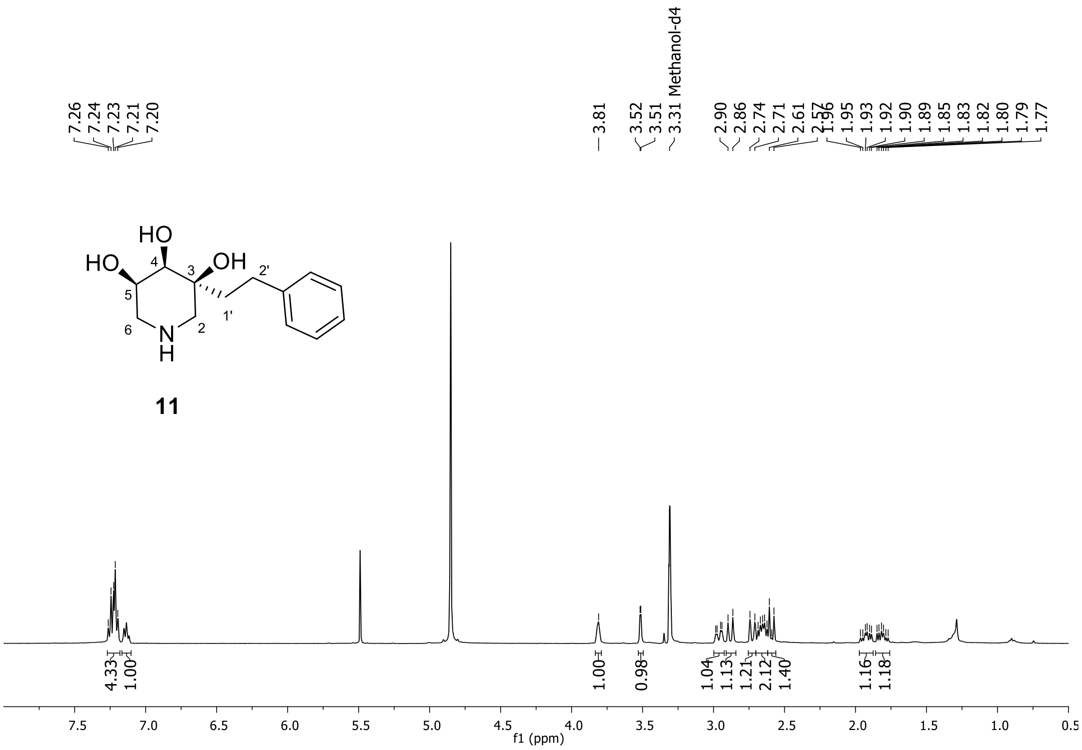

Figure S35: ^{1}H NMR spectrum of 11 (400 MHz, CD_3OD)

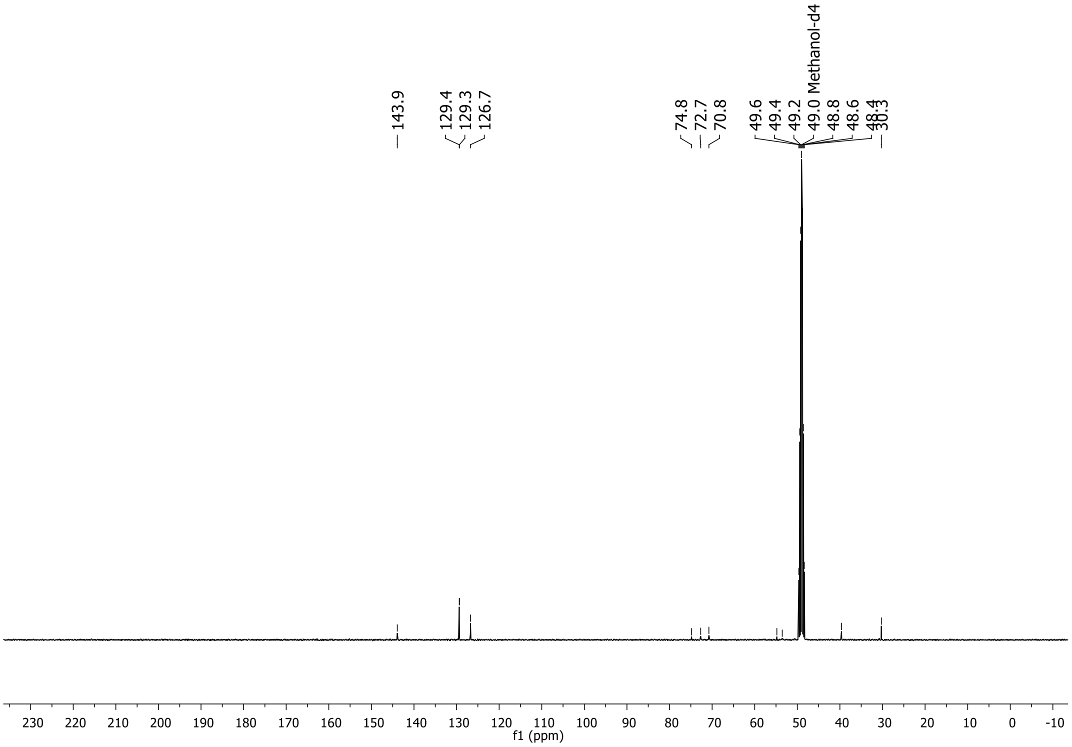

Figure S36: ^{13}C NMR spectrum of 11 (100 MHz, CD_3OD)

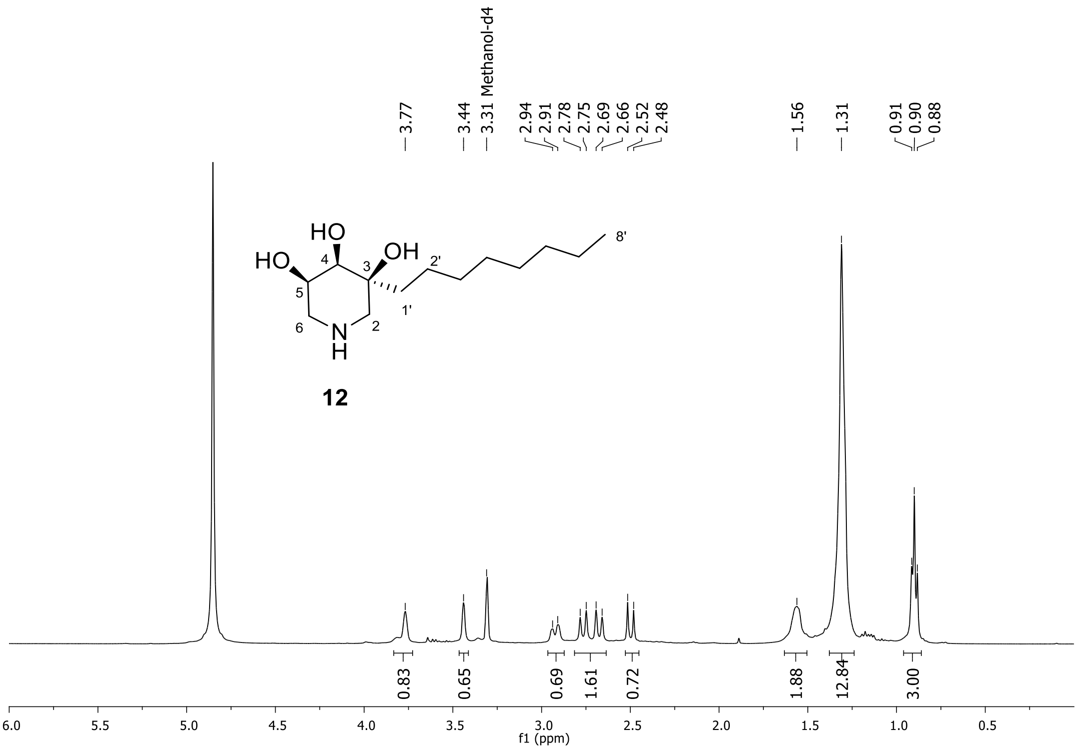

Figure S37: ^{1}H NMR spectrum of 12 (400 MHz, CD_3OD)

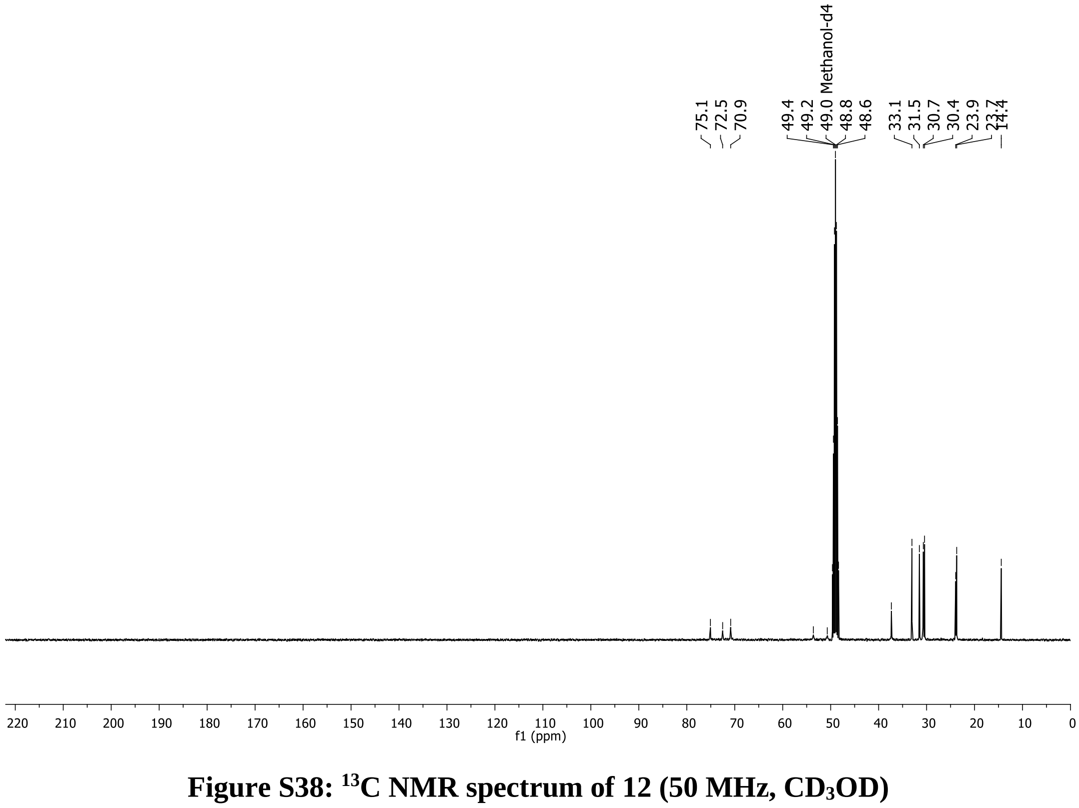

Figure S38: ^{13}C NMR spectrum of 12 (50 MHz, CD_3OD)

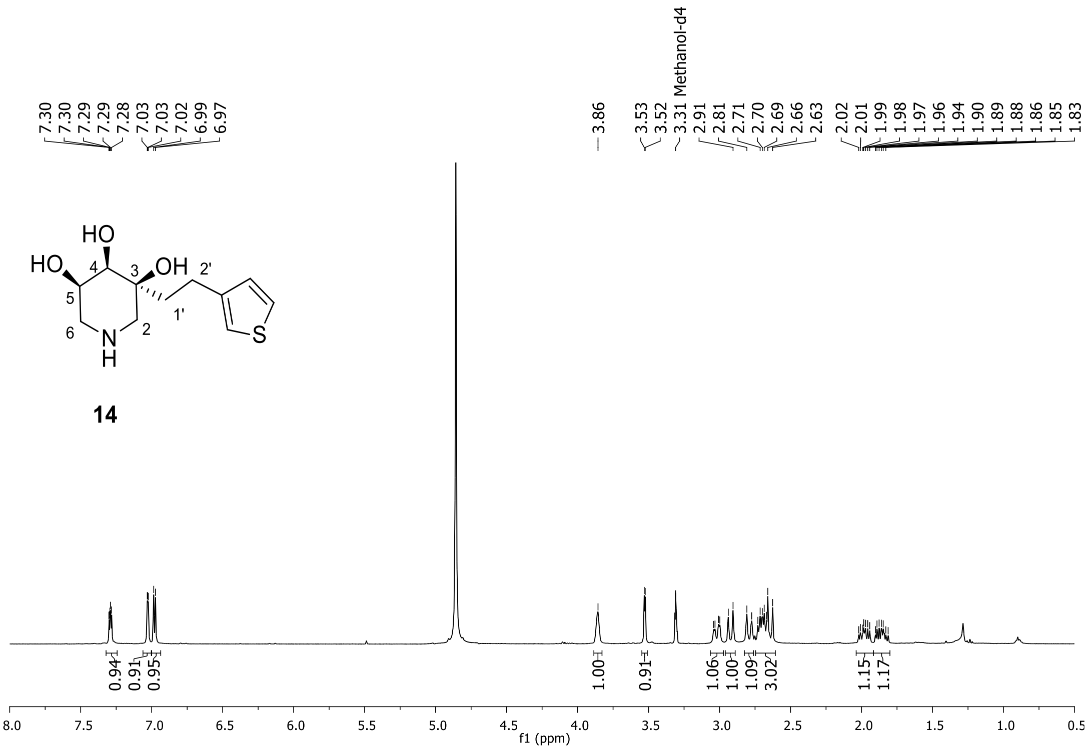

Figure S39: ^{1}H NMR spectrum of 14 (400 MHz, CD_3OD)

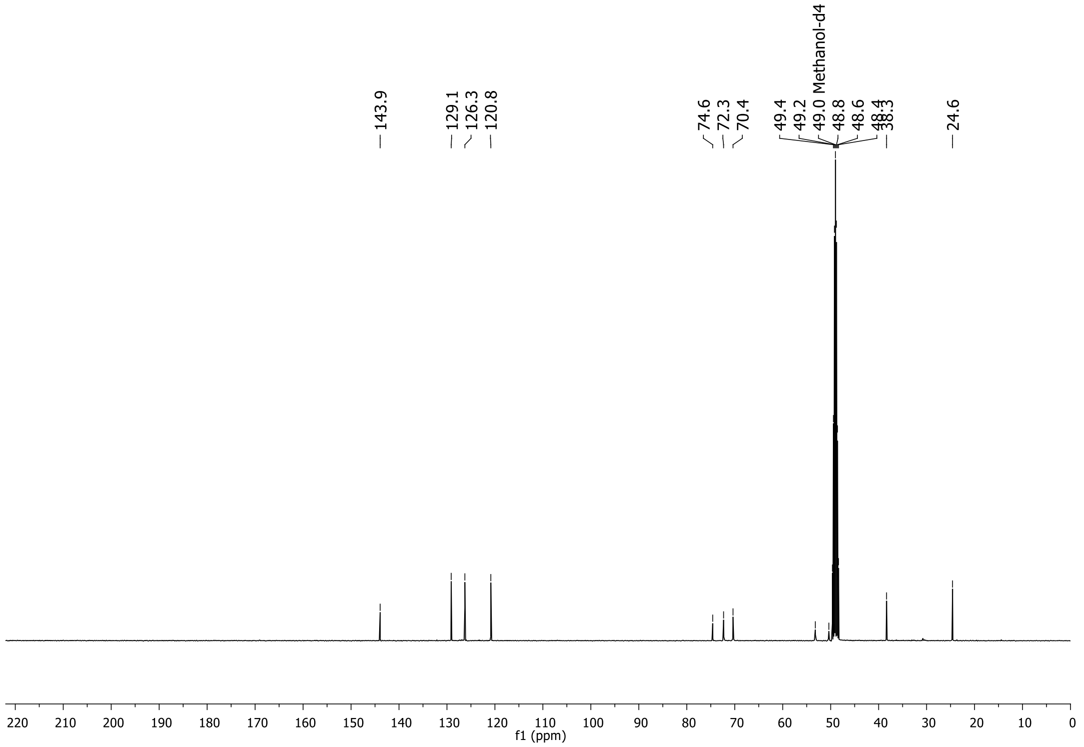

Figure S40: ^{13}C NMR spectrum of 14 (100 MHz, CD_3OD)

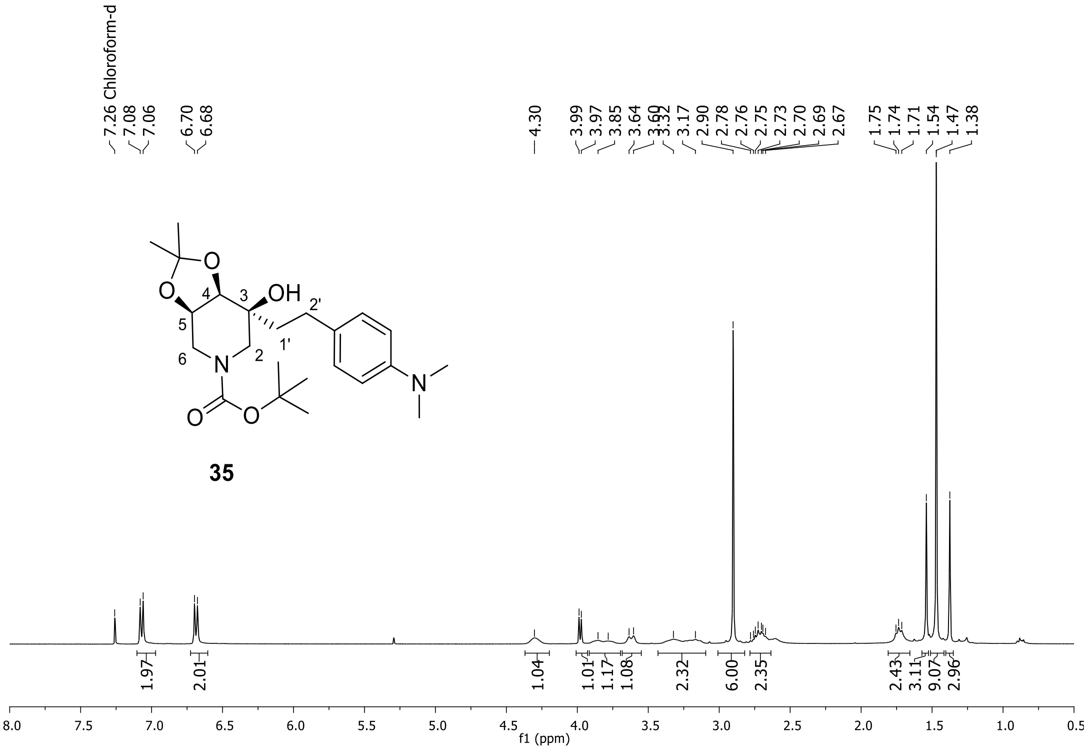

Figure S41: ^{1}H NMR spectrum of 35 (400 MHz, $CDCl_3$)

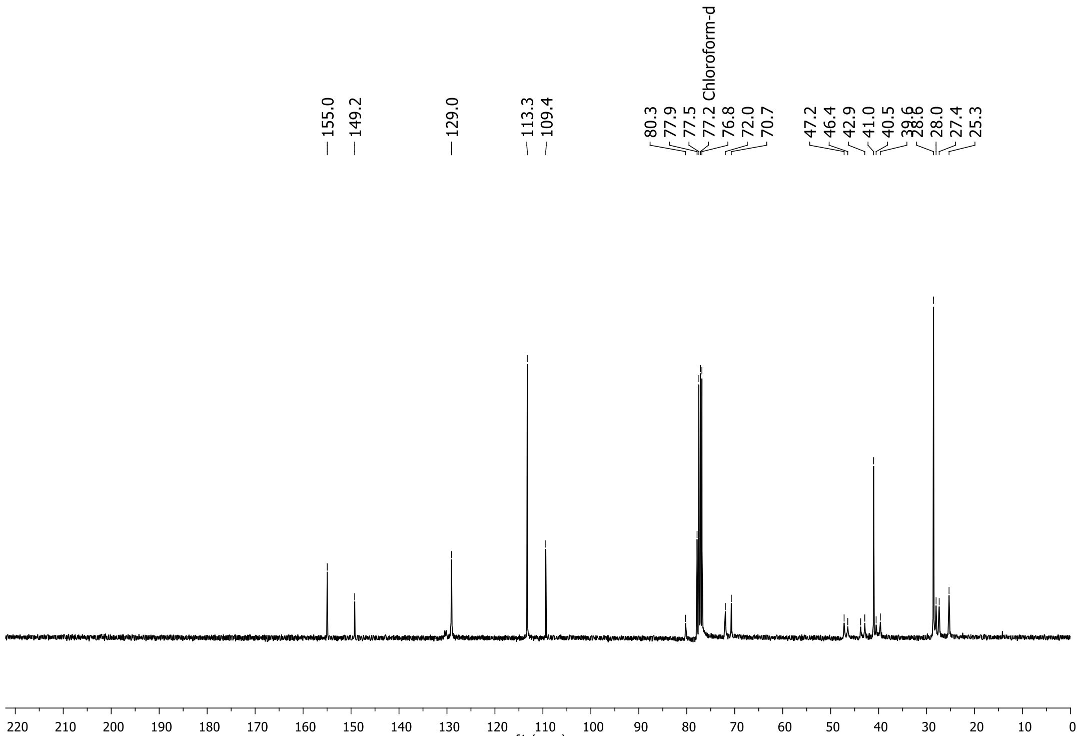

Figure S42: ^{13}C NMR spectrum of 35 (100 MHz, $CDCl_3$)

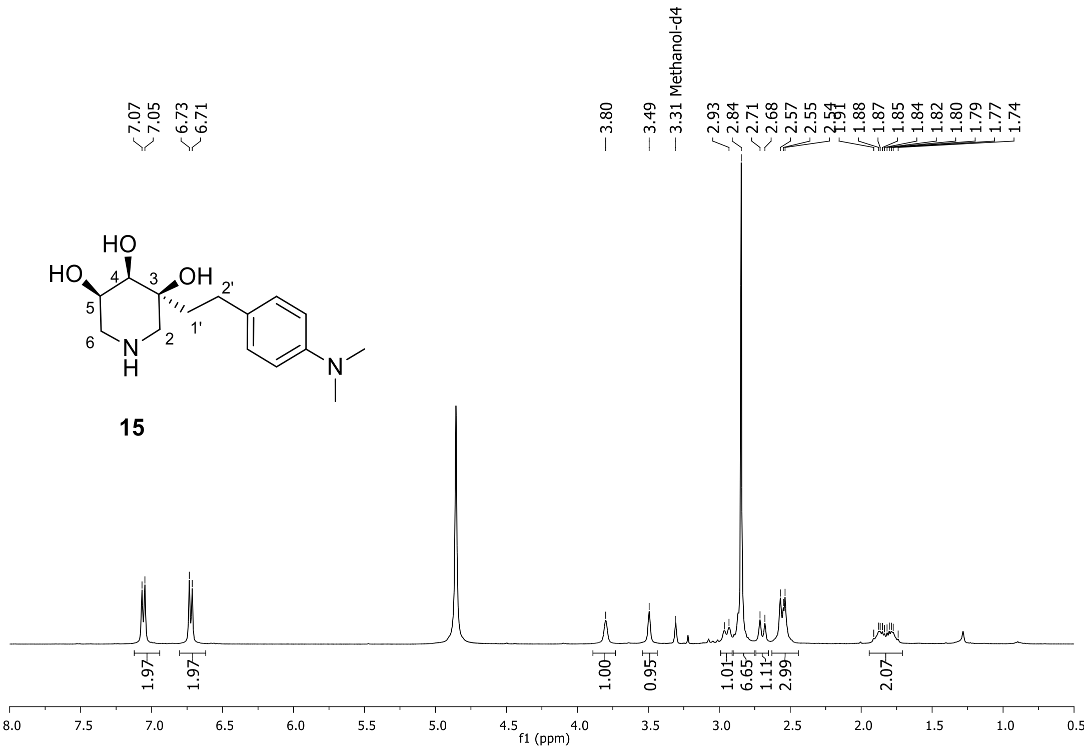

Figure S43: ^{1}H NMR spectrum of 15 (400 MHz, CD_3OD)

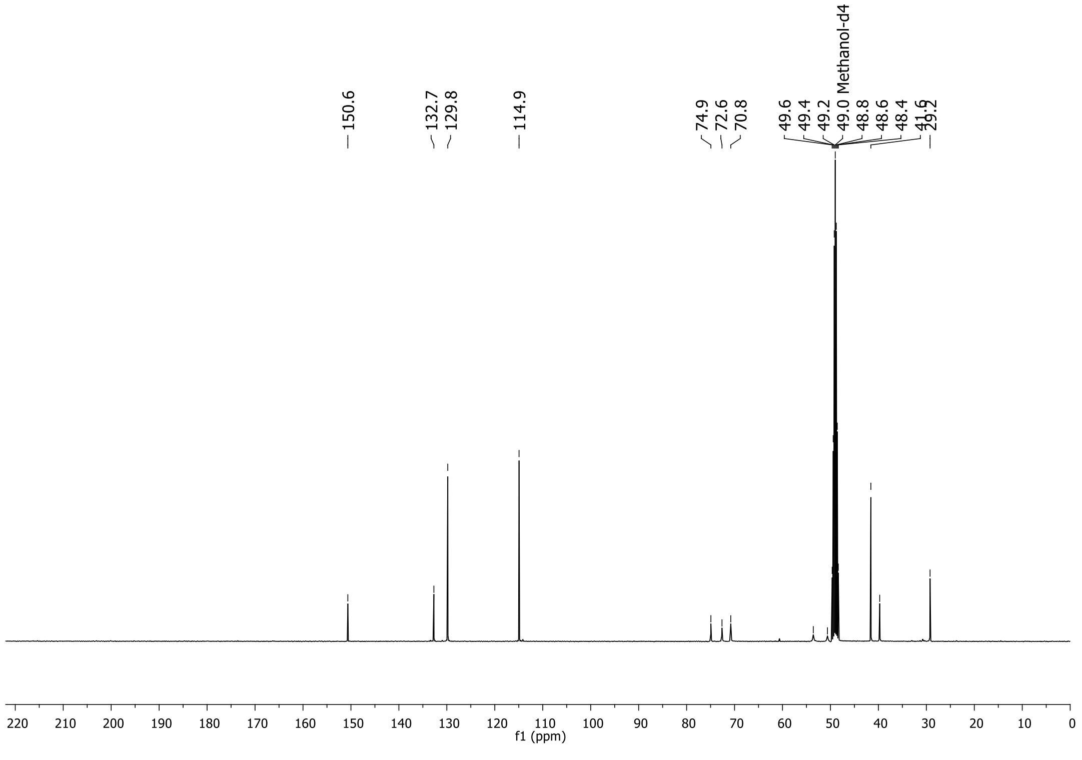

Figure S44: ^{13}C NMR spectrum of 15 (100 MHz, CD_3OD)

Configuration assignment

Relevant chemical shifts and coupling constants are reported in Tables S2a and S2b for H-4, H-5 and H-6 in ^{1}H NMR spectra in the two series of protected (in $CDCl_3$) and deprotected (in CD_3OD) trihydroxypiperidines, respectively. These values show regularities (the same applies to H-2 signals) which allowed us to ascribe the same configuration at C-3 for all compounds. Moreover, the shape of the signals and their coupling constants, where detectable, are consistent with the (*S*) absolute configuration tentatively assigned (see above) on the basis of mechanistic considerations. Indeed, the signal of H-5 appears as a broad singlet (or as a narrow multiplet), which is in agreement with its equatorial position in a preferred chair conformation which places the R substituent equatorially, *ie*, in the (3*S*) configuration ($_6C^3$ alcohol in Scheme 5 of the main text). The lack of large *ax-ax* coupling constants is confirmed by signals of H-6. For example, in piperidine **13** (R = ethyl) the two hydrogens at C-6 display vicinal coupling constants J = 3.4 and J = 2.4 Hz, typical for *ax-eq* and *eq-eq* relationships. The same applies to the other derivatives when the signals are well resolved, as in compounds **11**, **12**, **14** and **15**. The observed upfield shift (0.3-0.5 ppm) of H-4 within the two series of compounds on turning from the alkynyl to the saturated substituents (see **27-31** *vs* **35** and **32-34** *vs* **11-15**), consistent with H-4 falling in the deshielding cone of the triple bond in the former derivatives when in a *cis* relationship, further supports this assignment.

Table S2a. Chemical shifts and coupling constants of H-4, H-5 and H-6 of protected compounds **26-31** and **35**, in $CDCl_3$.

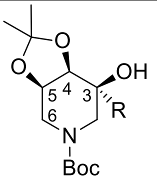	**H-4**<br>δ (ppm)	**H-5**<br>δ (ppm)	**H-6a**<br>δ (ppm)	**H-6b**<br>δ (ppm)
R = vinyl, **26**	4.07 (d, J= 6.8 Hz)	4.33 (br s)	3.95-3.69 (m)	3.53-3.32 (m)
R = 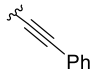, **27**	4.36 (d, J= 6.7 Hz)	4.52-4.40 (m)	3.92-3.70 (m)	3.67-3.55 (m)
R= 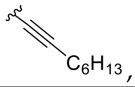, **28**	4.24 (d, J= 6.9 Hz)	4.47-4.34 (m)	3.74-3.54 (m)	
R= 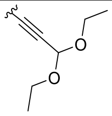, **29**	4.30 (d, J= 6.8 Hz)	4.47-4.36 (m)	3.78-3.49 (m)	
R= 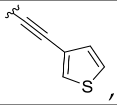, **30**	4.39 (d, J= 6.8 Hz)	4.55-4.42 (m)	3.90-3.60 (m)	
R = 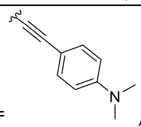, **31**	4.35 (d, J= 6.8 Hz)	4.49-4.42 (m)	3.90-3.57 (m)	
R = 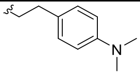, **35**	3.98 (d, J= 6.4 Hz)	4.30 (br s)	3.68-3.55 (m)	3.43-3.10 (m)

Table S2b. Chemical shifts and coupling constants of H-4, H-5 and H-6 of protected compounds **11-15** and **32-34**, in CD_3OD.

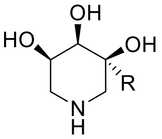	H-4<br>δ<br>(ppm)	H-5<br>δ<br>(ppm)	H-6a<br>δ<br>(ppm)	H-6b<br>δ<br>(ppm)
R = ethyl, **13**	3.47<br>(d, J= 3.2 Hz)	3.81<br>(br s)	2.96<br>(dd, J= 13.6, 3.4 Hz)	2.72<br>(dd, J= 13.7, 2.4 Hz)
R = 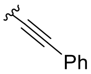, **32**	3.89<br>(br s)	3.98-3.91<br>(m)	2.86-2.76<br>(m)	
R = 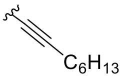, **33**	3.75<br>(br s)	3.90-3.84<br>(m)	2.78-2.70<br>(m)	
R = 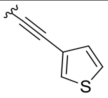, **34**	3.87<br>(br s)	3.95-3.89<br>(m)	2.85-2.74<br>(m)	
R = 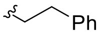, **11**	3.51<br>(br d, J= 2.2 Hz)	3.81<br>(br s)	2.96<br>(dd, J= 13.7, 4.0 Hz)	2.76-2.70<br>(m)
R = octyl, **12**	3.44<br>(br s)	3.77<br>(br s)	2.92<br>(d, J= 12.6 Hz)	2.68<br>(d, J= 12.6 Hz)
R = 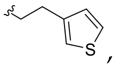, **14**	3.53<br>(br d, J= 2.7 Hz)	3.86<br>(br s)	3.02<br>(dd, J= 13.6, 3.8 Hz)	2.79<br>(br d, J= 13.6 Hz)
R = 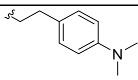, **15**	3.49<br>(br s)	3.80<br>(br s)	2.95<br>(d, J= 13.2 Hz)	2.70<br>(d, J= 13.8 Hz)

Table S3: Biological screening towards commercial glycosidases

	% Inhibition at 0.1 mM		
	**10**	**12**	**21**
**α-L-fucosidase EC 3.2.1.51**<br>Homo sapiens	-	-	-
**α-galactosidase EC 3.2.1.22**<br>coffee beans	-	-	-
**β-galactosidase EC 3.2.1.23**<br>*Escherichia coli*	-	-	-
Aspergillus oryzae	-	-	-
**α-glucosidase EC 3.2.1.20**<br>yeast	-	-	-
rice	-	-	-
**amyloglucosidase EC 3.2.1.3**<br>*Aspergillus niger*	-	-	-
**β-glucosidase EC 3.2.1.21**<br>almonds	51±1	-	-
**α-mannosidase EC 3.2.1.24**<br>Jack beans	-	-	-
**β-mannosidase EC 3.2.1.25**<br>snail	-	-	-
**β-*N*-acetylglucosaminidase EC 3.2.1.52**<br>Jack beans	-	-	-
bovine kidney	-	-	-

“-“: no inhibition detected.

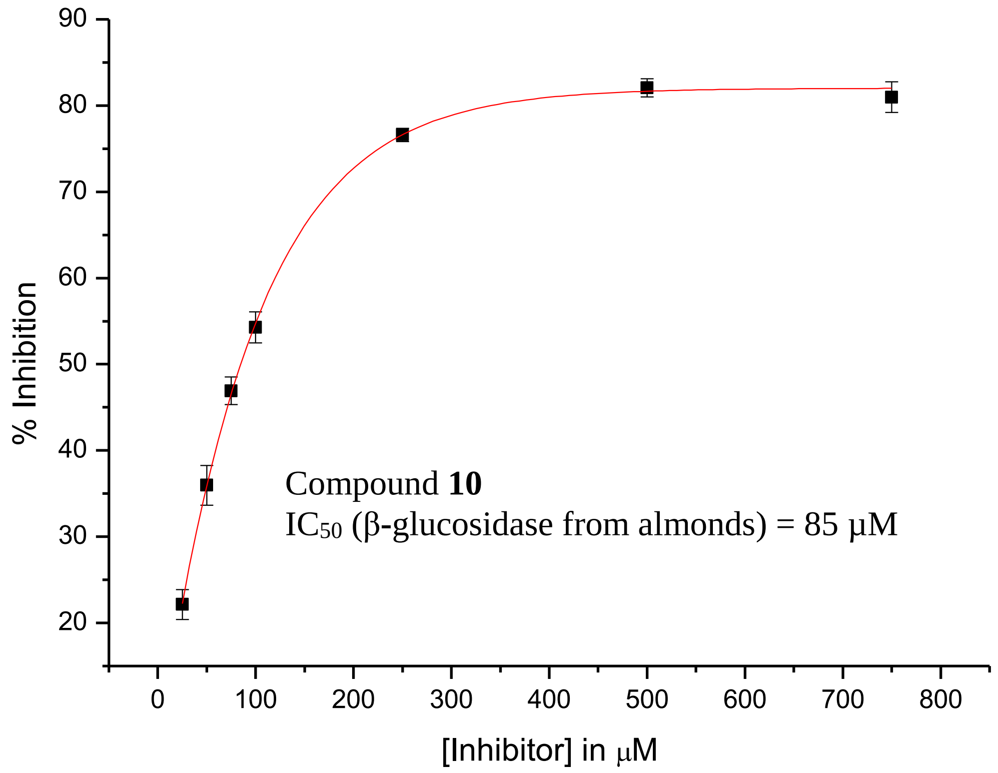

Figure S45: IC_{50} of compound 10 towards β-glucosidase from almonds

Biological screening towards human lysosomal β-galactosidase (β-Gal) and β-glucosidase (GCase)

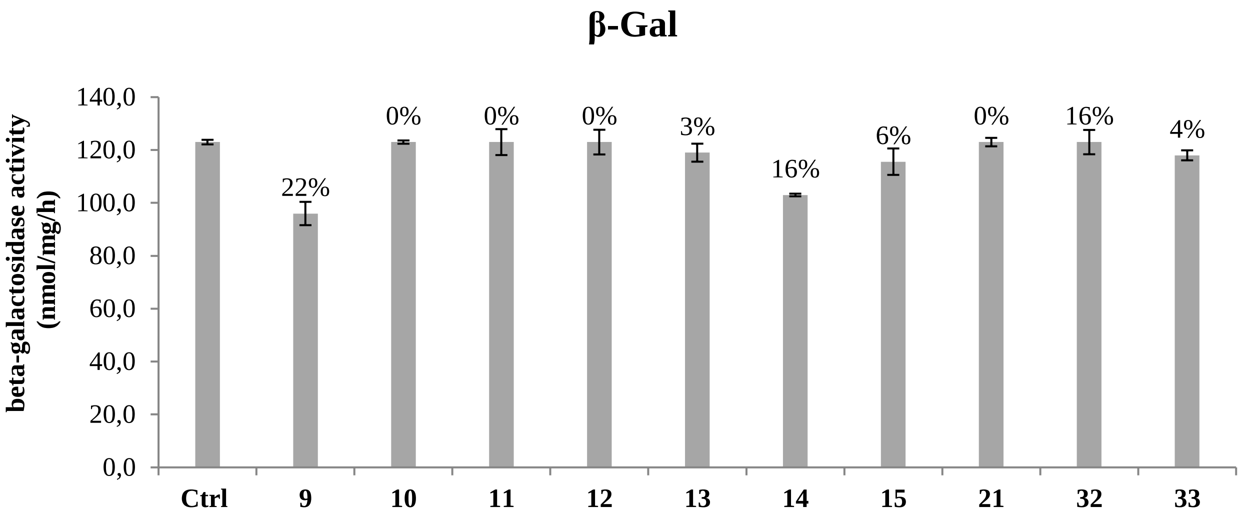

Figure S46: Percentage of β-Gal inhibition of the whole collection of compounds in human leukocytes extracts incubated with iminosugars at 1 mM concentration.

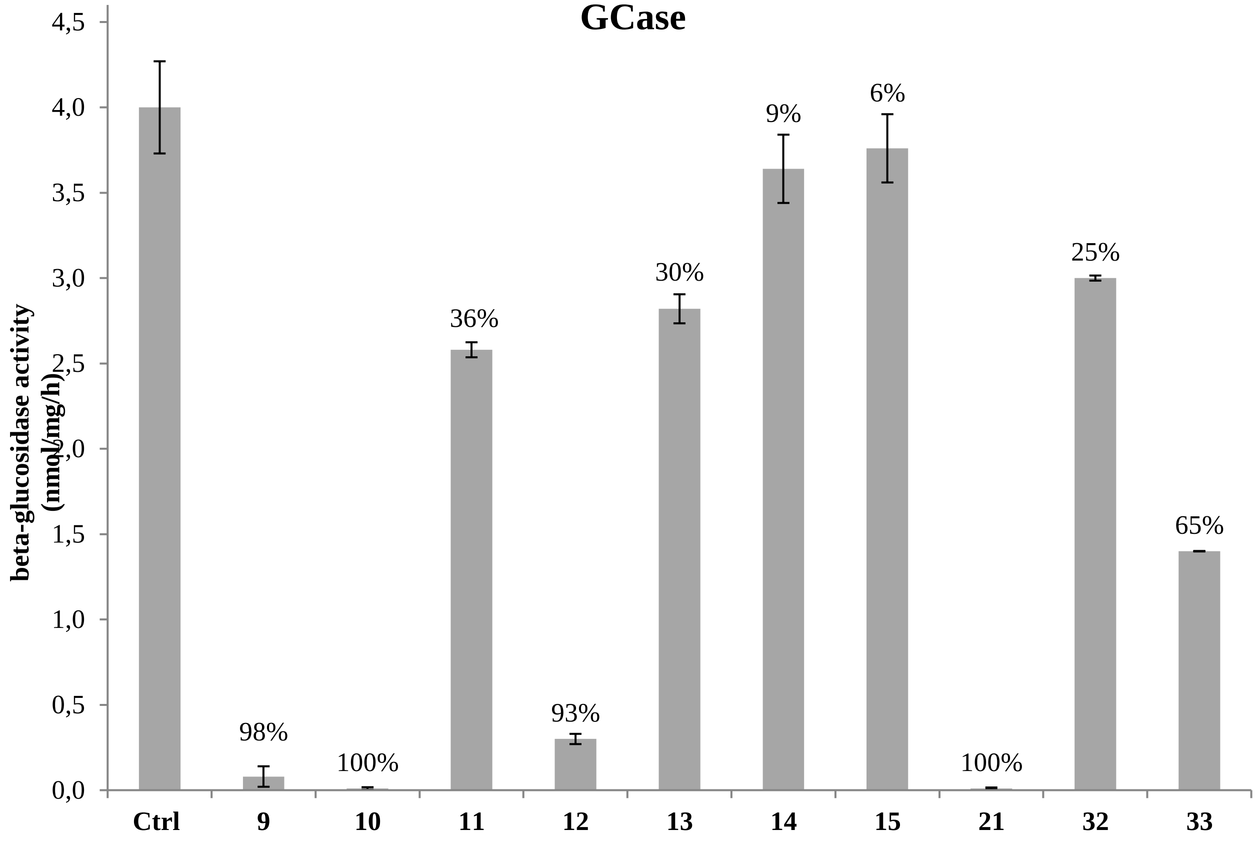

Figure S47: Percentage of GCase inhibition of the whole collection of compounds in human leukocytes extracts incubated with iminosugars at 1 mM concentration.

IC_{50} for compounds 9, 10, 12 and 21 towards human lysosomal β-glucosidase

The IC_{50} values of inhibitors against β-glucosidase were determined by measuring the initial hydrolysis rate with 4-methylumbelliferyl-β-D-glucoside (3.33 mM). Data obtained were fitted to the following equation using the Origin Microcal program.

$$\frac{Vi}{Vo} = \frac{Max - Min}{1 + \left(\frac{x}{IC_{50}}\right)^{slope}} + Min$$

where V_i/V_o, represent the ratio between the activity measured in the presence of the inhibitor (V_i) and the activity of the control without the inhibitor (V_0), "x" the inhibitor concentration, Max and Min, the maximal and minimal enzymatic activity observed, respectively.

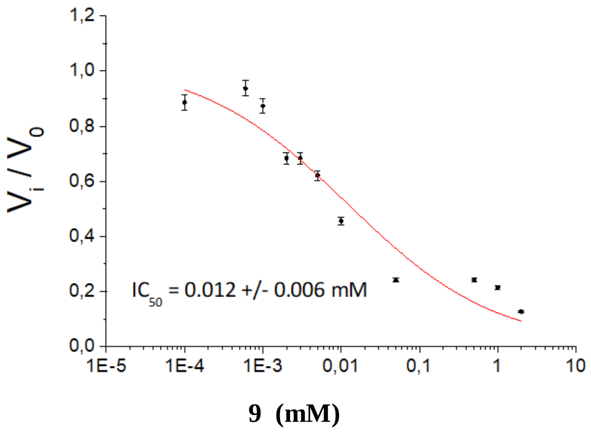

Figure S48: IC_{50} of compound 9 towards GCase

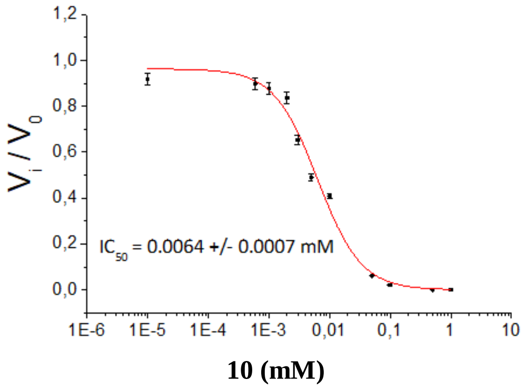

Figure S49: IC_{50} of compound 10 towards GCase

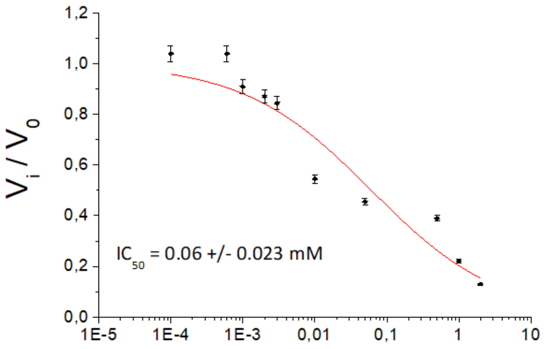

12 (mM)

Figure S50: IC_{50} of compound 12 towards GCase

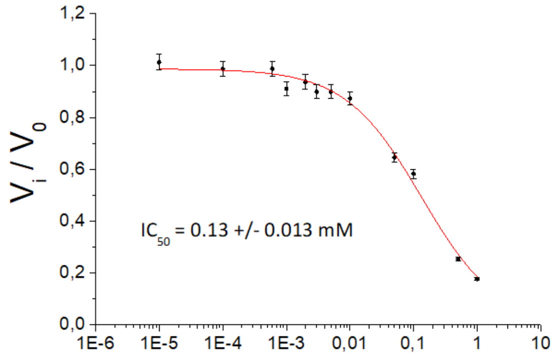

21 (mM)

Figure S51: IC_{50} of compound 21 towards GCase

Pharmacological chaperoning activity

Fibroblasts with the N370S/RecNcil mutation from Gaucher disease patients were obtained from the "Cell line and DNA Biobank from patients affected by Genetic Diseases" (Gaslini Hospital, Genova, Italy). Fibroblasts cells (20.0 x 10^4) were seeded in T25 flasks with DMEM supplemented with fetal bovine serum (10%), penicillin/streptomycin (1%), and glutamine (1%) and incubated at 37 °C with 5% CO_2 for 24 h. The medium was removed, and fresh medium containing the compounds **9**, **10** or **12** at different concentrations (10 nM, 100 nM, 1 µM, 10 µM, 50 µM, 100 µM) was added to the cells and left for 4 days. The medium was removed, and the cells were washed with PBS and detached with trypsin to obtain cell pellets, which were washed four times with PBS, frozen and lysed by sonication in water. Enzyme activity was measured as reported above. Reported data are mean S.D. (n=2).

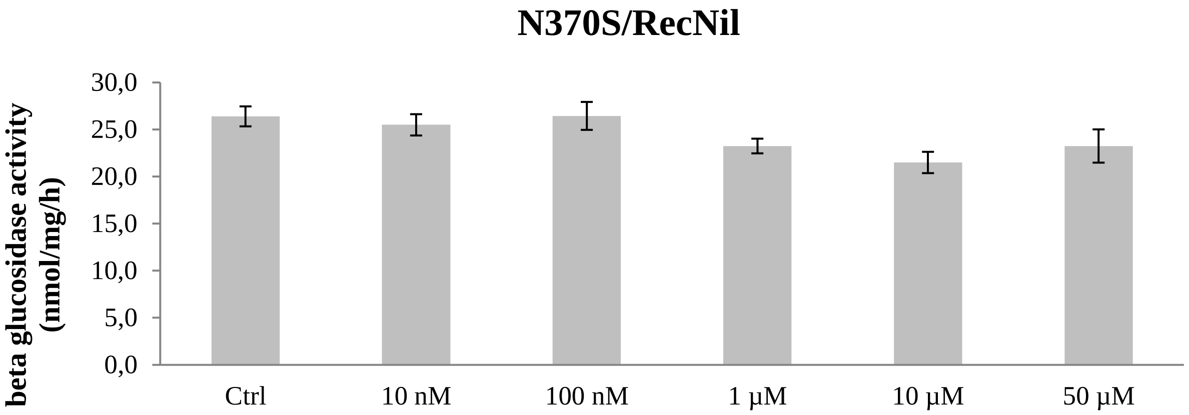

Figure S52: GCase activity in human fibroblasts derived from GD patients bearing N370/RecNcil mutations in the presence of compound **9**.

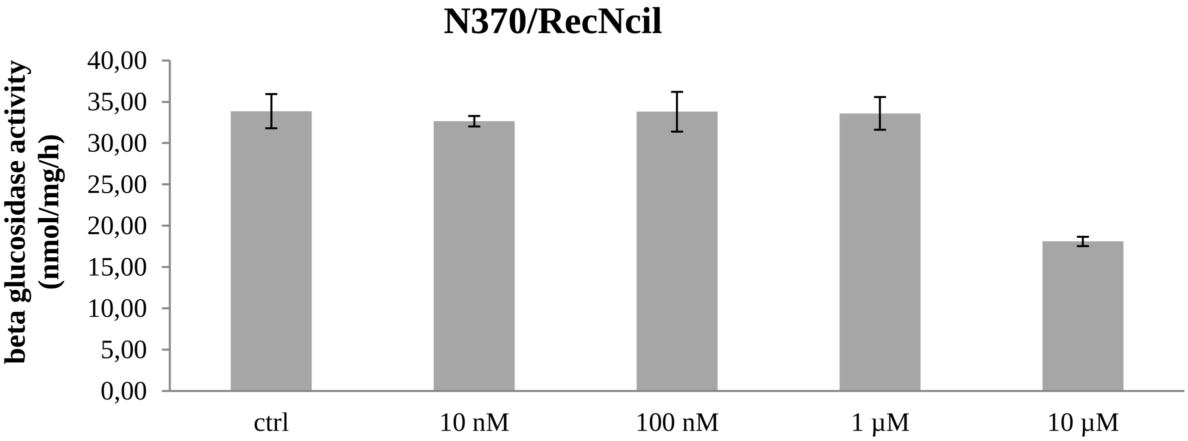

Figure S53: GCase activity in human fibroblasts derived from GD patients bearing N370/RecNcil mutations in the presence of compound **10**. The flasks containing cells incubated with 100 µM and 50 µM concentrations showed low cell viability that hampered to proceed with the assay.

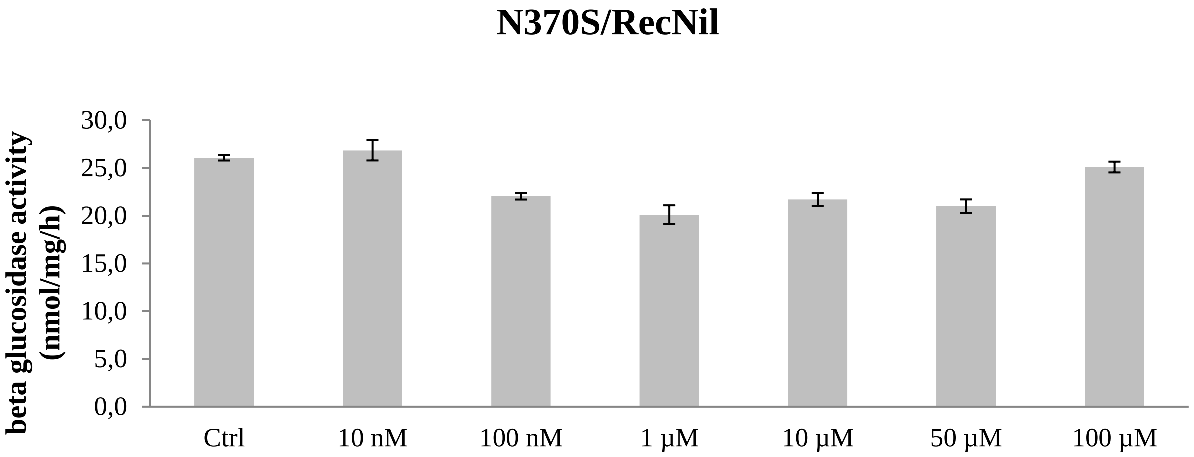

Figure S54: GCase activity in human fibroblasts derived from GD patients bearing N370/RecNcil mutations in the presence of compound **12**.
